# An Adaptive Palladium Single-Atom Catalyst Enabling Reactivity Switching between Borylation and C–C Coupling

**DOI:** 10.1021/jacs.4c17943

**Published:** 2025-05-23

**Authors:** Vitthal B. Saptal, Clara Saetta, Adriana Laufenböck, Martin Sterrer, Ik Seon Kwon, Andrea Lucotti, Matteo Tommasini, Ondřej Tomanec, Aristides Bakandritsos, Giovanni Di Liberto, Gianfranco Pacchioni, Gianvito Vilé

**Affiliations:** † Department of Chemistry, Materials, and Chemical Engineering “Giulio Natta”, 18981 Politecnico di Milano, Piazza Leonardo da Vinci 32, 20133 Milano, Italy; ‡ Department of Materials Science, 9305 University of Milan Bicocca, Via Roberto Cozzi 55, 20125 Milano, Italy; § Institute of Physics, 27267 University of Graz, Universitätsplatz 5, 8010 Graz, Austria; ∥ Department of Energy Science and Engineering, 65450 Kunsan National University, 558 Daehak-ro, 54150 Gunsan-si, Republic of Korea; ⊥ Regional Centre of Advanced Technologies and Materials, Czech Advanced Technology and Research Institute (CATRIN), 48207 Palacký University Olomouc, Šlechtitelů 241/27, 783 71 Olomouc-Holice, Czech Republic; # Nanotechnology Centre, Centre of Energy and Environmental Technologies, VŠB-Technical University of Ostrava, 17. Listopadu 2172/15, 708 00 Ostrava-Poruba, Czech Republic

## Abstract

The development of single-atom catalysts (SACs) with site-specific and tunable catalytic functionalities remains a highly desirable yet challenging goal in catalysis. In this study, we report a SAC featuring anisotropic coordination cavities synthesized via a one-step polymerization of 2,6-diaminopyridine and cyanuric chloride. These cavities provide a robust framework for anchoring isolated Pd single atoms with exceptional stability. The unique broken symmetry of the catalyst’s local structure enables precise control over reaction pathways, allowing reactivity to be switched between distinct catalytic outcomes. Specifically, under tailored reaction conditions, the catalyst can either halt at the borylation step or proceed seamlessly to Suzuki coupling in a self-cascade process. Mechanistic studies unveil the pivotal role of Pd single atoms in driving key steps, including oxidative addition, base exchange, and reductive elimination. Furthermore, green metrics demonstrate the process’s sustainability, with minimized waste generation and reduced reliance on hazardous reagents in the self-cascade transformation. This work establishes an innovative benchmark in the field of single-atom catalysis: by enabling complex, multistep transformations via strategic activation of multiple functional groups, this catalyst exemplifies the potential of self-cascade processes to revolutionize synthetic chemistry via catalysis engineering.

## Graphical abstract

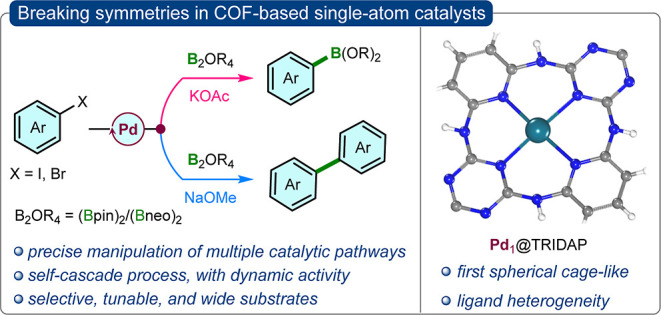

## Introduction

Catalysts play a key role in the production of essential chemicals, materials, and fuels, thereby driving efficiency and sustainability across diverse industries.^1^ The recent emergence of SACs within the spectrum of catalytic materials has attracted significant attention from the research community. These materials, featuring an atomic dispersion of transition metal atoms on a heterogeneous support, hold promise for bridging the gap between traditional homogeneous and heterogeneous catalysis.^2–5^ Over the past decade, SACs have demonstrated remarkable degrees of activity and selectivity in various transformations within the energy, environmental, and fine chemical synthesis sectors due to their fully exposed active sites.^6^ Moreover, because of the highly unsaturated coordination environment of the single metal atom and altered d-band structure, these catalysts often exhibit distinct electronic properties at the nanoscale that promote efficient reactant adsorption and enhance catalytic performance.^7,8^ In recent years, a range of support materials have been employed for the isolation of single atoms, including inorganic oxides,^9^ metal–organic frameworks,^10^ carbon-based materials,^11,12^ porous organic polymers,^13^ gC_3_N_4_,^14–17^ hybrid nanostructures,^18,19^ and covalent organic frameworks.^20^ However, in these reported carriers, the reliance on a sole, regularly repeating chemical functionality (Figure 1a) often results in weak interactions with single metal atoms, making it difficult to prevent sintering or agglomeration (the clustering of atoms) under reaction conditions and leading to the leaching of active sites into solution.^21–23^ This significantly compromises the catalyst stability and long-term effectiveness, and ultimately, this results in an uncertain number and nature of active sites, with difficulties in elucidating structure–reactivity relationships.

**Figure 1:**
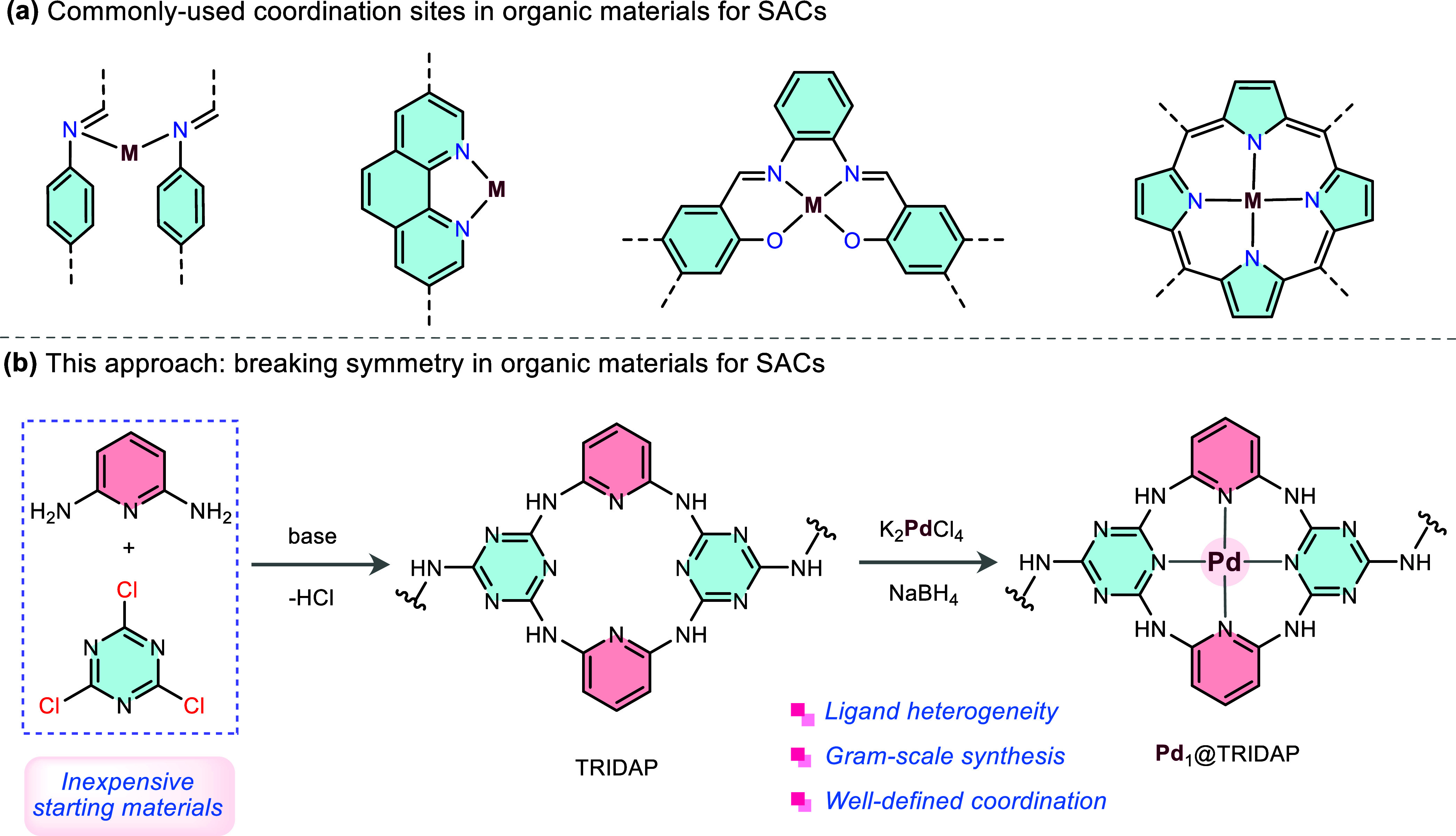
Approaches to ligand design. (a) Reported coordination sites in organic materials for the stabilization of SACs. These approaches predominantly utilize the same repeating unit. This symmetry in organic materials coordination chemistry constrains ligand design, potentially limiting the tunability and performance optimization of SACs. (b) This work breaks this symmetry for the stabilization of SACs in organic materials, enabling novel and enhanced catalytic properties.

An alternative approach to overcoming these challenges lies in the rational design of supports that can more effectively stabilize metal species. This can be achieved by developing nanostructures composed of lightweight elements like C, N, and O, which offer highly porous, crystalline structures with customizable functionalities, along with a structurally well-defined electronic environment for the effective isolation of single atoms.^24–26^ Literature reports have particularly shown that isolated metals tend to aggregate into nanoparticles when supported on organic material surfaces.^27,28^ The challenge in coordinating single precious metal atoms on organic materials arises from the lack of spatial multidentate ligand sites, primarily imine linkages.^29,30^ Such spatial ligands are crucial for maximizing metal coordination efficiency, preserving geometric integrity, and enabling precise control over the coordination environment.^31,32^ Organic materials have recently been designed with chelating ligands such as bipyridine,^33–37^ phenanthroline,^38,39^ porphyrin,^40,41^ and metallosalen,^42–44^ among others.^45–47^ However, although numerous functional organic materials have been designed, they still exhibit regularly interspaced repeating motifs, leading to electronically similar environments due to homogeneity in ligand design.^48–51^ Hence, the rational design of nanostructures that break symmetry in their structure (Figure 1b), along with their application in supporting in a stable manner single metal atoms, is yet to be accomplished.

In this work, we present a one-step method for the facile synthesis of a novel organic material that integrates triazine-linked and pyridine (TRIDAP) functionality to create a heterogeneous coordination environment for metal stabilization. This is achieved by utilizing cyanuric chloride and 2,6-diaminopyridine as relatively inexpensive precursors. The approach aims to modulate the electronic heterogeneity of SACs to establish a novel, robust environment for the effective isolation and stabilization of Pd single atoms. TRIDAP demonstrates high durability due to the covalent triazine framework (CTF) which imparts high structural stability and prevents Pd leaching;^52–54^ on the other hand, the pyridine functionalities enhance metal–ligand interactions,^55^ generating a varied electronic environment for precise control over catalytic activity (Figure 1b). The result is that Pd_1_@TRIDAP exhibits tunable catalytic function, facilitating borylation and/or a tandem homo- and hetero-coupling reactions in a self-cascade manner.

## Results and Discussion

The synthesis of the polymeric 1,3,5-triazine-linked 2,6-diaminopyridine network (TRIDAP) polymeric organic material was accomplished through a one-step polymerization process, which led to the fabrication of a stable triazine framework with precisely tailored functionalities. In particular, the polymerization occurred between 2,6-diaminopyridine and cyanuric chloride as the building blocks under mild conditions. The Pd atoms were then uniformly dispersed within the TRIDAP matrix through sonication and impregnation. Detailed procedures and specific reaction conditions are provided in the Materials and Methods section. Before catalytic experiments were conducted, the properties of the powdered materials were analyzed by using a comprehensive set of bulk and surface characterization techniques.

Fourier-transform infrared (FTIR) spectroscopy was performed to analyze the bonding structure of the materials (Figure 2a). The IR spectrum of TRIDAP presented a broad peak between 3500 and 2750 cm^–1^ corresponding to N–H stretching vibrations,^56^ which indicates the presence of amine groups and hydrogen bonding. The peak at 1617 cm^–1^ corresponds to C=N stretching in the triazine ring, the peak at 1314 cm^–1^ is attributed to C–N stretching in the triazine ring,^57^ while 1562 cm^–1^ for C=N of pyridine stretching.^58^ Finally, the signal at 1429 cm^–1^ corresponds to C=C stretching characteristic of aromatic rings.^59^ These features confirm that the polymerization process effectively produced the desired TRIDAP structure, with key bonding characteristics such as N–H stretching, C=N, and C–N stretching in the triazine ring, and C=C stretching in the aromatic rings, essential for the structural integrity and functionality of the catalyst. The FTIR spectrum of TRIDAP was compared to those of its precursors, 2,6-diaminopyridine and cyanuric chloride, to verify the successful retention of key functional groups (highlighted in yellow) and the structural integrity and functionality of the synthesized material. The relatively small red and blue shifts are consistent with the formation of new covalent bonds during polymerization, which leads to changes in the local environment of the functional groups within the extended framework of TRIDAP. However, all peaks remain within the expected IR regions. We subsequently examined the ^13^C solid-state NMR spectrum of TRIDAP to pinpoint the distinct coordination of carbon atoms within the pyridinium and triazine rings (Supporting Information, Figure S1a). The signal at ca. 109 ppm is associated with the C4 carbon of the pyridyl ring, while the peaks at 140 and 144 ppm are attributed to the C3 and C5 carbons, respectively.^60^ The peak at ca. 150 ppm corresponds to the C2 and C6 carbons of the pyridyl ring. For the triazine ring, the peaks at 163 and 170 ppm are consistent with carbon atoms within triazine carbon backbones.^57^ For the ^15^N solid-state NMR (Figure S1b), the peak at 184.8 ppm is consistent with nitrogen atoms in triazine rings, and the 124.5 ppm signal could be attributed to nitrogen in pyridine-like structures. Finally, a prominent peak at 8 ppm in the ^1^H NMR spectrum (Figure S1c) is indicative of aromatic protons on the pyridinium ring.^58^ This data further confirmed the expected structure and chemical environment of the support. Elemental C, N, and H analysis showed that the final SAC has a C/N ratio of approximately 1.34 (Table S1). The presence of Pd was confirmed by inductively coupled plasma optical emission spectroscopy (ICP–OES), showing a Pd content of 2.76 wt %. Moreover, N_2_ physisorption experiments confirmed the porous nature of the sample (Table S1).

**Figure 2:**
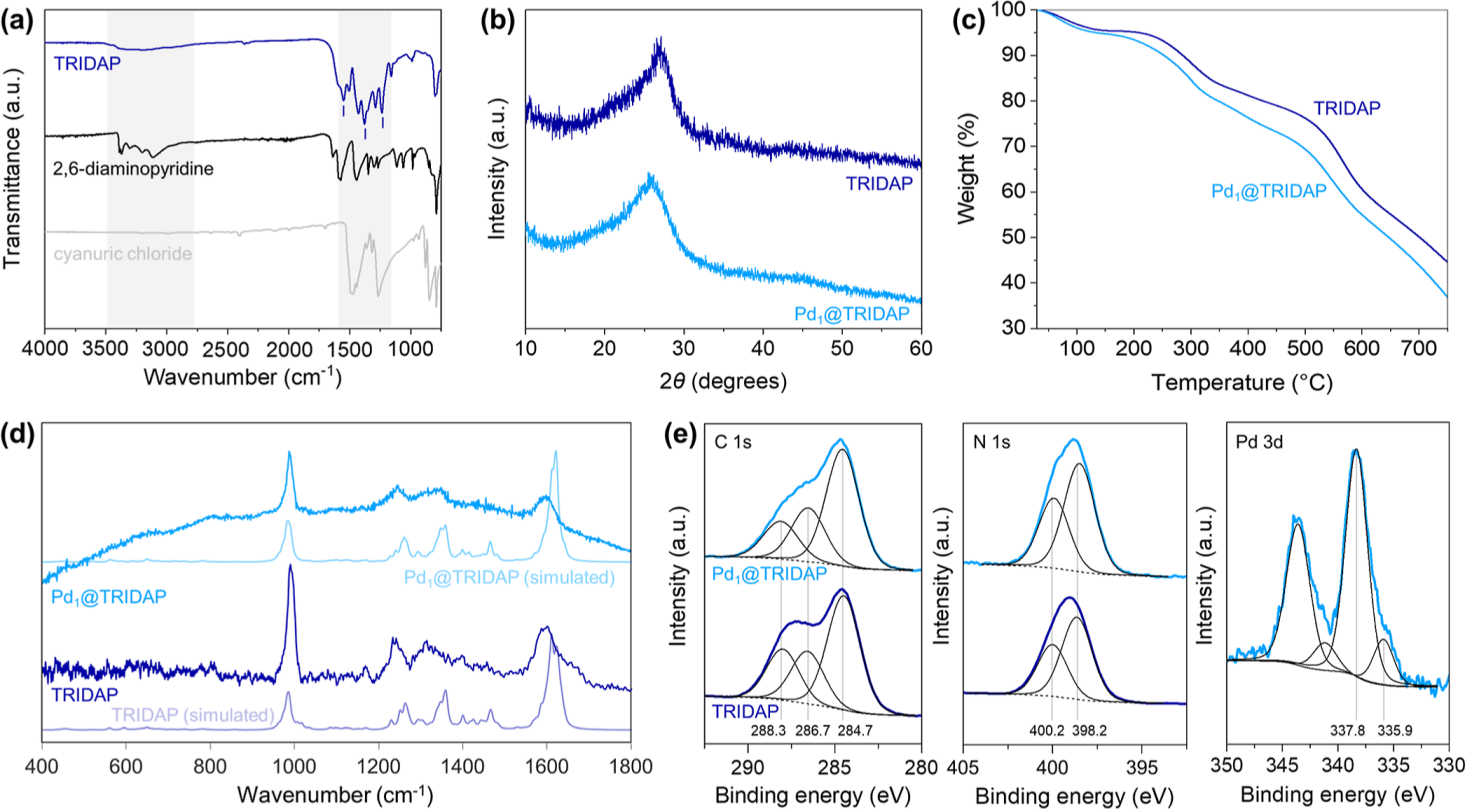
Structural, thermal, and spectroscopic characterization of Pd_1_@TRIDAP. (a) FTIR spectra of TRIDAP (dark blue), 2,6-diaminopyridine (black), and cyanuric chloride (gray). (b) XRD of TRIDAP (dark blue) and Pd_1_@TRIDAP (light blue). (c) TGA of TRIDAP (dark blue) and Pd_1_@TRIDAP (light blue). (d) Experimental Raman spectra of TRIDAP (dark blue) and Pd_1_@TRIDAP (light blue) and comparison with the DFT-computed Raman spectra of the corresponding molecular models of TRIDAP (violet-blue line) and Pd_1_@TRIDAP (azure line). (e) C 1s, N 1s, and Pd 3d XPS of TRIDAP (dark blue) and Pd_1_@TRIDAP (light blue) and corresponding peak fittings (black).

The crystalline structure of the material was assessed by X-ray diffraction (XRD) (Figure 2b). The XRD pattern of TRIDAP revealed a main reflection centered at 26.2°. The broadness and position of this peak are consistent with the *d*-spacing associated with the polymer backbone and its interplanar distances. Similar observations were made in the Pd-loaded materials, where the same peak was observed without any additional reflections, indicating the absence of any detectable Pd crystallites. Thermogravimetric analysis (TGA) of TRIDAP and Pd_1_@TRIDAP confirms excellent thermal stability up to 300 °C (Figure 2c). The initial ∼5% weight loss at ∼152 °C is due to the desorption of adsorbed water, residual solvent, and weakly bound species. The slightly higher weight loss for Pd_1_@TRIDAP suggests Pd promotes ligand decomposition, influencing oxidation under air. Major weight loss (>300 °C) corresponds to organic framework combustion, with exothermic events at 357.61 °C, 447.61 °C, and 637 °C, indicating progressive ligand breakdown.^61,62^ Both materials remain structurally intact up to 300 °C, decomposing only at higher temperatures.

To further probe the catalyst structure, we employed Raman spectroscopy on TRIDAP and Pd_1_@TRIDAP (Figure 2d). The similar spectral features between the two samples indicate that the incorporation of Pd, despite its interaction with N species (vide infra), does not significantly alter the molecular structure of the polymer. Specifically, the peak at ca. 1000 cm^–1^ corresponds to the in-plane ring deformation of variously substituted six-membered rings,^63^ while the bands around 1200–1400 cm^–1^ are attributed to collective C–C, C–N stretching, and in-plane C–H bending vibrations. Finally, the feature at ca. 1600 cm^–1^ is associated with ring-stretching vibrations.^64^ The close agreement between the DFT-simulated Raman spectra, reconstructing the vibrational characteristics of the catalyst models discussed in the next section, and the experimental data supports the accuracy of the structural models and confirms the presence of the expected vibrational modes. Moreover, the negligible difference between the Raman spectra of TRIDAP and Pd_1_@TRIDAP suggests that Pd is well-dispersed in a single-atom form.

To characterize the valence and electronic states of the metal, X-ray photoelectron spectroscopy (XPS) was recorded (Figure 2e). The C 1s spectra of both Pd_1_@TRIDAP and its metal-free counterpart display similar peaks at 284.7 eV (aromatic C–C), 286.7 eV (C–N), and 288.3 eV (C=N),^65^ indicating the presence of aromatic rings with nitrogen-containing groups, such as pyridine and triazine. The N 1s spectra further show a broad signal in both samples, which can be deconvoluted into its features at 398.2 eV (amine) and 400.2 eV (aromatic CN),^66^ confirming the presence of nitrogen species in both the metal-containing and metal-free samples. Finally, the Pd 3d spectrum of Pd_1_@TRIDAP has a main Pd 3d_5/2_ component at a 337.8 eV binding energy (BE) with a minor shoulder at 335.9 eV. The Pd peak at 337.8 eV indicates that Pd atoms are in a positively charged state.^65^ The low BE component at 335.9 eV, instead, is not present at the beginning of the analysis and progressively increased in intensity with prolonged X-ray exposure of the sample, which is attributed to beam-induced agglomeration under ultrahigh vacuum experimental conditions.

The morphology of the synthesized sample was analyzed by using microscopy (Figure 3). The TRIDAP covalent triazine-pyridine framework support exhibits a spherical morphology with both isolated and intercalated spheres observed (Figure S2). This distinctive structure likely arises from the convex curvature of the 2,6-diaminopyridine ligand connecting to cyanuric chloride, facilitating the formation of interconnected and agglomerated spheres. To gain deeper insights into the material’s morphological and compositional characteristics, high-resolution imaging and elemental analysis were performed. Figure 3a presents an aberration-corrected high-angle annular dark-field scanning transmission electron microscopy (HAADF-STEM) image, which confirms the presence and uniform distribution of single atoms in the samples. Figure 3b,c displays TEM and scanning electron microscopy (SEM) images, respectively, validating the spherical morphology of the particles, while Figure 3d shows STEM–EDS elemental mapping, illustrating the homogeneous dispersion of C, N, and Pd throughout the TRIDAP support.

**Figure 3:**
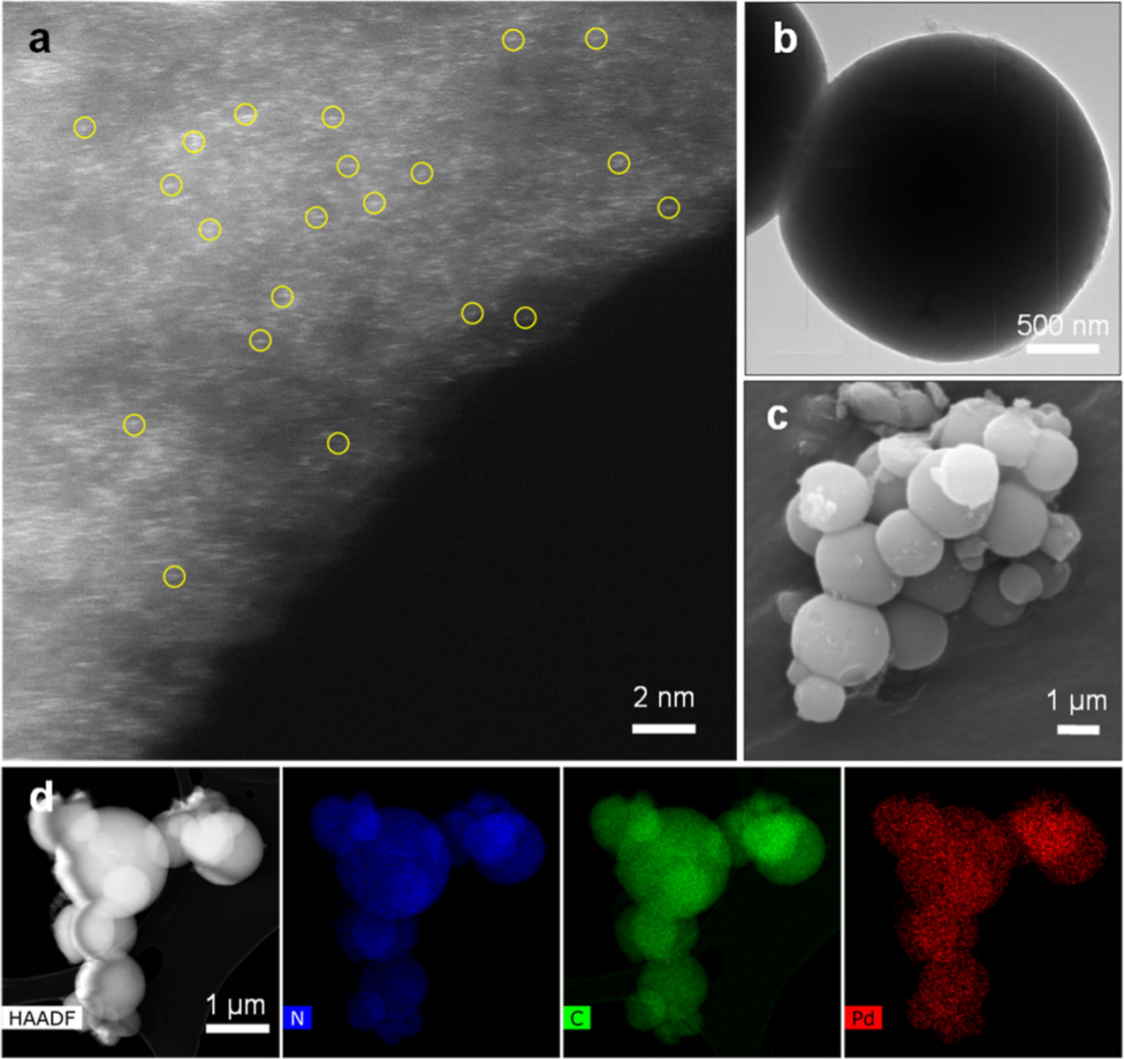
Morphological and compositional characterization of Pd_1_@TRIDAP: (a) HAADF-STEM image. Selected single atoms are marked with yellow circles. (b) TEM image at a magnification of 500 nm and (c) SEM image, showing the characteristic spherical morphology of TRIDAP. (d) STEM–EDS and corresponding elemental mapping of N, C, and Pd.

The nitrogen adsorption–desorption isotherms of TRIDAP and Pd_1_@TRIDAP were analyzed to evaluate their textural properties (Figure S3). TRIDAP exhibits a typical Type IV isotherm with a pronounced hysteresis loop at high relative pressure (*P*/*P*_0_ ∼ 0.8–1.0), characteristic of mesoporous materials. The Brunauer–Emmett–Teller (BET) analysis reveals that TRIDAP possesses a surface area of 34.158 m^2^/g, a total pore volume of 0.220 cm^3^/g, and an average pore diameter of 25.791 nm (Table S2). These values confirm the highly porous structure of TRIDAP, which offers substantial surface accessibility. However, upon Pd loading, significant changes are observed in the textural parameters. The BET surface area of Pd_1_@TRIDAP decreases to 18.078 m^2^/g, while the total pore volume reduces to 0.086 cm^3^/g, and the average pore diameter narrows to 19.073 nm. These reductions indicate the successful incorporation of Pd species, which partially block the mesopores while retaining the overall mesoporous framework. The Barrett–Joyner–Halenda (BJH) analysis further supports this, showing a decrease in the pore size and volume. The retained Type IV isotherm confirms that Pd species are well-dispersed without causing structural collapse, ensuring the material’s suitability for catalytic applications.

X-ray absorption spectroscopy (XAS) was employed to analyze the local nanostructure around the Pd metal center in Pd_1_@TRIDAP. The X-ray absorption near edge structure (XANES) for the Pd K edge in Figure 4a shows distinct rising edges for Pd_1_@TRIDAP, which are different from those of PdO powder and Pd foil. This suggests that the catalyst has a structure that differs from Pd foil and PdO. Figure 4b displays the first derivative curve of the XANES data, which was used to determine the average oxidation state of Pd atoms in the catalysts by comparing them to reference samples such as Pd(0) foil and PdO. A linear regression was done by analyzing the maximum point of the curve near the rising edge, allowing for the determination of oxidation states. The calculated oxidation state for Pd_1_@TRIDAP is +1.1. Furthermore, the absence of a sharp PdO increase near 24,362 eV is absent in the Pd SAC, indicating that no PdO-related nanoparticles exist. In Figure 4c, the nonphase-corrected extended X-ray absorption fine structure (EXAFS) data offered preliminary insights into the interatomic distance around the Pd atoms. Pd_1_@TRIDAP showed a contribution at 1.54 Å, assigned to Pd–N interatomic distance.^44^ This suggests that the Pd atoms are primarily bonded to N atoms of the TRIDAP supports, with no clear peaks related to Pd–Pd bonds observed. In the case of PdO powder, the Pd–O peak was observed near 1.52 Å, which is quite similar to that of the Pd–N bond in Pd_1_@TRIDAP, in addition to a second peak above 2.5 Å, indicating Pd–Pd interatomic distances for PdO (and Pd foil). Based on these observations, it can be concluded that there are no Pd–Pd-related bonds in the SACs. We also scrutinized the structural characteristics surrounding the nitrogen atom in the Pd_1_@TRIDAP SAC by means of the N K edges (Figure 4d). A peak near 399 eV was observed, indicating the electronic transition from N 1s orbital to π* transition of pyrrolic and pyridinic nitrogen originating from the TRIDAP supports.^67^ Notably, the peak intensity of graphitic nitrogen was weaker than that of pyrrolic/pyridinic nitrogen due to the preliminary nitrogen form in TRIDAP being pyridinic with unpaired electrons. EXAFS fitting analysis was conducted to obtain quantitative values from measured EXAFS data, such as interatomic distance, Debye–Waller factor, and coordination number, as summarized in Figure 4e (see also the Supporting Information, Table S3 and Figure S4). The coordination number for the Pd–N bond is approximately 4, consistent with the nature of the SACs with a M–N4 structure. The EXAFS peak around 2.12 Å was fitted with Pd–C/N paths rather than any kind of Pd–Pd path. The wavelet-transformed EXAFS (WT-EXAFS) was finally utilized to present the distribution of the wavelet that makes up the EXAFS data in both *k* space and *R* space (Figure 4f–h). This is because it is known that the wavelet of the *k* space tends to be located in a low/higher *k* space region if the central atoms are bonded with lighter/heavier elements. In Figure 3f, the prominent maxima around 4.7 Å^–1^ in *k* space, indicating the Pd–N bond, are observed with distinct features without any other noticeable maxima point. This result means that Pd atoms in the Pd_1_ catalyst are mainly surrounded by N atoms, in agreement with the EXAFS data. On the other hand, for both cases of PdO powder and Pd foil in Figure 3g,h, the maxima region is formed at 8–10 Å^–1^ in *k* space, implying that Pd–Pd bonds exist in PdO and Pd foil.

**Figure 4:**
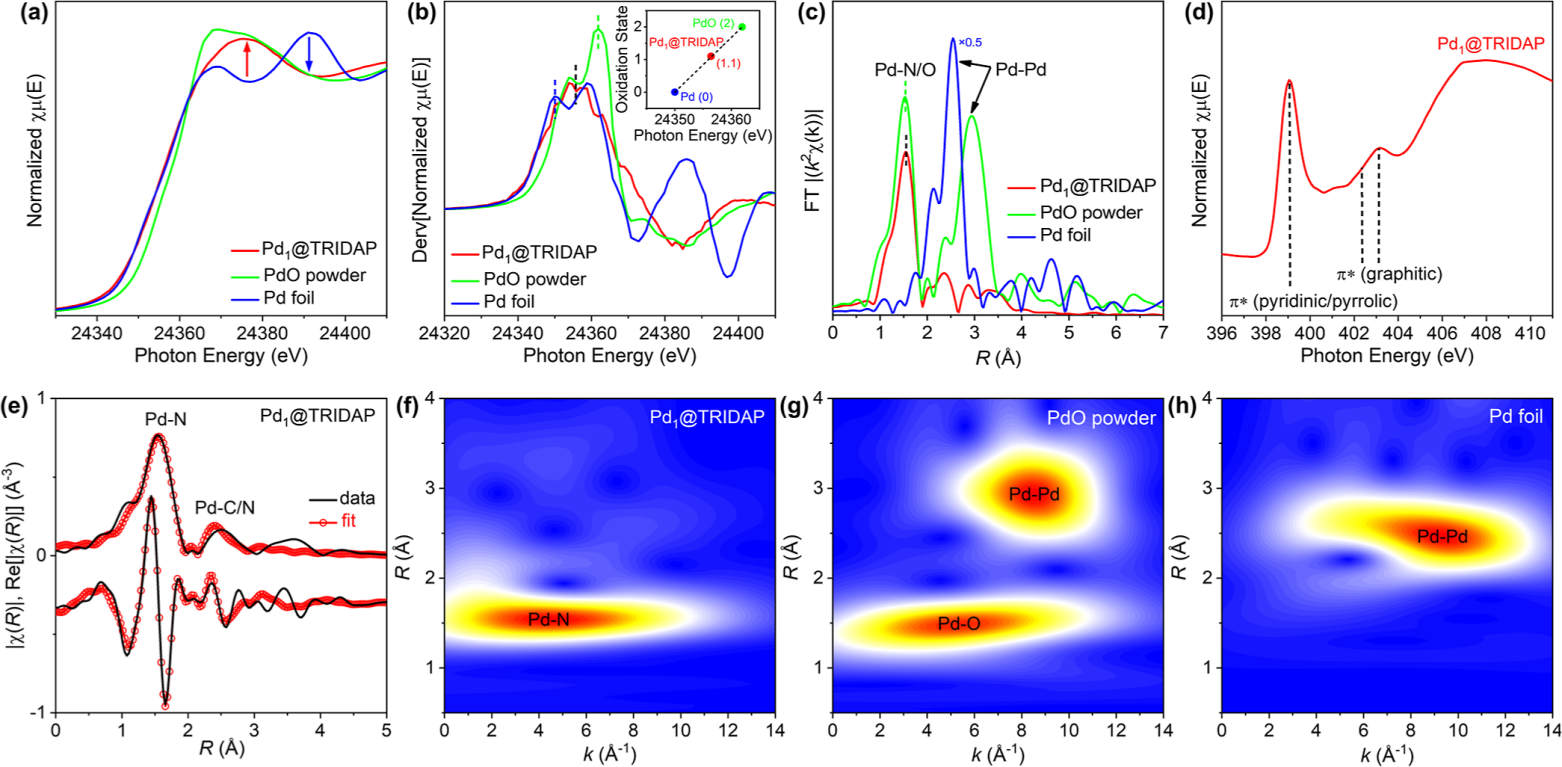
Analysis of the local coordination of Pd_1_@TRIDAP. (a) Normalized Pd K-edge XANES, (b) first derivative XANES, (c) nonphase-corrected Fourier transform EXAFS of Pd_1_@TRIDAP and Pd-containing reference materials, (d) normalized absorption spectra of Pd_1_@TRIDAP, (e) EXAFS fitting curve applied to the Fourier-transform EXAFS spectra of Pd_1_@TRIDAP, and WT-EXAFS analysis for (f) Pd_1_@TRIDAP, (g) PdO powder, and (h) Pd foil.

### Simulated Pd_1_@TRIDAP

To derive the local structure of Pd_1_@TRIDAP, we began with the compositional data of the catalyst (Table S1), calculating the average ratio between triazine-like and pyridine-like building blocks. This was used to propose a plausible structure for the TRIDAP support. To do so, we solved a linear mathematical system using the results from the elemental analysis, and details on the calculation are provided in the Supporting Information. The solution of the problem led to a triazine/pyridine ratio of 0.4, corresponding to a C/N ratio equal to 1.2, in good agreement with the experimental value of 1.25. A model structure compatible with this condition is shown in Figure S5 and depicts a spherical particle with 3-fold cavities available for metal atoms (herein, 3N-TRIDAP). An alternative structure is reported in Figure 5b, where the catalyst is 4-fold coordinated. We chose to simulate molecular analogues of the TRIDAP with the same cavity size and atomic arrangement because a periodic polymer structure would require extremely large computational cells, substantially increasing the size and complexity of the simulations. However, this approach does not introduce any artifacts regarding the cavity structure or the BE of the metal atom, as demonstrated in the Supporting Information, Figure S6, where we performed test calculations on the 3N-TRIDAP structure and compared the local structure and BE of the catalyst in both the molecular model and the extended periodic system. With the structure of the CTF, we proceeded to simulate the anchoring positions of the Pd single atoms. Starting from 3N-TRIDAP, the potential energy surface revealed several minima within 0.1 eV, where the Pd atom exhibited coordinations with bond lengths ranging from 2.1 Å to 2.3 Å. The lowest energy configuration featured one Pd–C and two Pd–N bonds at distances of 2.12 Å, 2.14 Å, and 2.61 Å, respectively (Figure 4b), indicating an unsaturated coordination of the metal center and a calculated oxidation state of +1, in agreement with the XAS data, and with a magnetic moment of μ_Pd_ = 0.7. The coordination environment is, however, substantially different from the measured one. In the case of 4N-TRIDAP, the Pd atom has an oxidation state of +1, with a magnetic moment of μ_Pd_ = 0.8. The coordination of Pd is equal to four, as suggested experimentally, with four Pd–N compatibles with the measured ones, with Pd–N distances in the range of 2.16–2.27 Å. We finally theoretically assessed the stability of this structure using Pourbaix diagrams for SACs, as described in one of our recent papers,^68^ and details are given in the Supporting Information, Figure S7. Specifically, Figure 5c presents the stability diagram as a function of temperature *T* and the BE of the metal to the support, *E*_b_, which predicts the stability of the Pd_1_@TRIDAP model. Once again, the structure more compatible with the experiment is 4N-TRIDAP, which we selected for the reactivity investigation.

**Figure 5:**
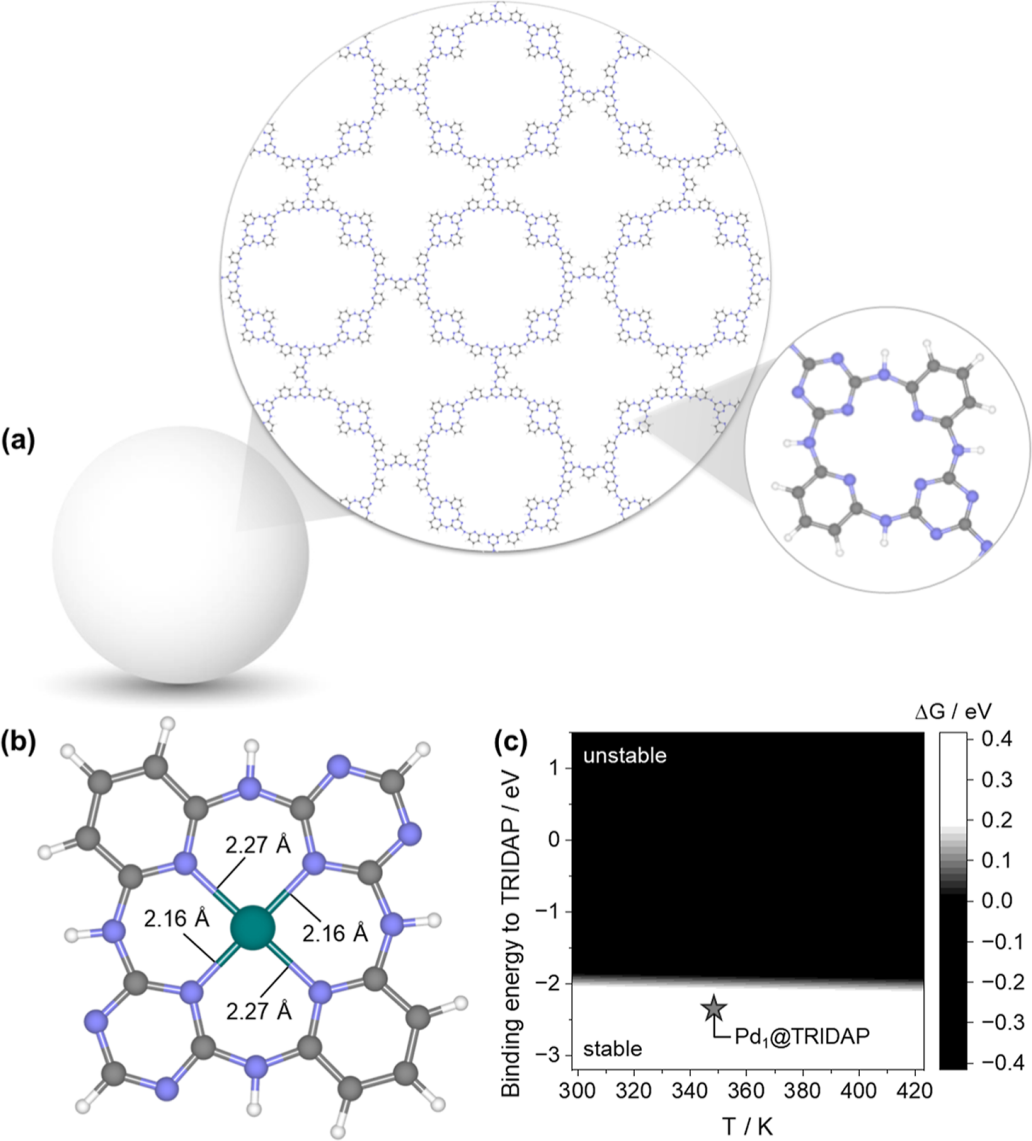
Computational models of Pd_1_@TRIDAP and predicted stability. (a) Corrugated polymer surface structure containing 4N-cavities. (b) Pd anchored to the 4N-cavity of TRIDAP and (c) stability plot of the catalyst against temperature and BE. Black areas in the stability plot indicate regions, where the modeled structure of Pd_1_@TRIDAP is not stable (Δ*G*_dissolution_ < 0 eV); white areas represent regions, where the structure is predicted to be stable (Δ*G*_dissolution_ > 0.2 eV); and gray areas indicate conditions, where the free energy for the dissolution process is close to zero, suggesting a metastable region.

### Optimization of Reaction Conditions

The as-synthesized Pd_1_@TRIDAP was employed in the Miyaura borylation of aryl halides to synthesize arylboranates, which serve as key building blocks in the construction of complex molecular architectures for drug discovery and development. The optimization of the reaction conditions commenced with iodobenzene (**1**) as the substrate, and bis­(pinacolato)­diboron (B_2_pin_2_) as the borylating reagent to obtain the arylboranate (**2a**), with NaOAc as the base and Pd_1_@TRIDAP as catalyst. The initial reaction, conducted at 90 °C for 12 h in toluene as the solvent, resulted in a yield of 20% (Table 1, entry 1). Subsequently, various solvents, including 1,4-dioxane, dimethylformamide (DMF), and water, were evaluated, yielding moderate-to-good results (Table 1, entries 2–4). Further investigations explored solvent mixtures, such as water–ethanol (38%), toluene-ethanol (24%), and dioxane-ethanol (68%) (Table 1, entries 5–7). Interestingly, polar protic solvents such as ethanol and methanol provided significantly higher yields of **2a** (76% and 82%, respectively) compared to other solvents. This suggests that hydrogen bonding or solvent coordination stabilizes reaction intermediates, while protic solvents enhance B_2_pin_2_ activation, facilitating transmetalation.

**Table 1:**
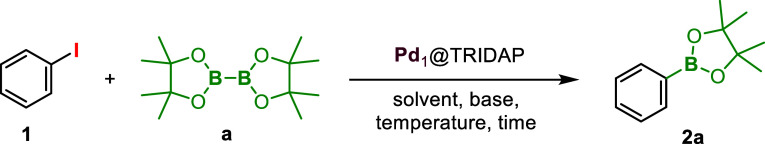
Optimization of the Reaction Conditions for the Miyaura Borylation Reaction^a^

entry	solvent	base	temperature (°C)	time (h)	yield^b^ (%)
1	toluene	NaOAc	90	12	20
2	dioxane	NaOAc	90	12	17
3	DMF	NaOAc	90	12	48
4	water	NaOAc	90	12	42
5	water–ethanol	NaOAc	90	12	38
6	toluene–ethanol	NaOAc	90	12	24
7	dioxane–ethanol	NaOAc	90	12	68
8	ethanol	NaOAc	90	12	76
9	methanol	NaOAc	90	12	82
10	methanol	K_2_CO_3_	90	12	24
11	methanol	Na_2_CO_3_	90	12	17
12	methanol	KOAc	90	12	91
13	methanol	KOAc (3)	90	9	94
14	methanol	KOAc (1.5)	90	9	83
15	methanol	KOAc (1)	90	9	46
16	methanol	no base	80	9	21
17	methanol	KOAc	90	7	90
18	methanol	KOAc	80	7	90
19	methanol	KOAc	50	7	44
20	methanol	KOAc	80	5	84
21	methanol	KOAc	80	3	69
22^c^	methanol	KOAc	80	9	5
23^d^	methanol	KOAc	80	7	60
24^e^	methanol	KOAc	80	7	51

a Unless indicated otherwise, the reaction conditions were iodobenzene (1 mmol), B_2_Pin_2_ (1.3 mmol), base (2 mmol), and solvent (5 mL).

b Isolated yields.

c Reaction performed in the absence of Pd_1_@TRIDAP catalyst.

d Reaction using Pd@C_3_N_4_.

e Reaction using Pd/C (4.64 mg of 10 wt.% Pd/C).

The choice of base was also a crucial factor in optimizing the reaction. Our tests revealed that using weak bases such as KOAc led to a significantly higher yield (91%) compared to NaOAc (82%), whereas strong bases such as K_2_CO_3_ and Na_2_CO_3_ resulted in substantially lower formation of **2a** (Table 1, entries 10–12). Notably, when KOAc was replaced with K_2_CO_3_, the yield dropped drastically from 91% to just 24%. This result suggests that strong bases may disrupt the catalytic cycle, interfering with the transmetalation step, which is essential for product formation. In contrast, KOAc plays a direct role in activating B_2_pin_2_ on the catalyst surface by acting as a ligand, facilitating its interaction with the Pd complex and enhancing transmetalation efficiency by minimizing side reactions. Subsequently, the effect of base concentration was examined (entries 13–16), and our results revealed that the presence of 2 equiv of KOAc was essential to obtain excellent yields of **2a**. In contrast, the reaction conducted with 1.5 equiv or in the absence of a base led to a very poor yield. Next, the impact of temperature was investigated, revealing a decline in yield when the reaction temperature was lowered from 90 to 50 °C (entries 17–19). A temperature of 80 °C provided an optimal yield of 90% (entry 18). Finally, the influence of reaction time was examined, revealing that a 7 h reaction duration resulted in a 90% yield of **2a**, while reducing the reaction time to 5 or 3 h led to lower yields (entries 20–21). As a control experiment, in the absence of Pd_1_@TRIDAP, only trace amounts of the product were observed (entry 22), proving that the reaction proceeds through a catalytic mechanism involving Pd. Then, the catalytic activity of Pd_1_@TRIDAP was compared with benchmark catalysts such as Pd-supported graphitic carbon nitride (Pd–C_3_N_4_) and commercial Pd/C (entries 23 and 24). Under optimized conditions, Pd@C_3_N_4_ achieved a 60% yield, while Pd/C resulted in a 51% yield of desired product **2a**. To further assess the catalytic efficiency of the synthesized catalyst, molecular Pd catalysts such as PdCl_2_ and Pd­(OAc)_2_ were also screened, yielding 33% and 45%, respectively (Supporting Information, Table S4). Finally, the effect of catalyst loading was examined, revealing that reducing the amount of catalyst resulted in lower yields of **2a**. The optimal catalyst loading was determined to be 10 mg, ensuring maximum efficiency (Figure S8a).

With the optimization reaction conditions in hand, we explored the substrate scope of our borylation method (Figure 6). The catalytic system demonstrated exceptional efficiency, yielding borylated products not only from iodobenzene (90%) but also from bromobenzene (35%) (Figures 6, **2a**). Methyl-substituted aryl iodides at the *para* and *meta* positions exhibited excellent reactivity (**2b**–**2c**), whereas *ortho*-substituted derivatives gave moderate yields due to steric hindrance (**2d**). Electron-donating functional groups such as phenol and OMe demonstrated good yields (**2e**–**2f**, 75–79%). Similarly, bromoarenes with bulky groups such as *tert*-butyl at the *para* position afforded up to 36% yield (**2g**). Electron-withdrawing groups such as CN, CHO, COMe, NO_2_, and CO_2_Me at the para position were well tolerated, delivering borylated products efficiently (**2h**–**2l**). Furthermore, diverse aryl halides, including –Cl, –F, and –CF_3_, showed excellent selectivity under the optimized conditions (**2m**–**2o**). The method also proved to be effective for aliphatic aryl halides, yielding up to 87% of the borylated product (**2p**). Finally, polycyclic aryl halides such as 1-iodonaphthalene, 4-iodobiphenyl, and 2-iodobiphenyl were successfully borylated, achieving high yields despite significant steric hindrance (**2q**–**2t**). The competitive reactivity between bromo- and iodo halides within the same molecule revealed a high selectivity for borylation at the iodo functional group (**2u**) at the optimized condition. Moreover, the successful diboration of 1,4-diiodobenzene, an important precursor, was achieved using this developed catalytic system. The scope of the designed catalyst was then investigated for bis­(neopentyl glycolato)­diboron (B_2_neo_2_) as the borylating reagent (**3a**–**3g**). A range of aryl halides underwent borylation using B_2_neo_2_ with excellent yields compared to B_2_pin_2_. DFT calculations confirmed this trend, as the use of B_2_neo_2_ in place of B_2_pin_2_ leads to negligible changes to the reaction energy profile (vide infra). Various halides such as –F, –Cl, and –CF_3_ in para position exhibited excellent tolerances, yielding the product in excellent amounts (**3a–3d**, 80–98%). Additionally, polycyclic aryl halides like 1-iodonaphthalene, 4-iodobiphenyl provided excellent yields (**3e**–**3f**). However, 2-iodobiphenyl resulted in lower yields, potentially due to more significant steric hindrance at the *ortho* position (**3g**). Next, we investigated the recyclability of the synthesized Pd_1_@TRIDAP catalyst for the Miyaura borylation reaction. The catalyst demonstrated excellent recyclability for up to six cycles with negligible loss in activity (Figure S8b). ICP–OES analysis showed no detectable Pd leaching (below 0.01 ppm), suggesting strong binding between the metal center and the triazine-pyridine support. We performed TEM and STEM analysis on fresh and recycled catalysts, observing no Pd agglomeration after six cycles (Figure S11), confirming the triazine-pyridine network’s stabilizing effect on Pd single atoms.

**Figure 6:**
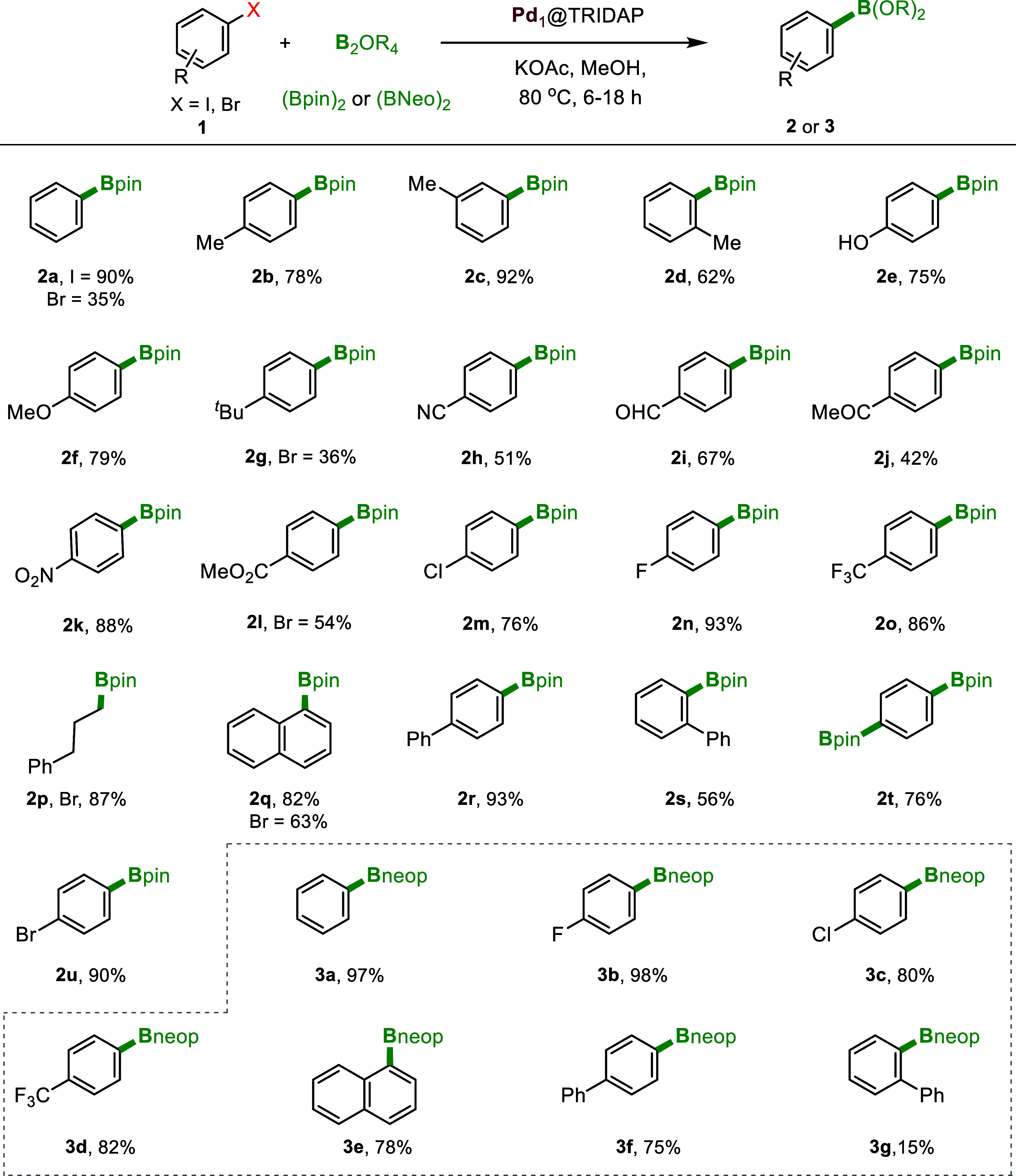
Substrate scope for the Miyaura borylation of aryl halides over Pd_1_@TRIDAP. Reaction conditions: aryl halides (0.5 mmol), diborane (0.6 mmol), KOAc (2 mmol), methanol (5 mL), and Pd_1_@TRIDAP (10 mg). All yields are isolated yields.

### Reaction Mechanism

To gain insights into the atomistic details of the reaction, we performed density functional theory (DFT) calculations to rationalize the observed trends in catalytic performances and describe the reaction mechanism for the Miyaura borylation reaction (see also Table S7). Bromobenzene was used as the aryl halide in the calculations to avoid complexities from heavy atoms like iodine and the associated need for corrections, such as spin–orbit coupling. The overall reaction is exergonic. Initially, in the presence of Pd_1_@TRIDAP (**1**), the aryl halide undergoes oxidative addition, forming metal aryl halide complex **2**, Figure 7. Subsequently, the halide ion in complex **2** is replaced by a KOAc base molecule, generating (acetato)­palladium complex **3**. This step requires overcoming a small thermodynamic barrier and is more favorable than initiating the reaction with KOAc adsorption, followed by the aryl halide addition, as shown in Figure S9. The acetate complex then interacts with B_2_pin_2_, transferring a boryl group to the Pd center and releasing a boryl acetate species. The resulting borylated complex **4** then undergoes reductive elimination, forming the final product and regenerating the catalyst (Figure 7). We did not observe the formation of an intermediate complex between **3** and **4** made by Pd, acetate, and diboron, which could be attributed to the spatial hindrance preventing the formation of the intermediate. The trends in the observed yield as a function of temperature suggest an activation energy (*E*_a_) of approximately 0.25 eV, assuming an Arrhenius-like dependence of the yield on temperature. The predicted activation energy from ab initio thermodynamics is slightly lower (0.1 eV) than expected. Furthermore, computational analyses reveal that the substitution of B_2_pin_2_ with B_2_neo_2_ does not significantly impact the reaction energy profiles, as depicted in Figure 7. This finding aligns with the trends in the reaction yields presented in Table 1. However, replacement of KOAc with K_2_CO_3_ is anticipated to inhibit the reaction. This is attributed to the inability of K_2_CO_3_ to facilitate the borylation process or the formation of a Pd carbonate complex (specifically, the Pd acetate complex) due to a substantial thermodynamic barrier, as illustrated in Figure S9. This highlights the importance of the acetate ligand in the catalytic cycle.

**Figure 7:**
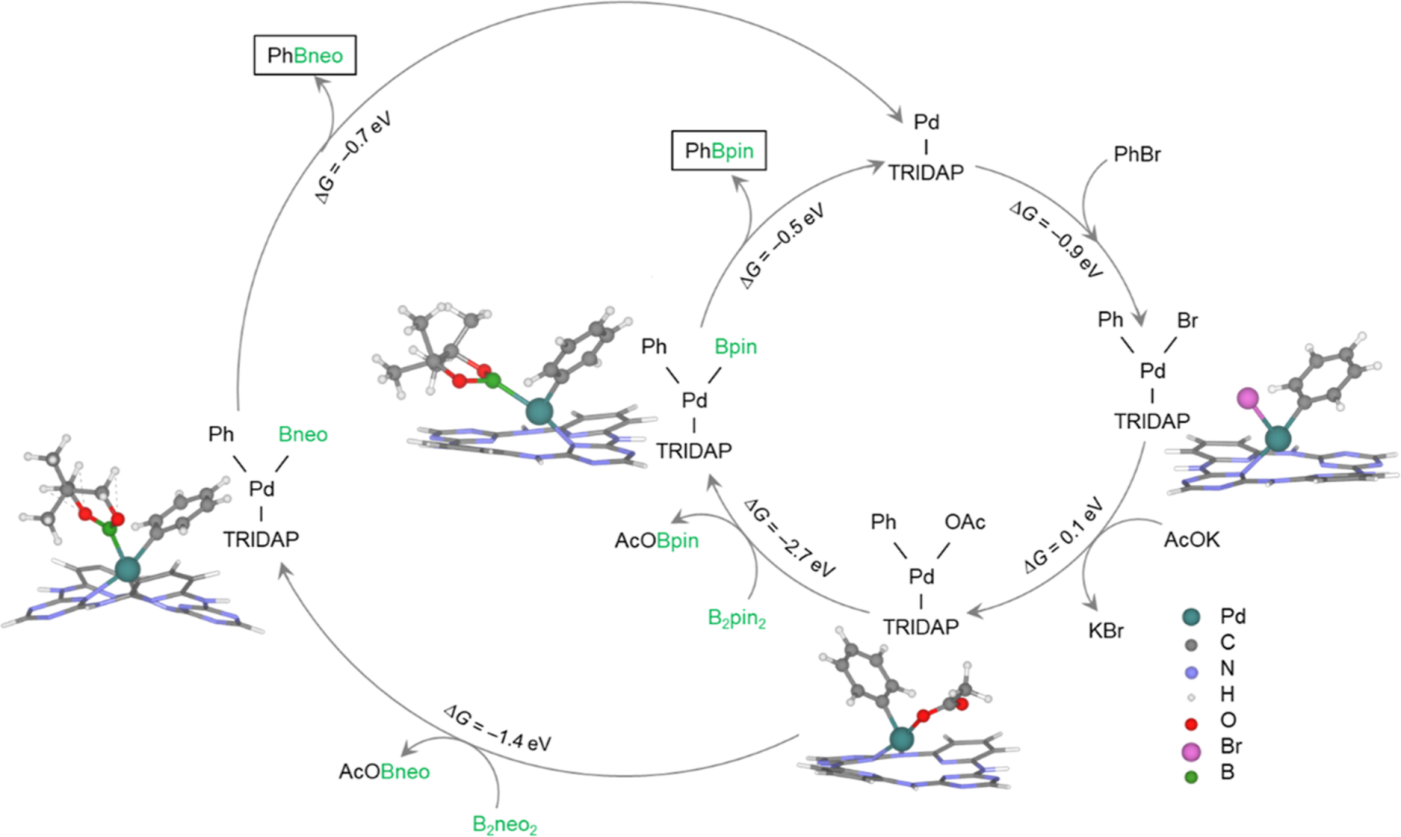
Reaction mechanism for the Miyaura borylation reaction over Pd_1_@TRIDAP. Catalytic cycle at *T* = 80 °C for Miyaura borylation reaction on Pd_1_@TRIDAP in the presence of KOAc and B_2_pin_2_ as reagents, and the variation in the catalytic cycle in the presence of B_2_neo_2_ as the borylating agent.

### Self-Cascade Coupling

Following the successful borylation of various aryl halides, we explored the potential of Pd_1_@TRIDAP to facilitate a tandem borylative Suzuki homo-coupling reaction (Figure 8). This investigation aimed to determine key factors influencing in situ coupling after borylation, identifying conditions that either promote or hinder the cascade process. In fact, a self-cascade reaction is particularly advantageous, as it integrates multiple transformations into a single synthetic step, enhancing reaction efficiency while minimizing intermediate isolation. This not only streamlines the reaction sequence but also improves overall yield and sustainability in synthetic organic chemistry (Figure 8a, Supporting Information, Scheme S1).

**Figure 8:**
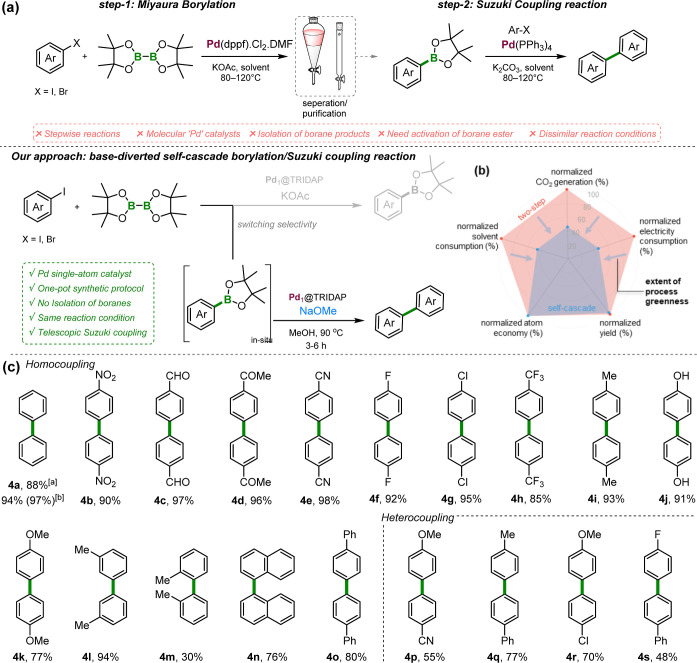
Self-cascade homo- and hetero-coupling over Pd_1_@TRIDAP. (a) Literature precedents comparing traditional coupling methods with the current self-cascade approach, highlighting its potential for more sustainable synthetic routes. (b) Sustainability assessment of the two-step synthesis versus one-step self-coupling using with Pd_1_@TRIDAP in terms of CO_2_ emissions, electrical consumption, solvent usage, yield, and atom economy. (c) Substrate scope. Reaction conditions. **1** (0.5 mmol), B_2_pin_2_ (0.25 mmol), NaOMe (1.5 mmol), MeOH (5 mL), time (4 h), and Pd_1_@TRIDAP (5 mg). ^a^The reaction was performed using KO^*t*^Bu. ^b^The reaction was performed using B_2_neo_2_. All yields are isolated yields. The reaction mechanism, which may either proceed to self-coupling or be inhibited by borylation, is illustrated in the Supporting Information, Figure S9.

In this context, several parameters were expected to influence the coupling outcome, including the Pd coordination environment, reagent concentration, and solvent choice. These factors could either enhance boron reagent activation and Pd catalysis or suppress the critical intermediate steps necessary for successful C–C bond formation. During the experimental campaign (Supporting Information, Scheme S2), we unexpectedly observed that replacing acetate bases with KOtBu or NaOMe led exclusively to the formation of the homo-coupled biphenyl product (**4a**), regardless of the borylation reagent used. Notably, B_2_neo_2_ reacted more rapidly than B_2_pin_2_, leading to a higher yield (97%), suggesting that the rate of transmetalation and the stability of the boron reagent significantly influence the reaction outcome.

DFT calculations confirmed that NaOMe not only facilitates the Miyaura borylation but can also promote the C–C coupling process (Supporting Information, Figure S10). This is due to the ability of NaOMe to stabilize the obtained transition state through its strong nucleophilic character, which lowers the energy barrier for the C–C coupling. Additionally, the coordination of NaOMe with the boron species creates a more reactive intermediate, where Pd passes from a formal oxidation state of (+I) to (+II), enhancing the likelihood of C–C bond formation (Supporting Information, Figure S10). This stabilization lowers the activation energy barrier associated with the C–C coupling step, facilitating the reaction and restoring the catalyst. In contrast, acetate, while also a nucleophile, possesses a less effective electron-donating ability due to its resonance stabilization. The negative charge is delocalized over the oxygen atoms, resulting in a reduced nucleophilic strength compared to NaOMe. Consequently, acetate cannot effectively coordinate with the boron species to form a reactive intermediate. DFT calculations reveal that no stable minimum energy structure can be found for an acetate–boron complex, indicating that acetate fails to engage with the boron species in a manner conducive to C–C bond formation. Furthermore, the acetate base lacks the capability to activate the B–O bond, as it does not stabilize the corresponding transition state. This results in a reaction pathway that is limited to borylation without progressing to the desired C–C coupling.

We then investigated a series of *para*-substituted aryl iodides with electron-withdrawing groups, including –NO_2_, –CHO, –COMe, –CN, and halides such as –F, –Cl, and –CF_3_ (Figures 8c, **4b–h**). These substrates consistently gave excellent results in the homo-coupling reaction (85–98%). Furthermore, electron-donating derivatives also tolerated the reaction conditions well, producing homo-coupled products in excellent amounts (**4i–4l**). Nevertheless, sterically hindered aryl halides like 2-methyl group-substituted ones afforded lower yield (**4m**). Next, we successfully utilized the catalyst for the synthesis of polycyclic aromatic derivatives, specifically naphthalene and biphenyl, and hetero-coupled aryl halides in a one-pot synthesis (**4p–4r**).

Notably, the synthesis of compound **4a**, specifically biphenyl, was successfully scaled up from an initial 0.5 mmol of substrate to 5 mmol. The isolated yield obtained in the scale-up process (91%) was comparable to the 88% yield obtained in the smaller-scale experiment, demonstrating the robustness of our methodology across different reaction scales.

Finally, we demonstrate the versatility and efficiency of Pd-catalyzed cross-electrophile coupling in a sequential one-pot synthesis of key pharmaceutical intermediates. This highly modular strategy enables the streamlined synthesis of complex molecules from simple electrophiles, reducing synthetic steps and improving efficiency. In Scheme 1a, the reaction begins with 1-chloro-4-iodobenzene (**1m**), which undergoes selective borylation using (Bpin)_2_ and a Pd-catalyst in the presence of KOAc. This intermediate is further coupled with 2-iodoaniline via a second electrophilic coupling step, affording 4′-chloro-[1,1′-biphenyl]-2-amine (**4t**) in an excellent yield of 82%. The product serves as a key intermediate in the synthesis of Boscalid, an industrially significant fungicide. Additionally, a literature comparison of Miyaura borylation and cascade homo-coupling using reported molecular catalysts (Table S6) highlights the superior catalytic activity and metal recovery of Pd_1_@TRIDAP.

**Scheme 1:**
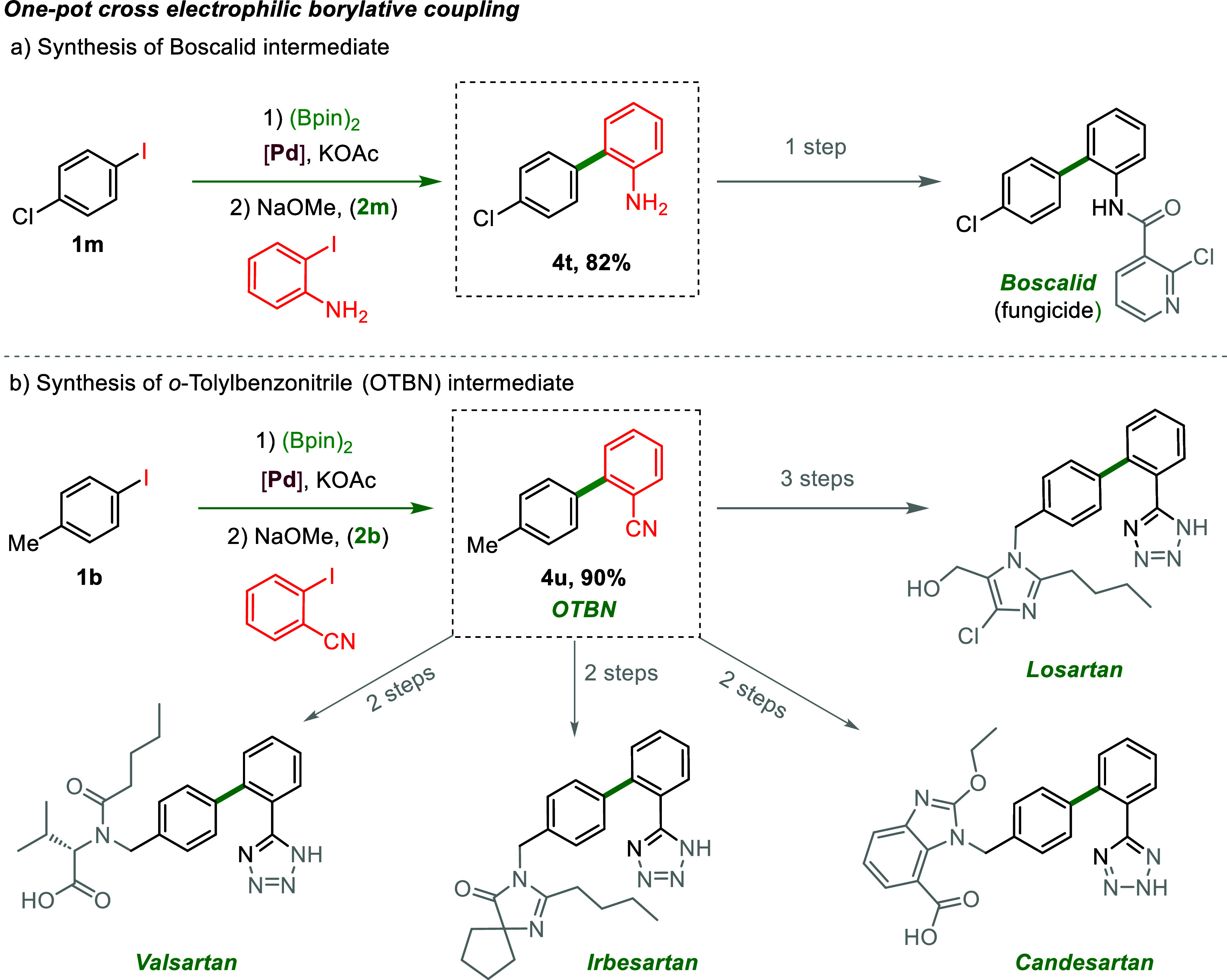
Application of Pd_1_@TRIDAP in the Sequential One-Pot Synthesis of Biologically-Active Intermediates^a^

a (a) Synthesis of Boscalid intermediate, 4′-chloro-[1,1′-biphenyl]-2-amine (**4t**), using a cross-electrophile coupling strategy. (b) Synthesis of *o*-tolylbenzonitrile (OTBN, **4u**) as an intermediate for antihypertensive drugs (Losartan, Valsartan, Irbesartan, and Candesartan). Reaction conditions: step 1: aryl halides (1 mmol), B_2_pin_2_ (1.2 mmol), KOAc (1.5 mmol), methanol (5 mL), and Pd_1_ @TRIDAP (20 mg); step 2: aryl halides (1 mmol) and NaOMe (1.5 mmol).

A similar approach is investigated in Scheme 1b for the synthesis of OTBN (**4u**) from 4-iodotoluene (**1b**) and 2-iodobenzonitrile, achieving an outstanding 90% yield. OTBN is a crucial intermediate in the synthesis of antihypertensive drugs such as Losartan, Valsartan, Irbesartan, and Candesartan. This streamlined, one-pot methodology minimizes synthetic steps, reducing operational complexity while enhancing the scalability and efficiency. The proposed cross-electrophile-coupling strategy offers significant advantages for pharmaceutical applications, providing a sustainable, cost-effective, and modular approach to accessing high-value intermediates. Given the competitive landscape of drug development, this method has immense potential in both academic and industrial settings, enabling faster development times and reduced manufacturing costs.

## Conclusions

In conclusion, we have successfully synthesized a spherical cage-type carrier based on a polymeric organic material structure that incorporates ligand heterogeneity through the use of a triazine and pyridine functionality. This material was employed for the first time to stabilize Pd single atoms, creating a highly effective SAC. Advanced characterization techniques, including TEM, XAS, Raman, and XPS, confirmed the structure and properties of the synthesized catalyst. The Pd_1_@TRIDAP catalyst demonstrated exceptional catalytic activity for the selective Miyaura borylation reaction of a wide range of aryl halides with diboranes. It also showed remarkable tolerance toward various aryl halides and diboranes under mild reaction conditions. Furthermore, this catalyst facilitated tandem homo- and hetero-Suzuki coupling reactions in a self-cascade process with excellent isolated yields. The high stability and excellent recyclability of the catalyst were also demonstrated, and the catalyst structure and reactivity were rationalized through first-principles calculations. These findings highlight the potential of rationally designed SACs for advanced applications in organic synthesis. In fact, by engineering catalytic materials that enable multiple bond-forming steps within a single reaction framework, this paves the way for streamlined synthesis, reduced reaction times, and increased overall yields, thus leading to a greener and more sustainable chemical synthesis. In addition, we have demonstrated the practical utility of the Pd_1_@TRIDAP catalyst in one-pot cross-electrophile coupling reactions to synthesize key pharmaceutical intermediates, such as Boscalid (fungicide **4t**) and OTBN (**4u**), a valuable precursor for antihypertensive “-sartan” drugs. These results highlight the versatility and broad applicability of our catalyst in enabling efficient and sustainable multistep synthetic processes.

## Materials and Methods

### Synthesis of the TRIDAP Framework

All reagents and solvents for the synthetic parts of the project were obtained from commercial suppliers and utilized without undergoing any additional purification steps; for more details, see the experimental part. The synthesis of polymeric TRIDAP coordinated organic material was conducted via a one-step polymerization process involving 2,6-diaminopyridine (2,6 DAP) and cyanuric chloride (CCl_3_). Initially, 2,6 DAP (10 mmol, 1 equiv) and Na_2_CO_3_ (2 equiv) were added to a three-necked round-bottom flask containing 200 mL of dioxane as a solvent. Subsequently, CCl_3_ (15 mmol, 1.5 equiv) was slowly added to the reaction mixture while maintaining a temperature range of 0–5 °C. The reaction mixture was then stirred for 2 h before being heated to 100 °C using an oil bath for 24 h. Upon completion of the reaction, a pale, yellow-colored slurry was collected via filtration. The collected material was washed several times using Milli-Q water, ethanol, and acetone. Finally, the resulting material was dried at 70 °C to yield the product (90% yield).

### Synthesis of Pd_1_@TRIDAP

The Pd_1_@TRIDAP catalyst was synthesized via impregnation, wherein 300 mg of TRIDAP organic material was first dispersed in 100 mL of Milli-Q water and the mixture sonicated for 15–20 min, followed by the slow addition of a solution of K_2_PdCl_4_ (3 wt %, 50 mL) during sonication and subsequent sonication for an additional 20 min. The mixture was then heated at 90 °C for 24 h. The resulting material was filtered, washed with Milli-Q water, and dried overnight at 75 °C. The dried material was redispersed in 50 mL of Milli-Q water, followed by slow addition of aqueous NaBH_4_ as a reducing agent, with stirring for 1 h. The final product, the Pd_1_@TRIDAP catalyst, was collected via filtration and washed with water, ethanol, and acetone successively to remove impurities, resulting in a catalyst ready for various catalytic applications.

### Catalyst Characterization

The chemical nature and bonding environment of the samples were assessed through FTIR spectroscopy with a Smart iTX accessory for ATR measurements mounted on a Thermo Scientific Nicolet iS20 FTIR Spectrometer equipped with a DTGS detector. 128 scans were acquired at a 4 cm^–1^ resolution, and then averaged to get the final IR spectrum in the 4000–400 cm^–1^ wavenumber range. The elemental H, N, and C content of all the samples was determined by combustion analysis using a Vario MICRO Elemental Analyzer, following the combustion of their combustion at high temperatures (>1000 °C) in an oxygen-rich environment. The Pd content in the samples was determined using ICP–OES on a PerkinElmer Optima 8300 spectrometer, following the microwave digestion of a weighed catalyst sample (10 mg) in nitric acid. Nitrogen physisorption measurements were collected on a 3P Sync 400 instrument at −196 °C. An outgassing step was carried out at 150 °C for 24 h before the measurements to remove any residual moisture or adsorbed contaminant from the catalytic surfaces. The specific surface area was determined through the BET method using the adsorption branch in the *p*/*p*_0_ range from 0.05 to 0.30. The porosity and pore distribution were calculated by using the model of quenched solid DFT for N_2_ adsorbed on porous carbon-type materials at 77 K. Powder XRD patterns were collected on a Bruker D2 Phaser diffractometer equipped with a Cu Kα radiation source (λ = 1.54 Å), and each XRD diffractogram was acquired by using a 2θ step size of 0.016° and a counting time of 0.4 s per step. TGA was carried out with a PerkinElmer STA 6000 analyzer. For each analysis, samples were heated from 30 to 750 °C at a heating rate of 10 °C/min in air atmosphere. The ex situ Raman spectrum of TRIDAP in powder form was recorded with the 1064 nm excitation line of the Nd:YVO_4_ laser integrated into a NEXUS FT-Raman instrument produced by THERMO-Nicolet. The spectrum was acquired using the micro stage with 256 scans and 2 W power. With this setup, the laser spot size is about 50 μm. For Pd_1_@TRIDAP, due to strong background observed with 1064 nm excitation, we adopted a different excitation line (633 nm, He–Ne laser), and the Raman spectrum was collected with the LABRAM-HR800 dispersive micro-Raman equipment produced by HORIBA Jobin Yvon. The powder of the sample was analyzed under the microscope (Olympus BX41) under a 50× objective (NA = 0.75) that provides a laser spot size of about 1 μm. The spectrum was acquired by averaging over 5 acquisitions (50 s each) with a laser power at the sample of 0.1 mW. The simulation of the off-resonance Raman spectra of TRIDAP and Pd_1_@TRIDAP was carried out by DFT methods using B3LYP and the 6-31G­(d,p) basis set for all elements except Pd. For describing Pd, we adopted the ECP28MDF pseudopotential and the associated VDZ basis set.^69^ The full geometry optimization of the models and the calculation of the off-resonance Raman intensities were carried out with the Gaussian software.^70^ The models were built by considering a small portion of the structures obtained by the periodic boundary calculations described below. The choice of the B3LYP functional was dictated by its well-recognized good performance in reproducing the Raman spectra of π-conjugated aromatic systems.^71^ Solid-state NMR measurements on the TRIDAP sample were conducted using an NMR Avance 500 instrument (Bruker BioSpin Srl). All cross-polarization magic-angle-spinning spectra for ^1^H, ^13^C, and ^15^N were acquired at 298 K with a spin rate of 12 kHz. For ^13^C and ^15^N measurements, a contact time of 8 ms was used to transfer polarization from protons to carbon or nitrogen atoms, with a recycling delay of 5s. Chemical shifts are expressed in parts per million (ppm). The sample, after being finely powdered, was placed in a zirconia rotor (4 mm in diameter and 18 mm in height), which was then inserted into the NMR instrument for measurements. For XPS measurements, the catalyst powder was gently distributed and pressed onto a double-sided carbon adhesive tape, which was fixed on a flag-style sample holder and then introduced into the ultrahigh-vacuum analysis chamber via a load-lock. Care was taken to fully cover the carbon tape to ensure that no additional signal contributions from the carbon tape are detected. The analysis chamber is equipped with a dual anode X-ray source (XR50, Specs) and a hemispherical electron energy analyzer (Argus CU, Scienta Omicron). The XP spectra of the catalyst samples were acquired with Al Kα radiation (1486.7 eV) at normal emission. For the Pd 3d, C 1s, and N 1s detail spectra, the analyzer pass energy was set to 50 eV. Spectral fitting of the Pd 3d spectra was performed with the XPS Peak software. Charging effects were considered by relating the BE scale to the C 1s signal of the aromatic carbon at 284.7 eV. SEM was performed by using a variable-pressure instrument (SEM Cambridge Stereoscan 360) at 100/120 Pa with a VPSE detector. The operating voltage was 20 kV with an electron beam current intensity of 150 pA. The focal distance was 8 mm. The specimens were used without any treatment. HAADF-STEM was performed using a Titan G2 60-300 (FEI) microscope operating at an accelerating voltage of 300 kV and with a high resolution (136 pm). Energy dispersive spectroscopy (EDS) was conducted in STEM mode with a Super-X system equipped with four silicon drift detectors (Bruker). STEM images were obtained by using a HAADF detector 3000 (Fischione). The samples were dispersed in ethanol, sonicated for 5 min, dropped on a copper grid with Lacey carbon film, and dried under air at room temperature before microscopy analysis. Ex situ XAS measurements were conducted at the 10C Wide XAFS beamline (BL10C) of Pohang Light Source-II (PLS-II). The incident monochromatic X-ray beam was shaped using a Si(111) double crystal monochromator, which reduced harmonic interference by detuning the beamline optics, thereby lowering the incident beam intensity by 40%. Slits with an opening of 1 mm (vertical) × 5 mm (horizontal) were used in all measurements. The measurements were performed at the Pd K-edge in transmission mode, utilizing ionization chamber detectors.

### General Synthetic Method for Miyaura Borylation Reaction

A sealed reaction vial equipped with a magnetic stir bar was charged with the aryl halides (0.5 mmol), B_2_pin_2_ (0.6 mmol), and base (2 mmol). Pd_1_@TRIDAP catalyst (10 mg) and 5 mL of anhydrous solvent were added. The vial was sealed and placed in an oil bath preheated to 80–90 °C, where the reaction mixture was stirred magnetically. For comparative studies, 46.4 mg of 1 wt % Pd@C_3_N_4_ or 4.64 mg of 10 wt % Pd/C (Sigma-Aldrich) was used as catalysts under identical reaction conditions. Progress was monitored by thin-layer chromatography (TLC) or gas chromatography–mass spectrometry (GC–MS). Upon completion, the reaction mixture was cooled to room temperature, transferred to a separatory funnel, and extracted with ethyl acetate (3 × 10 mL). The combined organic layers were washed with water (10 mL) and brine (10 mL), dried over anhydrous magnesium sulfate (MgSO_4_), filtered, and concentrated under reduced pressure by using a rotary evaporator. The crude product was purified by column chromatography using a suitable eluent (e.g., a mixture of hexane and ethyl acetate) to afford the desired boronate ester.

### Experimental Procedure for the Homo- and Hetero-Coupling of Aryl Halides

A sealed reaction vial equipped with a magnetic stir bar was charged with the aryl halide (0.5 mmol), B_2_pin_2_ (0.25 mmol), and sodium methoxide (NaOMe, 1.5 mmol). Pd_1_@TRIDAP catalyst (10 mg) and 5 mL of methanol (MeOH) were added. The vial was sealed and placed in an oil bath preheated to 80–90 °C, where the reaction mixture was stirred magnetically for 4 h. Progress was monitored by TLC or GC–MS. Upon completion, the reaction mixture was cooled to room temperature, transferred to a separatory funnel, and extracted with ethyl acetate (3 × 10 mL). The combined organic layers were washed with water (10 mL) and brine (10 mL), dried over anhydrous MgSO_4_, filtered, and concentrated under reduced pressure using a rotary evaporator.

To assess the sustainability of the process, we calculated the solvent consumption, CO_2_ generation, electricity consumption, and yield associated with the borylation and self-cascade reactions. Solvent consumption was quantified by measuring the volume of solvent utilized during the reaction and normalizing it to the number of moles of substrate employed. CO_2_ generation was analyzed through a life cycle assessment (LCA), which allowed us to evaluate the total carbon footprint of the process by considering both direct emissions and those associated with upstream activities, such as solvent production and energy consumption. Similarly, electricity consumption was also assessed via LCA, wherein we monitored the energy usage of the reaction setup throughout the experiments and evaluated the environmental impact of the electricity sources utilized. The yield of the final products was determined by using HPLC by comparing the peak areas of the desired products to those of the starting materials. All calculated values were normalized to the theoretical outputs of a two-step process, which was expected to yield higher values due to the additional transformation steps. This normalization facilitated a clearer assessment of the efficiency and environmental sustainability of the self-cascade synthesis methodology.

### Computational Details

Spin polarized DFT calculations were performed with the VASP code,^72–74^ using the generalized gradient approximation implemented in the PBE functional.^75^ The following valence electrons were treated explicitly: H (1s), B (2s, 2p), C (2s, 2p), N (2s, 2p), O (2s, 2p), and Pd (5s, 4d). They have been expanded on a set of plane waves with a kinetic energy cutoff of 400 eV, whereas the core electrons were treated with the projector augmented wave approach.^76,77^ The threshold criteria for electronic and ionic loops were set to 10^–5^ eV and 10^–2^ eV/Å, respectively. The sampling of the reciprocal space was restricted to the gamma point because of the cell dimensions. Dispersion forces have been included according to the Grimme’s D3 parametrization.^78^ Single point PBE0 ^79,80^ calculations have been performed to refine the electronic structure.^81^ This is typically a reasonable choice to provide accurate results without the need to perform computationally demanding geometry optimizations with hybrid functionals. Reaction free energy profiles were evaluated by adopting the ab initio thermodynamic approach,^82^ by adding to the DFT energy the contribution of zero-point energy correction and entropy terms. The first were calculated in a harmonic fashion. Entropies of reactants and products were determined through the formalism of the partition function. The vibrational entropy of solid-state species was determined as well. The BE of the Pd atom (*E*_b_) to the support (TRIDAP) was calculated as *E*_b_ = *E*_Pd1–TRIDAP_ – *E*_Pd1_ – *E*_TRIDAP_.

## Supplementary Material



## Notes

Additional physicochemical characterization of CHN, BET, XAS, NMR, SEM, STEM results, DFT models, catalytic tests for borylation and C–C coupling, mechanistic analysis, and full NMR data of all products (PDF)

## References

[1] Liu L., Corma A. (2023). Bimetallic Sites for Catalysis: From Binuclear Metal Sites to Bimetallic
Nanoclusters and Nanoparticles. Chem. Rev..

[2] Wu S.-M., Schmuki P. (2025). Single Atom Cocatalysts
in Photocatalysis. Adv. Mater..

[3] Saptal V. B., Ruta V., Bajada M. A., Vilé G. (2023). Single-Atom
Catalysis in Organic Synthesis. Angew. Chem.,
Int. Ed..

[4] Liang X., Fu N., Yao S., Li Z., Li Y. (2022). The Progress and Outlook
of Metal Single-Atom-Site Catalysis. J. Am.
Chem. Soc..

[5] Ji S., Chen Y., Wang X., Zhang Z., Wang D., Li Y. (2020). Chemical Synthesis
of Single Atomic Site Catalysts. Chem. Rev..

[6] Samantaray M. K., D’Elia V., Pump E., Falivene L., Harb M., Ould Chikh S., Cavallo L., Basset J.-M. (2020). The Comparison between
Single Atom Catalysis and Surface Organometallic Catalysis. Chem. Rev..

[7] Bajada M. A., Sanjosé-Orduna J., Di Liberto G., Tosoni S., Pacchioni G., Noël T., Vilé G. (2022). Interfacing Single-Atom Catalysis with Continuous-Flow
Organic Electrosynthesis. Chem. Soc. Rev..

[8] Samantaray M. K., Pump E., Bendjeriou-Sedjerari A., D’Elia V., Pelletier J. D. A., Guidotti M., Psaro R., Basset J.-M. (2018). Surface
Organometallic Chemistry in Heterogeneous Catalysis. Chem. Soc. Rev..

[9] Lang R., Du X., Huang Y., Jiang X., Zhang Q., Guo Y., Liu K., Qiao B., Wang A., Zhang T. (2020). Single-Atom Catalysts
Based on the Metal–Oxide Interaction. Chem. Rev..

[10] Jia H., Liao Q., Liu W., Cipriano L. A., Jiang H., Dixneuf P. H., Vilé G., Zhang M. (2024). Reductive Coupling
of N-Heteroarenes and 1,2-Dicarbonyls for Direct Access to γ-Amino
Acids, Esters, and Ketones Using a Heterogeneous Single-Atom Iridium
Catalyst. J. Am. Chem. Soc..

[11] Gawande M. B., Fornasiero P., Zbořil R. (2020). Carbon-Based
Single-Atom Catalysts
for Advanced Applications. ACS Catal..

[12] Qin R., Liu K., Wu Q., Zheng N. (2020). Surface Coordination Chemistry of
Atomically Dispersed Metal Catalysts. Chem.
Rev..

[13] Copéret C. (2019). Single-Sites
and Nanoparticles at Tailored Interfaces Prepared via Surface Organometallic
Chemistry from Thermolytic Molecular Precursors. Acc. Chem. Res..

[14] P S., John J., Rajan T. P. D., Anilkumar G. M., Yamaguchi T., Pillai S. C., Hareesh U. S. (2023). Graphitic Carbon
Nitride (g-C3N4) Based Heterogeneous Single Atom Catalysts: Synthesis,
Characterisation and Catalytic Applications. J. Mater. Chem. A.

[15] Pelletier J. D. A., Basset J.-M. (2016). Catalysis by Design: Well-Defined Single-Site Heterogeneous
Catalysts. Acc. Chem. Res..

[16] Kumar P., Singh G., Guan X., Lee J., Bahadur R., Ramadass K., Kumar P., Kibria Md. G., Vidyasagar D., Yi J., Vinu A. (2023). Multifunctional Carbon
Nitride Nanoarchitectures for
Catalysis. Chem. Soc. Rev..

[17] Rocha G. F. S. R., da Silva M. A. R., Rogolino A., Diab G. A. A., Noleto L. F. G., Antonietti M., Teixeira I. F. (2023). Carbon Nitride Based
Materials: More than Just a Support for Single-Atom Catalysis. Chem. Soc. Rev..

[18] Peralta R. A., Huxley M. T., Evans J. D., Fallon T., Cao H., He M., Zhao X. S., Agnoli S., Sumby C. J., Doonan C. J. (2020). Highly
Active Gas Phase Organometallic Catalysis Supported Within Metal–Organic
Framework Pores. J. Am. Chem. Soc..

[19] Witzke R. J., Chapovetsky A., Conley M. P., Kaphan D. M., Delferro M. (2020). Nontraditional
Catalyst Supports in Surface Organometallic Chemistry. ACS Catal..

[20] Iemhoff A., Vennewald M., Palkovits R. (2023). Single-Atom
Catalysts on Covalent
Triazine Frameworks: At the Crossroad between Homogeneous and Heterogeneous
Catalysis. Angew. Chem., Int. Ed..

[21] Sádaba I., López Granados M., Riisager A., Taarning E. (2015). Deactivation
of Solid Catalysts in Liquid Media: The Case of Leaching of Active
Sites in Biomass Conversion Reactions. Green
Chem..

[22] Shende V.
S., Saptal V. B., Bhanage B. M. (2019). Recent Advances Utilized in the Recycling
of Homogeneous Catalysis. Chem. Rec..

[23] Hammond C. (2017). Intensification
Studies of Heterogeneous Catalysts: Probing and Overcoming Catalyst
Deactivation during Liquid Phase Operation. Green Chem..

[24] Kramer S., Bennedsen N. R., Kegnæs S. (2018). Porous Organic Polymers Containing
Active Metal Centers as Catalysts for Synthetic Organic Chemistry. ACS Catal..

[25] Xu L.-H., Liu W., Liu K. (2023). Single Atom Environmental
Catalysis: Influence of Supports
and Coordination Environments. Adv. Funct. Mater..

[26] Li Z., Ji S., Liu Y., Cao X., Tian S., Chen Y., Niu Z., Li Y. (2020). Well-Defined Materials
for Heterogeneous Catalysis:
From Nanoparticles to Isolated Single-Atom Sites. Chem. Rev..

[27] Kwak M., Bok J., Lee B.-H., Kim J., Seo Y., Kim S., Choi H., Ko W., Hooch Antink W., Lee C. W., Yim G. H., Seung H., Park C., Lee K.-S., Kim D.-H., Hyeon T., Yoo D. (2022). Ni Single
Atoms on Carbon Nitride for Visible-Light-Promoted Full Heterogeneous
Dual Catalysis. Chem. Sci..

[28] Bhadra M., Sasmal H. S., Basu A., Midya S. P., Kandambeth S., Pachfule P., Balaraman E., Banerjee R. (2017). Predesigned Metal-Anchored
Building Block for In Situ Generation of Pd Nanoparticles in Porous
Covalent Organic Framework: Application in Heterogeneous Tandem Catalysis. ACS Appl. Mater. Interfaces.

[29] Ding S.-Y., Gao J., Wang Q., Zhang Y., Song W.-G., Su C.-Y., Wang W. (2011). Construction of Covalent
Organic Framework for Catalysis: Pd/COF-LZU1
in Suzuki–Miyaura Coupling Reaction. J. Am. Chem. Soc..

[30] Vijeta A., Casadevall C., Roy S., Reisner E. (2021). Visible-Light Promoted
C–O Bond Formation with an Integrated Carbon Nitride–Nickel
Heterogeneous Photocatalyst. Angew. Chem., Int.
Ed..

[31] Kim S., Bok J., Lee B.-H., Choi H., Seo Y., Kim J., Kim J., Ko W., Lee K.-S., Cho S.-P., Hyeon T., Yoo D. (2023). Orthogonal
Dual Photocatalysis of Single Atoms on Carbon Nitrides
for One-Pot Relay Organic Transformation. ACS
Nano.

[32] Jia J., Bu X., Yang X. (2022). A Cobalt Covalent Organic Framework: A Dual-Functional
Atomic-Level Catalyst for Visible-Light-Driven C–H Annulation
of Amides with Alkynes. J. Mater. Chem. A.

[33] Han W.-K., Liu Y., Feng J.-D., Yan X., Pang H., Gu Z.-G. (2023). Engineering
a Molecular Ruthenium Catalyst into Three-Dimensional Metal Covalent
Organic Frameworks for Efficient Water Oxidation. Chem. Sci..

[34] Vardhan H., Al-Enizi A. M., Nafady A., Pan Y., Yang Z., Gutiérrez H. R., Han X., Ma S. (2021). Single-Pore
versus
Dual-Pore Bipyridine-Based Covalent–Organic Frameworks: An
Insight into the Heterogeneous Cata-lytic Activity for Selective C-H
Functionalization. Small.

[35] Bajada M. A., Di Liberto G., Tosoni S., Ruta V., Mino L., Allasia N., Sivo A., Pacchioni G., Vilé G. (2023). Light-Driven C–O Coupling of Carboxylic Acids
and Alkyl Halides over a Ni Single-Atom Catalyst. Nat. Synth..

[36] Samudrala K. K., Conley M. P. (2023). A Supported Ziegler-Type Organohafnium Site Metabolizes
Polypropylene. J. Am. Chem. Soc..

[37] Xu H.-S., Luo Y., See P. Z., Li X., Chen Z., Zhou Y., Zhao X., Leng K., Park I.-H., Li R., Liu C., Chen F., Xi S., Sun J., Loh K. P. (2020). Divergent
Chemistry Paths for 3D and 1D Metallo-Covalent Organic Frameworks
(COFs). Angew. Chem., Int. Ed..

[38] Guan Q., Zhou L.-L., Dong Y.-B. (2022). Metalated
Covalent Organic Frameworks:
From Synthetic Strategies to Diverse Applications. Chem. Soc. Rev..

[39] Almansaf Z., Hu J., Zanca F., Shahsavari H. R., Kampmeyer B., Tsuji M., Maity K., Lomonte V., Ha Y., Mastrorilli P., Todisco S., Benamara M., Oktavian R., Mirjafari A., Moghadam P. Z., Khosropour A. R., Beyzavi H. (2021). Pt­(II)-Decorated Covalent Organic Framework for Photocatalytic
Difluoroalkylation and Oxidative Cyclization Reactions. ACS Appl. Mater. Interfaces.

[40] Lin S., Diercks C. S., Zhang Y.-B., Kornienko N., Nichols E. M., Zhao Y., Paris A. R., Kim D., Yang P., Yaghi O. M., Chang C. J. (2015). Covalent Organic
Frameworks Comprising Cobalt Porphyrins for Catalytic CO2 Reduction
in Water. Science.

[41] Zhou W., Deng W.-Q., Lu X. (2024). Metallosalen Covalent
Organic Frameworks
for Heterogeneous Catalysis. Interdiscip. Mater..

[42] Li L.-H., Feng X.-L., Cui X.-H., Ma Y.-X., Ding S.-Y., Wang W. (2017). Salen-Based Covalent
Organic Framework. J.
Am. Chem. Soc..

[43] Zhao X., Pachfule P., Thomas A. (2021). Covalent Organic Frameworks (COFs)
for Electrochemical Applications. Chem. Soc.
Rev..

[44] Lang R., Xi W., Liu J.-C., Cui Y.-T., Li T., Lee A. F., Chen F., Chen Y., Li L., Li L., Lin J., Miao S., Liu X., Wang A.-Q., Wang X., Luo J., Qiao B., Li J., Zhang T. (2019). Non-Defect-Stabilized
Thermally Stable Single-Atom Catalyst. Nat.
Commun..

[45] Zhao H., Liu X., Zeng C., Liu W., Tan L. (2024). Thermochemical CO_2_ Reduction to Methanol
over Metal-Based Single-Atom Catalysts
(SACs): Outlook and Challenges for Development. J. Am. Chem. Soc..

[46] Kaiser S. K., Chen Z., Faust Akl D., Mitchell S., Pérez-Ramírez J. (2020). Single-Atom
Catalysts across the Periodic Table. Chem. Rev..

[47] Kment S. ˇ., Bakandritsos A., Tantis I., Kmentová H., Zuo Y., Henrotte O., Naldoni A., Otyepka M., Varma R. S., Zbořil R. (2024). Single-Atom
Catalysts Based on Earth-Abundant Metals
for Energy-Related Applications. Chem. Rev..

[48] Abdel-Mageed A. M., Rungtaweevoranit B., Parlinska-Wojtan M., Pei X., Yaghi O. M., Behm R. J. (2019). Highly
Active and Stable Single-Atom Cu Catalysts Supported
by a Metal–Organic Framework. J. Am.
Chem. Soc..

[49] Pachfule P., Kandambeth S., Díaz Díaz D., Banerjee R. (2014). Highly Stable
Covalent Organic Framework–Au Nanoparticles Hybrids for Enhanced
Activity for Nitrophenol Reduction. Chem. Commun..

[50] Dong J., Han X., Liu Y., Li H., Cui Y. (2020). Metal–Covalent
Organic Frameworks (MCOFs): A Bridge Between Metal–Organic
Frameworks and Covalent Organic Frameworks. Angew. Chem., Int. Ed..

[51] Chen H., Liu W., Laemont A., Krishnaraj C., Feng X., Rohman F., Meledina M., Zhang Q., Van Deun R., Leus K., Van Der Voort P. (2021). A Visible-Light-Harvesting Covalent Organic Framework
Bearing Single Nickel Sites as a Highly Efficient Sulfur–Carbon
Cross-Coupling Dual Catalyst. Angew. Chem.,
Int. Ed..

[52] Liu G., Liu S., Lai C., Qin L., Zhang M., Li Y., Xu M., Ma D., Xu F., Liu S., Dai M., Chen Q. (2024). Strategies
for Enhancing the Photocatalytic and Electrocatalytic
Efficiency of Covalent Triazine Frameworks for CO2 Reduction. Small.

[53] Alsudairy Z., Brown N., Campbell A., Ambus A., Brown B., Smith-Petty K., Li X. (2023). Covalent Organic Frameworks in Heterogeneous
Catalysis: Recent Advances and Future Perspective. Mater. Chem. Front..

[54] Dong J., Han X., Liu Y., Li H., Cui Y. (2020). Metal–Covalent
Organic Frameworks (MCOFs): A Bridge Between Metal–Organic
Frameworks and Covalent Organic Frameworks. Angew. Chem., Int. Ed..

[55] Jati A., Dey K., Nurhuda M., Addicoat M. A., Banerjee R., Maji B. (2022). Dual Metalation
in a Two-Dimensional Covalent Organic Framework for Photocatalytic
C–N Cross-Coupling Reactions. J. Am.
Chem. Soc..

[56] Yan R., Mishra B., Traxler M., Roeser J., Chaoui N., Kumbhakar B., Schmidt J., Li S., Thomas A., Pachfule P. (2023). A Thiazole-Linked
Covalent Organic Framework for Lithium-Sulfur
Batteries. Angew. Chem., Int. Ed..

[57] Roy M., Mishra B., Maji S., Sinha A., Dutta S., Mondal S., Banerjee A., Pachfule P., Adhikari D. (2024). Covalent Organic
Framework Catalyzed Amide Synthesis Directly from Alcohol Under Red
Light Excitation. Angew. Chem., Int. Ed..

[58] Basak A., Karmakar A., Dutta S., Roy D., Paul S., Nishiyama Y., Pathak B., Kundu S., Banerjee R. (2025). Metal-Free
Electrocatalytic Alkaline Water Splitting by Porous Macrocyclic Proton
Sponges. Angew. Chem., Int. Ed..

[59] Park J. H., Lee C. H., Ju J.-M., Lee J.-H., Seol J., Lee S. U., Kim J.-H. (2021). Bifunctional Covalent
Organic Framework-Derived
Electrocatalysts with Modulated p-Band Centers for Rechargeable Zn–Air
Batteries. Adv. Funct. Mater..

[60] Li Z., Ren Q., Wang X., Chen W., Leng L., Zhang M., Horton J. H., Liu B., Xu Q., Wu W., Wang J. (2021). Highly Active and Stable Palladium Single-Atom Catalyst Achieved
by a Thermal Atomization Strategy on an SBA-15 Molecular Sieve for
Semi-Hydrogenation Reactions. ACS Appl. Mater.
Interfaces.

[61] Zhang R., Liu Z., Zheng S., Wang L., Zhang L., Qiao Z.-A. (2023). Pyridinic
Nitrogen Sites Dominated Coordinative Engineering of Subnanometric
Pd Clusters for Efficient Alkynes’ Semihydrogenation. Adv. Mater..

[62] Mou K., Meng F., Zhang Z., Li X., Li M., Jiao Y., Wang Z., Bai X., Zhang F. (2024). Pyridazine-Promoted
Construction of Vinylene-Linked Covalent Organic Frameworks with Exceptional
Capability of Stepwise Water Harvesting. Angew.
Chem., Int. Ed..

[63] Socrates, G. Infrared and Raman Characteristic Group Frequencies: Tables and Charts, 3rd Edition, Wiley, 2004.

[64] Edwards, H. G. M. Spectra-Structure Correlations in Raman Spectroscopy. In Handbook of Vibrational Spectroscopy; Chalmers, J. M. ; Griffiths, P. R. , Eds.; Wiley, 2006.

[65] Luo Q., Wang H., Xiang Q., Lv Y., Yang J., Song L., Cao X., Wang L., Xiao F.-S. (2024). Polymer-Supported
Pd Nanoparticles for Solvent-Free Hydrogenation. J. Am. Chem. Soc..

[66] Ouyang Z., Sheng G., Zhong Y., Wang J., Cai J., Deng S., Deng Q. (2025). Palladium Single Atom-Supported Covalent
Organic Frameworks for Aqueous-Phase Hydrogenative Hydrogenolysis
of Aromatic Aldehydes via Hydrogen Heterolysis. Angew. Chem., Int. Ed..

[67] Di
Liberto G., Giordano L., Pacchioni G. (2024). Predicting
the Stability of Single-Atom Catalysts in Electrochemical Reactions. ACS Catal..

[68] Peterson K. A., Figgen D., Dolg M., Stoll H. (2007). Energy-Consistent Relativistic
Pseudopotentials and Correlation Consistent Basis Sets for the 4d
Elements Y–Pd. J. Chem. Phys..

[69] Frisch, M. J. ; Trucks, G. W. ; Schlegel, H. B. ; Scuseria, G. E. ; Robb, M. A. ; Cheeseman, J. R. ; Scalmani, G. ; Barone, V. ; Mennucci, B. ; Petersson, G. A. ; Nakatsuji, H. ; Caricato, M. ; Li, X. ; Hratchian, H. P. ; Izmaylov, A. F. ; Bloino, J. ; Zheng, G. ; Sonnenberg, J. L. ; Hada, M. ; Ehara, M. ; Toyota, K. ; Fukuda, R. ; Hasegawa, J. ; Ishida, M. ; Nakajima, T. ; Honda, Y. ; Kitao, O. ; Nakai, H. ; Vreven, T. ; Montgomery, J. A. ; Peralta, J. E. ; Ogliaro, F. ; Bearpark, M. ; Heyd, J. J. ; Brothers, E. ; Kudin, K. N. ; Staroverov, V. N. ; Kobayashi, R. ; Normand, J. ; Raghavachari, K. ; Rendell, A. ; Burant, J. C. ; Iyengar, S. S. ; Tomasi, J. ; Cossi, M. ; Rega, N. ; Millam, J. M. ; Klene, M. ; Knox, J. E. ; Cross, J. B. ; Bakken, V. ; Adamo, C. ; Jaramillo, J. ; Gomperts, R. ; Stratmann, R. E. ; Yazyev, O. ; Austin, A. J. ; Cammi, R. ; Pomelli, C. ; Ochterski, J. W. ; Martin, R. L. ; Morokuma, K. ; Zakrzewski, V. G. ; Voth, G. A. ; Salvador, P. ; Dannenberg, J. J. ; Dapprich, S. ; Daniels, A. D. ; Farkas, O. ; Foresman, J. B. ; Ortiz, J. V. ; Cioslowski, J. ; Fox, D. J. Gaussian 09, Revision E.01.

[70] Zanchi C., Lucotti A., Pistaffa M., Ossi P. M., Trusso S., Fontana F., Carminati G., Rizzo S., Tommasini M. (2020). A Raman and
SERS Study on the Interactions of Aza[5]­Helicene and Aza[6]­Helicene
with a Nanostructured Gold Surface. Vib. Spectrosc..

[71] Kresse G., Hafner J. (1993). Ab Initio Molecular Dynamics for
Liquid Metals. Phys. Rev. B:Condens. Matter
Mater. Phys..

[72] Kresse G., Furthmüller J. (1996). Efficiency
of Ab-Initio Total Energy Calculations for
Metals and Semiconductors Using a Plane-Wave Basis Set. Comput. Mater. Sci..

[73] Kresse G., Hafner J. (1994). Ab Initio Molecular-Dynamics Simulation
of the Liquid-Metal–Amorphous-Semiconductor
Transition in Germanium. Phys. Rev. B:Condens.
Matter Mater. Phys..

[74] Perdew J. P., Burke K., Ernzerhof M. (1996). Generalized
Gradient Approximation
Made Simple. Phys. Rev. Lett..

[75] Blöchl P. E. (1994). Projector
Augmented-Wave Method. Phys. Rev. B:Condens.
Matter Mater. Phys..

[76] Kresse G., Joubert D. (1999). From Ultrasoft Pseudopotentials to
the Projector Augmented-Wave
Method. Phys. Rev. B:Condens. Matter Mater.
Phys..

[77] Grimme S., Antony J., Ehrlich S., Krieg H. (2010). A Consistent and Accurate
Ab Initio Parametrization of Density Functional Dispersion Correction
(DFT-D) for the 94 Elements H-Pu. J. Chem. Phys..

[78] Adamo C., Barone V. (1999). Toward Reliable Density Functional Methods without
Adjustable Parameters: The PBE0Model. J. Chem.
Phys..

[79] Perdew J.
P., Ernzerhof M., Burke K. (1996). Rationale for Mixing Exact Exchange
with Density Functional Approximations. J. Chem.
Phys..

[80] Barlocco I., Cipriano L. A., Di Liberto G., Pacchioni G. (2023). Modeling Hydrogen
and Oxygen Evolution Reactions on Single Atom Catalysts with Density
Functional Theory: Role of the Functional. Adv.
Theory Simul..

[81] Nørskov J. K., Bligaard T., Logadottir A., Kitchin J. R., Chen J. G., Pandelov S., Stimming U. (2005). Trends in
the Exchange Current for
Hydrogen Evolution. J. Electrochem. Soc..

[82] Nørskov J. K., Bligaard T., Rossmeisl J., Christensen C. H. (2009). Towards
the Computational Design of Solid Catalysts. Nat. Chem..

