# Supplementary material for: An Adaptive Palladium Single-Atom Catalyst Enabling Reactivity Switching between Borylation and C–C Coupling
Source: J Am Chem Soc. 2025 May 23;147(22):18524–40. doi: 10.1021/jacs.4c17943 (PMC12147128; doi:10.1021/jacs.4c17943)
Supplement: Supplementary file 1 [file ja4c17943_si_001.pdf]

*Supporting Information*

# An Adaptive Palladium Single-Atom Catalyst Enabling Reactivity Switching Between Borylation and C–C Coupling


*Vitthal B. Saptal,[a] Clara Saetta,[b] Adriana Laufenböck,[c] Martin Sterrer,[c] Ik Seon Kwon,[d] Andrea Lucotti,[a] Matteo Tommasini,[a] Ondřej Tomanec,[e] Aristides Bakandritsos,[e,f] Giovanni Di Liberto,*[,b] Gianfranco Pacchioni,[b] and Gianvito Vilé*[,a]*

[a] *Department of Chemistry, Materials, and Chemical Engineering "Giulio Natta", Politecnico di Milano, Piazza Leonardo da Vinci 32, 20133 Milano, Italy.*

[b] *Department of Materials Science, University of Milan Bicocca, Via Roberto Cozzi 55, 20125 Milano, Italy.*

[c] *Institute of Physics, University of Graz, Universitätsplatz 5, 8010 Graz, Austria.*

[d] *Department of Energy Science and Engineering, Kunsan National University, 558 Daehak-ro, 54150 Gunsan-si, Republic of Korea.*

[e] *Regional Centre of Advanced Technologies and Materials, Czech Advanced Technology and Research Institute (CATRIN), Palacký University Olomouc, Šlechtitelů 241/27, 783 71 Olomouc-Holice, Czech Republic.*

[f] *Nanotechnology Centre, Centre of Energy and Environmental Technologies, VŠB–Technical University of Ostrava, 17. listopadu 2172/15, 708 00 Ostrava-Poruba, Czech Republic*

* *Corresponding authors. E-mails: giovanni.diliberto@unimib.it (G.D.L.) and gianvito.vile@polimi.it (G.V.).*

## Contents

1. Materials and reagents page S3
2. Materials characterization page S4
3. Computational structural analysis page S9
4. Supplementary catalytic tests page S13
5. Reaction mechanisms page S18
6. NMR spectral data of products page S22
7. NMR spectra of products page S30
8. References for the Supporting Information page S57

## 1. Materials and reagents

All aryl halides, diborane, and other reagents were purchased from commercial suppliers including Sigma-Aldrich, BLD Pharma, Merck, and TCI Chemicals, and used without further purification. GC-MS analyses were performed using an Agilent 5977C GC/MSD™ equipped with an HP-5MS Ultra Inert fused silica GC column (30 m, 0.25 mm, 0.25 μm) coupled with a mass spectrometer. Pd@$C_3N_4$ synthesized using our previous reported method.[8] The GC-MS method involved setting the initial oven temperature at 40°C for 2 min, and then increasing the temperature to 80°C using a heating ramp of 20°C/min and holding the aliquot at this temperature for 2 min, followed by a final increase to 230°C using 25°C/min. Integrated areas were converted to mole percentages using a previously constructed calibration curve, and this data was used to calculate yields. $^{1}$H NMR and $^{13}$C NMR spectra were recorded on a Bruker 400 MHz spectrometer, with yields calculated using an internal standard.

## 2. Materials characterization

**Table S1.** Compositional properties of the materials.

| Catalyst | C[a] (wt.%) | N[a] (wt.%) | H[a] (wt.%) |
|---|---|---|---|
| TRIDAP | 46.9 ± 0.2 | 36.3 ± 0.1 | 2.8 ± 0.2 |
| $Pd_1$@TRIDAP | 44.7 ± 0.2 | 35.9 ± 0.1 | 1.6 ± 0.2 |

[a]The carbon (C), nitrogen (N), and hydrogen (H) contents (wt.%) were obtained using CHN elemental analysis *via* combustion, where the sample is burned in an oxygen-rich environment, and the resulting gases ($CO_2$, $H_2O$, and $N_2$) are quantified.

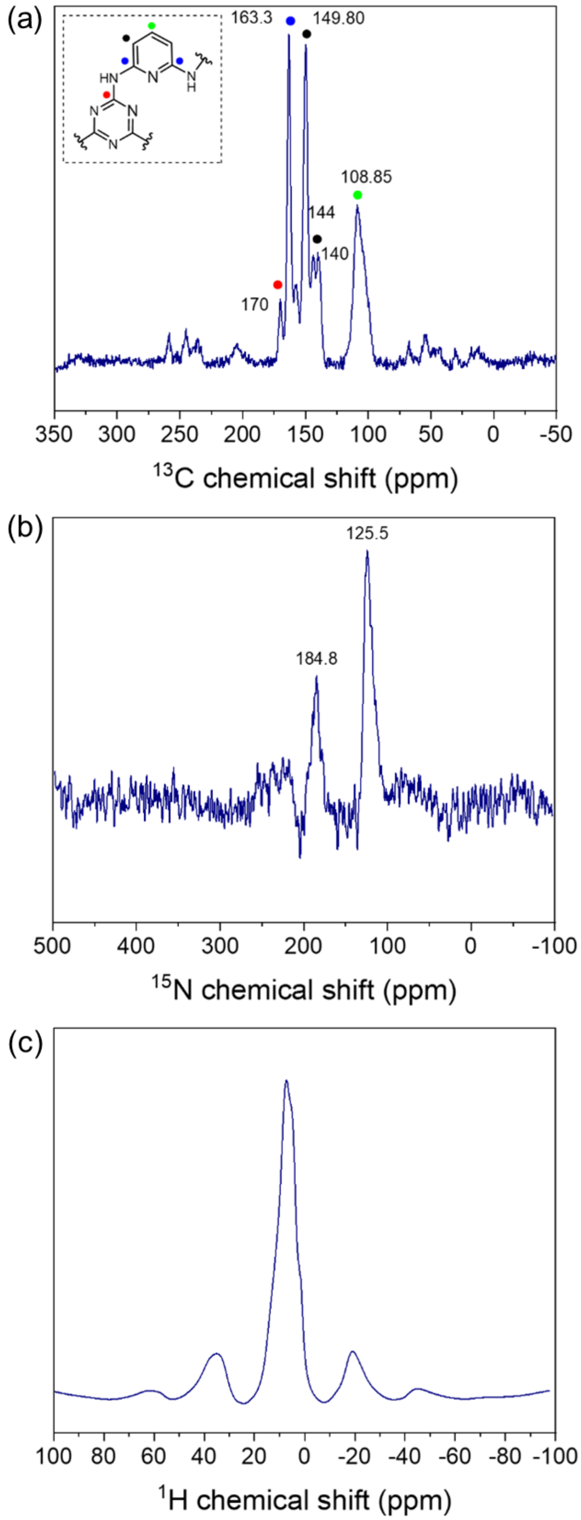


**Figure S1.** Solid-state NMR spectra of TRIDAP, showing the chemical environments of $^{13}C$ (a), $^{15}N$ (b), and $^{1}H$ (c) nuclei.

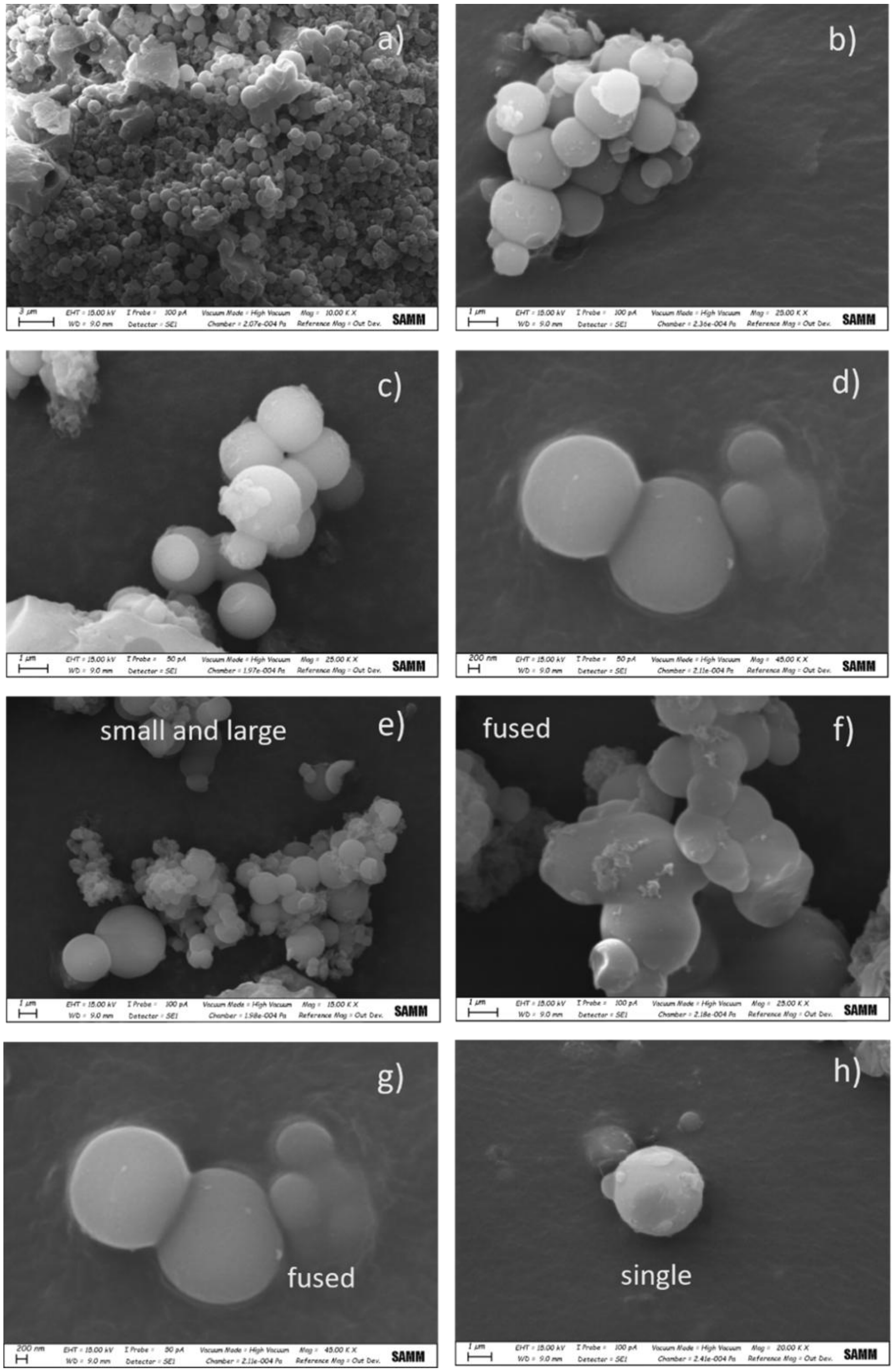


**Figure S2.** SEM analysis of TRIDAP, showing consistent spherical morphology in different micrographs.

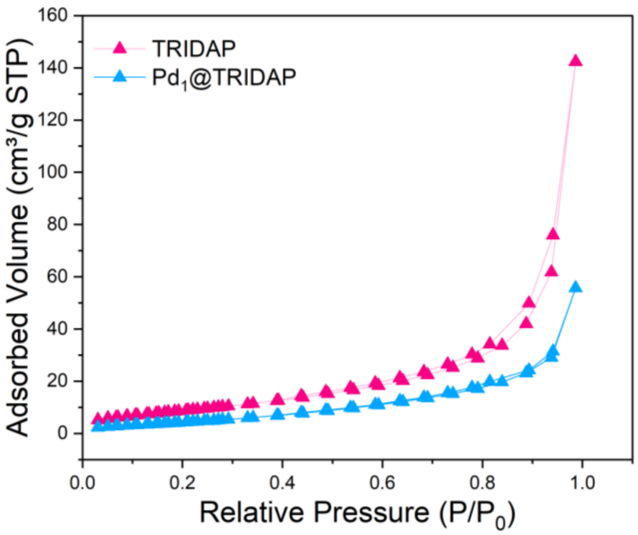


**Figure S3.** $N_2$ adsorption-desorption isotherms of TRIDAP (pink triangles) and $Pd_1$@TRIDAP (blue triangles).

**Table S2**. BET and BJH textural parameters of TRIDAP and $Pd_1$@TRIDAP.

| **Parameters** | **TRIDAP** | **$Pd_1$@TRIDAP** |
|---|---|---|
| BET Surface Area[a] ($m^2$/g) | 34.158 | 18.078 |
| Total Pore Volume[b] at $p/p_0$ ($cm^3$/g) | 0.220 | 0.086 |
| Average Pore Diameter (4V/A) (nm) | 25.791 | 19.073 |
| BJH Adsorption Cumulative Surface Area ($m^2$/g) | 58.736 | 35.336 |
| BJH Adsorption Cumulative Pore Volume ($cm^3$/g) | 0.236 | 0.097 |
| BJH Adsorption Median Pore Diameter (nm) | 31.602 | 21.194 |

[a]The specific surface area ($S_{BET}$) was measured using the Brunauer-Emmett-Teller (BET) model, applied to nitrogen adsorption-desorption isotherms at liquid nitrogen temperatures (-196°C). [b]The pore volume ($V_{pore}$) and pore diameter ($d_{pore}$) were calculated using the Barrett-Joyner-Halenda (BJH) method, analyzing the desorption branch of the isotherm to estimate mesoporous structures.

**Table S3.** Fitted XAS values for the $Pd_1$@TRIDAP and reference materials.

| Sample | Scattering path | $N$[a] | $R$[b] (Å) | $\sigma^2$[c] ($10^{-3}$ Å$^2$) | $\Delta E$[d] (eV) | R-factor[e] |
|---|---|---|---|---|---|---|
| Pd foil | Pd-Pd | 12 | 2.745±0.007 | 5.59±0.89 | 3.99±1.62 | 0.001 |
| PdO powder | Pd-O | 4 | 2.017±0.004 | 2.30±0.34 | 3.23±1.04 | 0.008 |
| | Pd-Pd | 4 | 3.052±0.006 | 4.74±1.49 | | |
| | Pd-Pd | 8 | 3.442±0.006 | 3.33±1.78 | | |
| $Pd_1$@TRIDAP | Pd-N | 3.1±1.3 | 2.055±0.008 | 1.80±0.43 | 4.13±2.09 | 0.008 |
| | Pd-N | 1.0±0.3 | 2.599±0.030 | 2.22±0.37 | | |

[a]Coordination number, representing the number of atoms surrounding the absorbing atom within a specific distance. [b]Interatomic distance, representing the average bond length or interatomic distance between the absorbing atom and the neighboring atoms. [c]Debye-Waller factor, representing the mean square disorder in the interatomic distance, which accounts for thermal and static disorder in the system. A higher $\sigma^2$ indicates greater variation in bond lengths (due to temperature or structural disorder). [d]Energy shift, representing the difference between experimental edge energy and the theoretical value. [e]Fit quality, measuring of the goodness of fit between the experimental EXAFS data and the theoretical model. A lower R-factor indicates a better fit.

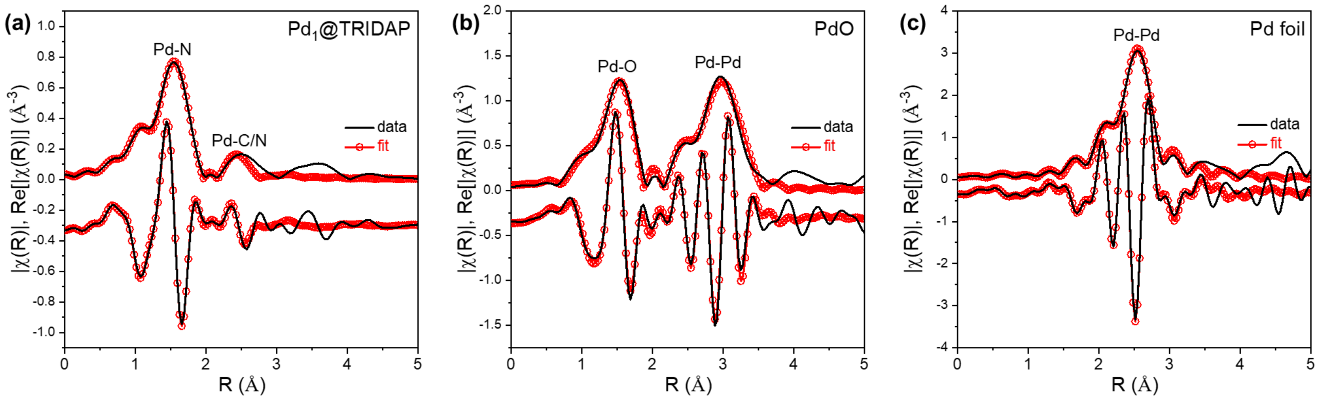


**Figure S4.** Phase-uncorrected FT-EXAFS spectra, k2-weighted Fourier-transformed EXAFS data (χ(R)) plotted as a function of radial distance (R), showing the first-shell EXAFS fitting performed with the corresponding DFT structures.

## 3. Computational structural analysis

The properties of a single-atom catalyst are highly influenced by the support and its local coordination environment. Drawing from experimental evidence regarding the quantities of C, N, H, and Cl species, we designed a covalent organic framework with a 2D periodic crystal structure. We investigated the reaction stoichiometry based on the monomers used. The triazine-like monomer contains three carbon and three nitrogen atoms, while the pyridine-like monomer consists of five carbon and three nitrogen atoms. Moreover, the chlorine and hydrogen contents vary depending on the degree of polymerization. This information allows us to establish a linear system to determine the triazine-to-pyridine ratio for constructing the polymer:

$$m_{\mathrm{C}} = (\mathrm{AW_C} \times 3)n_{\mathrm{T}} + (\mathrm{AW_C} \times 5)n_{\mathrm{P}}$$

$$m_{\mathrm{N}} = (\mathrm{AW_N} \times 3)n_{\mathrm{T}} + (\mathrm{AW_N} \times 5)n_{P}$$

$$m_{\mathrm{H}} = \mathrm{AW_H} x n_{\mathrm{P}}$$

$$m_{\mathrm{Cl}} = \mathrm{AW_N} y n_{\mathrm{T}}$$

where $m_{\mathrm{C}}$ is the carbon mass, $m_{\mathrm{N}}$ is the nitrogen mass, $m_{\mathrm{H}}$ is the hydrogen mass, $m_{\mathrm{Cl}}$ is the chlorine mass, all expressed in g·mol$^{-1}$. $\mathrm{AW_x}$ is the atomic weight of the atom $x$. $n_{\mathrm{T}}$ is the stoichiometric coefficient for the triazine-like monomer, $n_{\mathrm{P}}$ is the stochiometric coefficient for the pyridine-like monomer, $x$ and $y$ are respectively the average H and Cl content in the respective precursor (pyridine for hydrogen, and triazine for chlorine).

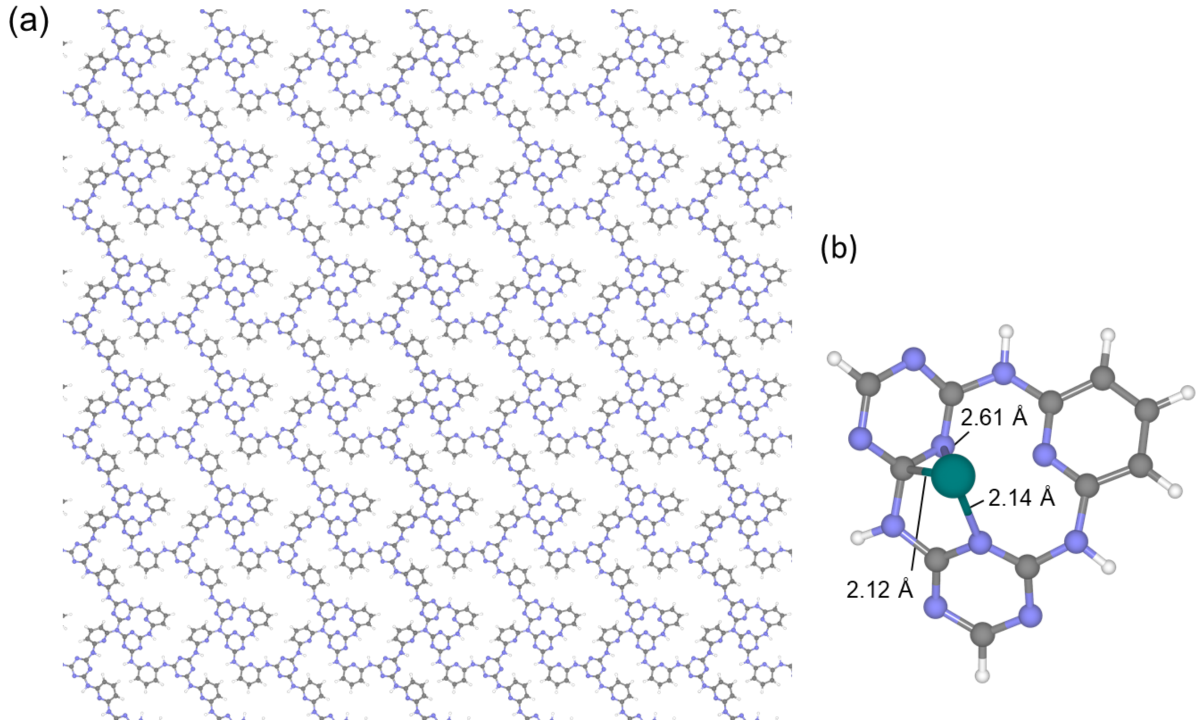


**Figure S5.** (a) Corrugated polymer surface structure containing 3N-cavities. (b) Pd anchored to the 3N-cavity of TRIDAP.

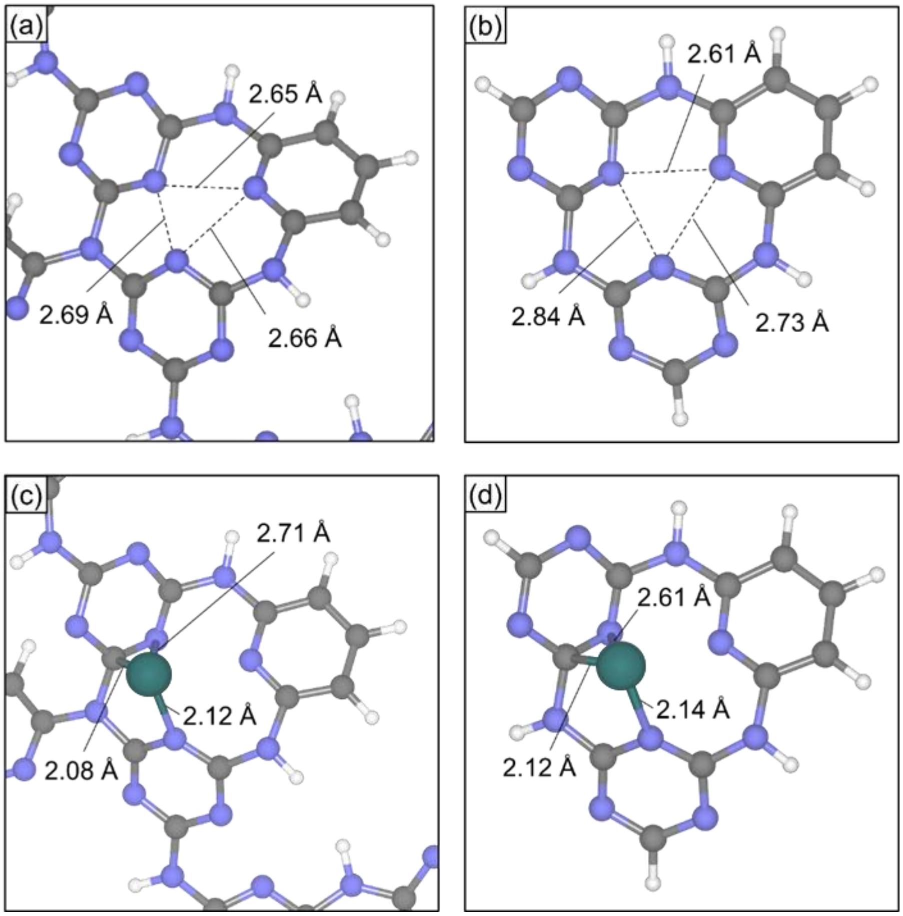


**Figure S6.** (a) Extended periodic model of 3N-TRIDAP. (b) Molecular analogue of 3N-TRIDAP. (c) Pd atom adsorbed on the support in the periodic model. (d) Pd atom adsorbed on the support in the molecular analogue.

We chose to model molecular analogues instead of periodic crystalline structures, ensuring the same cavity size and local atomic arrangement. This approach avoids introducing any spurious effects on the cavity structure or the binding energy of the metal atom. To validate this, we conducted test calculations on one of the models (3N-TRIDAP) using both periodic and molecular simulations. The cavity size remained consistent (see **Figures S6a-S6b**). Additionally, when the Pd atom was anchored at two different sites, the resulting structures were very similar (**Figures S6c-S6d**), with nearly identical adhesion energies and only a negligible difference of $\Delta E$ = 0.01 eV.

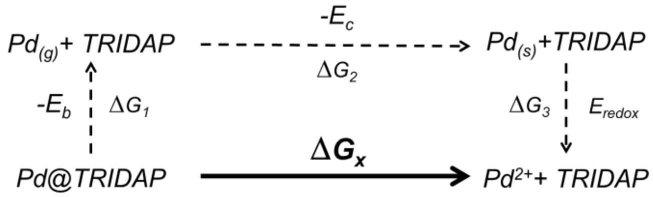


**Figure S7.** Thermodynamic cycle for predicting the stability of single-atom catalysts against metal dissolution. We assessed the stability of various catalysts using a thermodynamic cycle informed by previous work conducted by some of us.[1] The target free energy ($\Delta G$) can be expressed as the sum of three terms: $\Delta G = \Delta G_1 + \Delta G_2 + \Delta G_3$. The first step ($\Delta G_1$) corresponds to the binding energy of the metal atom to the support. The second term ($\Delta G_2$) represents the cohesive energy of the bulk metal, which can be obtained from the experimental International Tables. The third component ($\Delta G_3$) is derived from the experimental redox potential. For this analysis, we approximate these values using aqueous solutions; thus, the dissolution of ions in methanol is less favorable than in water. Consequently, the predicted stability is calculated under more challenging conditions than those encountered in actual experiments. Based on this thermodynamic cycle, only one quantity needs to be calculated, which serves as the descriptor for the stability of the SAC, as detailed in other studies.[1] Specifically, the energy of the first process ($\Delta G_1$) is the negative of the binding energy ($E_b$) of the single-atom catalyst. The binding energies are found to be -1.55 eV and -2.50 eV for $Pd_1$@3N-TRIDAP and $Pd_1$@4N-TRIDAP.

## 4. Supplementary catalytic tests and comparison of table

**Table S4.** Optimization of reaction conditions for Miyaura borylation.

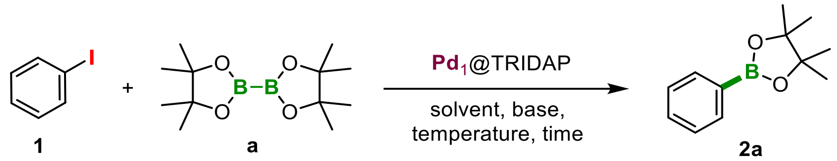


| Entry | Solvent | Base | Temperature (°C) | Time (h) | Yield (%) |
|---|---|---|---|---|---|
| 1[a] | Methanol | KOAc | 80 | 7 | 33 |
| 2[b] | Methanol | KOAc | 80 | 7 | 45 |

Reaction conditions: the reaction conditions were iodobenzene (1 mmol), $B_2Pin_2$ (1.3 mmol), KOAc (2 mmol), methanol (5 mL), $Pd_1$@TRIDAP (10 mg). [a] $PdCl_2$(2 mol%). [b] $Pd(OAc)_2$ (2 mol%).

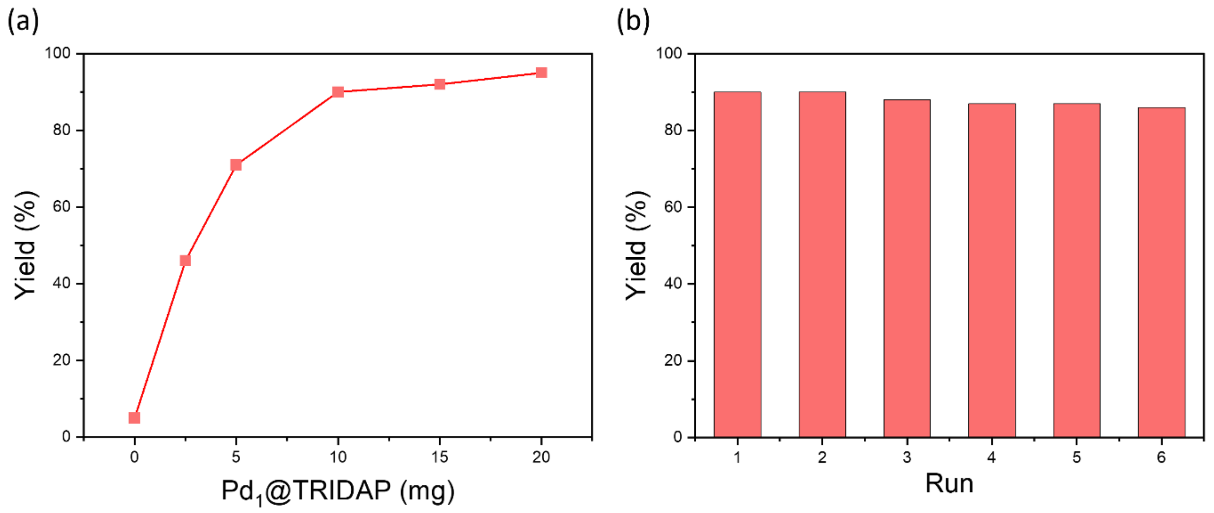


**Figure S8.** (a) effect of loading of $Pd_1$@TRIDAP and (b) recyclability study of $Pd_1$@TRIDAP in the Miyaura borylation reaction. Reaction conditions: iodobenzene (1 mmol), $B_2Pin_2$ (1.3 mmol), KOAc (1.5 mmol), methanol (5 mL), and $Pd_1$@TRIDAP (10 mg). All yields are isolated yields.

**Table S5.** Optimization of reaction conditions for cascade borylation-coupling reaction.

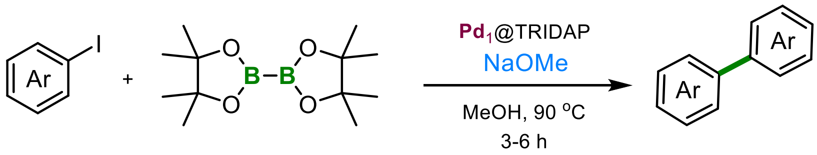


| Entry | Base | Solvent | Yield (%) |
|---|---|---|---|
| 1 | $K_2CO_3$ | MeOH | 28 |
| 2 | NaOEt | MeOH | 85 |
| 3 | $K_3PO_4$ | MeOH | 15 |
| 4 | KO*t*Bu | MeOH | 88 |
| 5 | NaOMe | EtOH | 80 |
| 6 | NaOMe | - | 52 |
| 7 | NaOMe | THF | 67 |

Reaction conditions: the reaction conditions were iodobenzene (1 mmol), $B_2Pin_2$ (0.5 mmol), base (1.5 mmol), methanol (5 mL), 90$^o$C, $Pd_1$@TRIDAP (10 mg).

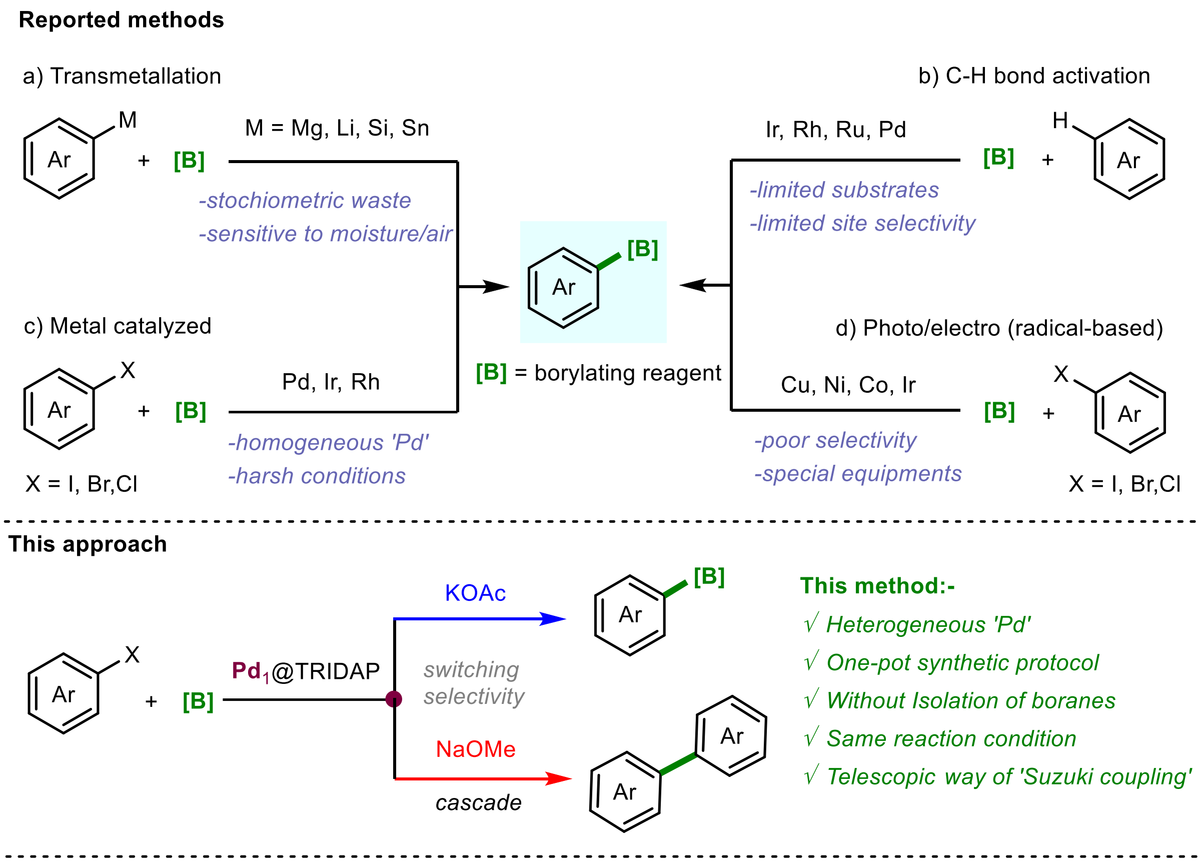


**Scheme S1.** Literature precedents on the borylation and comparison with the self-cascade approach.

**Table S6**. Comparisons of reported catalytic activity of the Miyaura borylation reaction and cascade coupling using $Pd_1$@TRIDAP.

| **Catalyst** | **Type** | **Conditions** | **Yield (%)** | **Cascade coupling** | **Ref.** |
|---|---|---|---|---|---|
| **$Pd_1$@TRIDAP** (Recyclable for 6 cycles) | **Heterogeneous** | KOAc or NaOMe, 80°C, 6h | **90** | ✓ | **This work** |
| **(Allyl)PdCl**/XPhos (0.5 mol%) | Homogeneous | 2-KEH, IPAc, 35-60°C, 1-20h | 99 | ✗ | 9 |
| **Pd-phenethylamine Cl** (2 mol%)/X-Phos | Homogeneous | KOAc, 80°C, EtOH, 18h | 90 | *Two steps* | 10 |
| **$Pd(OAc)_2$/XPhos** (0.5 mol%) | Homogeneous | KOAc, 80°C, EtOH, 20-30h | 93 | *Two steps* | 11 |
| **$PdCl_2$(dppf)/$Et_3$N** (3 mol%) | Homogeneous | Dioxane, 80°C, 2h | 89 | ✗ | 12 |
| **$Pd_2(dba)_3$/n-BuPA$d_2$** (1-6 mol%) | Homogeneous | KOAc, DMAc, 90°C, 2-6h | 98 | *Two steps* | 14 |
| **$Pd_2(dba)_3$/XPhos** (2-4 mol%) | Homogeneous | ChCl-Glycerol, 110°C, 1h | 90 | *Two steps* | 16 |
| **$PdCl_2(amphos)_2$+Zinc Trimer $[Zn]_3$** (2 mol%) | Homogeneous | THF, 80°C, 3h | 88 | ✗ | 17 |
| **$Pd(PCy_3)_2Cl_2$** (0.04 mol%) | Homogeneous | *o*-Xylene, 110°C, 10h | 96 | ✗ | 18 |
| **$PdCl_2$(dppf) (3 mol%)** | Homogeneous | DMSO, KOAc, 80°C, 2-24h | 98 | ✗ | 19 |
| **$Pd_2(dba)_3$/XPhos** (0.3 mol%) | Homogeneous | KOAc, Dioxane, 110°C | 94 | *Two steps* | 20 |
| **Pd-BI-DIME** (Bedford's Pd, 1 mol%) | Homogeneous | DMAc, KOAc, 100°C, 12h | 96 | ✗ | 21 |
| **$Pd(OAc)_2$/CuI/$PPh_3$** (1.2 mol%) | Homogeneous | THF, $Cs_2CO_3$, rt, 24h | 76 (97) | ✗ | 22 |
| **$Pd(OAc)_2$ or $PdCl_2(py)_2$ on Silica-SMAP** (0.5 mol%), *non-recyclable* | Heterogeneous | $K_2CO_3$ or CsOAc, Dioxane, 110°C | 84-99 | ✗ | 23 |
| **$Pd_2(dba)_3$/CM-Phos ligand** (0.5 mol%) | Homogeneous | CsOAc, Dioxane, 110°C, 20h | 96 | ✗ | 24 |
| **Bedford-type Palladacycle** (0.05 mol%) | Homogeneous | NaOAc, Water, 40°C, 6h | 99 | ✗ | 25 |
| **XPhos-Pd-G2** (2 mol%) | Homogeneous | KOAc, EtOH, 80°C, 2-15h | 94 | *Two steps* | 26 |
| **$Pd(OAc)_2$/AmPhos** (1 mol%) | Homogeneous | TPGS-750-M/$H_2O$, 55°C, 18h | 82 | *Two steps* | 27 |

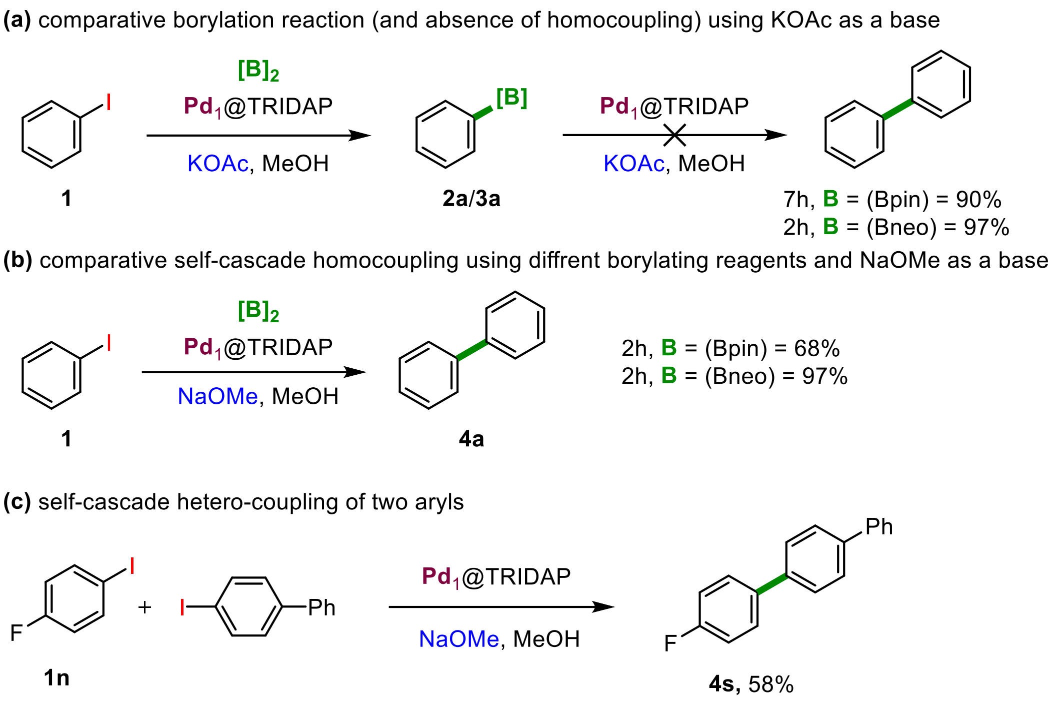


**Scheme S2.** Control experiments for the self-cascade coupling step, using KOAc as a base (a), using different diboron reagents (b), and heteroaryl coupling (c).

## 5. Reaction mechanisms

**Table S7.** Calculated entropic and zero-point energies for all reactants, products, and reaction intermediates.

| System | TS (eV) | ZPE (eV) | System | TS (eV) | ZPE (eV) |
|---|---|---|---|---|---|
| HBr | 0.41 | 0.19 | PhPh | 3.90 | 4.67 |
| NaBr | 0.42 | 0.08 | MeOH | 0.97 | 1.38 |
| KBr | 0.99 | 0.03 | MeONa | 0.47 | 0.81 |
| $B_2(OH)_4$ | 1.99 | 1.82 | MeOK | 1.52 | 1.05 |
| $B_2(pin)_2$ | 6.18 | 9.53 | MeOBOHOH | 1.76 | 2.05 |
| $B_2(neo)_2$ | 5.49 | 8.14 | MeOBpin | 3.99 | 5.89 |
| AcOH | 1.24 | 1.63 | MeOBneo | 3.47 | 5.23 |
| AcONa | 1.28 | 1.42 | AcO* | 0.82 | 1.36 |
| AcOK | 1.94 | 1.37 | Ph*Br* | 1.58 | 2.36 |
| AcOBOHOH | 2.42 | 2.27 | Ph*AcO* | 2.40 | 3.72 |
| AcOBpin | 5.57 | 6.15 | Ph*$B(OH)_2$* | 2.15 | 2.87 |
| AcOBneo | 3.94 | 5.46 | Ph*Bpin* | 4.28 | 7.13 |
| PhBr | 2.16 | 2.32 | Ph*Bneo* | 3.82 | 6.43 |
| $PhB(OH)_2$ | 1.98 | 3.27 | Ph*$CO_3$* | 2.30 | 0.00 |
| PhBpin | 4.96 | 7.13 | Ph*Ph* | 2.90 | 4.69 |
| PhBneo | 4.68 | 6.43 | PhBpinOMe* | 4.89 | 8.33 |
| $K_2CO_3$ | 2.09 | 0.49 | | | |

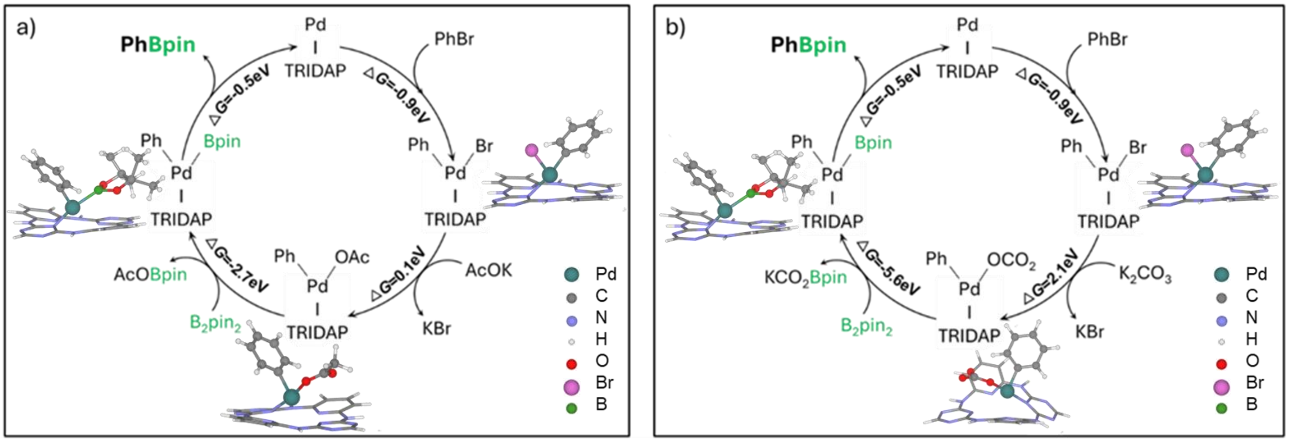


**Figure S9.** Comparative analysis of borylation reactions employing potassium acetate (KOAc) and potassium carbonate ($K_2CO_3$) as bases. Panel (a) presents the borylation reaction initiated with KOAc stabilizing intermediates and facilitating a controlled transmetalation step. The favorable reaction conditions facilitate the coupling of the organoboron reagent with the electrophile. In contrast, panel (b) illustrates the borylation reaction using $K_2CO_3$ as the base. The data demonstrate that $K_2CO_3$ is less effective as it is strong base and lack of ligand effect, as it fails to adequately promote the formation of the desired borylated products due to its inability to stabilize the palladium complex required for the reaction.

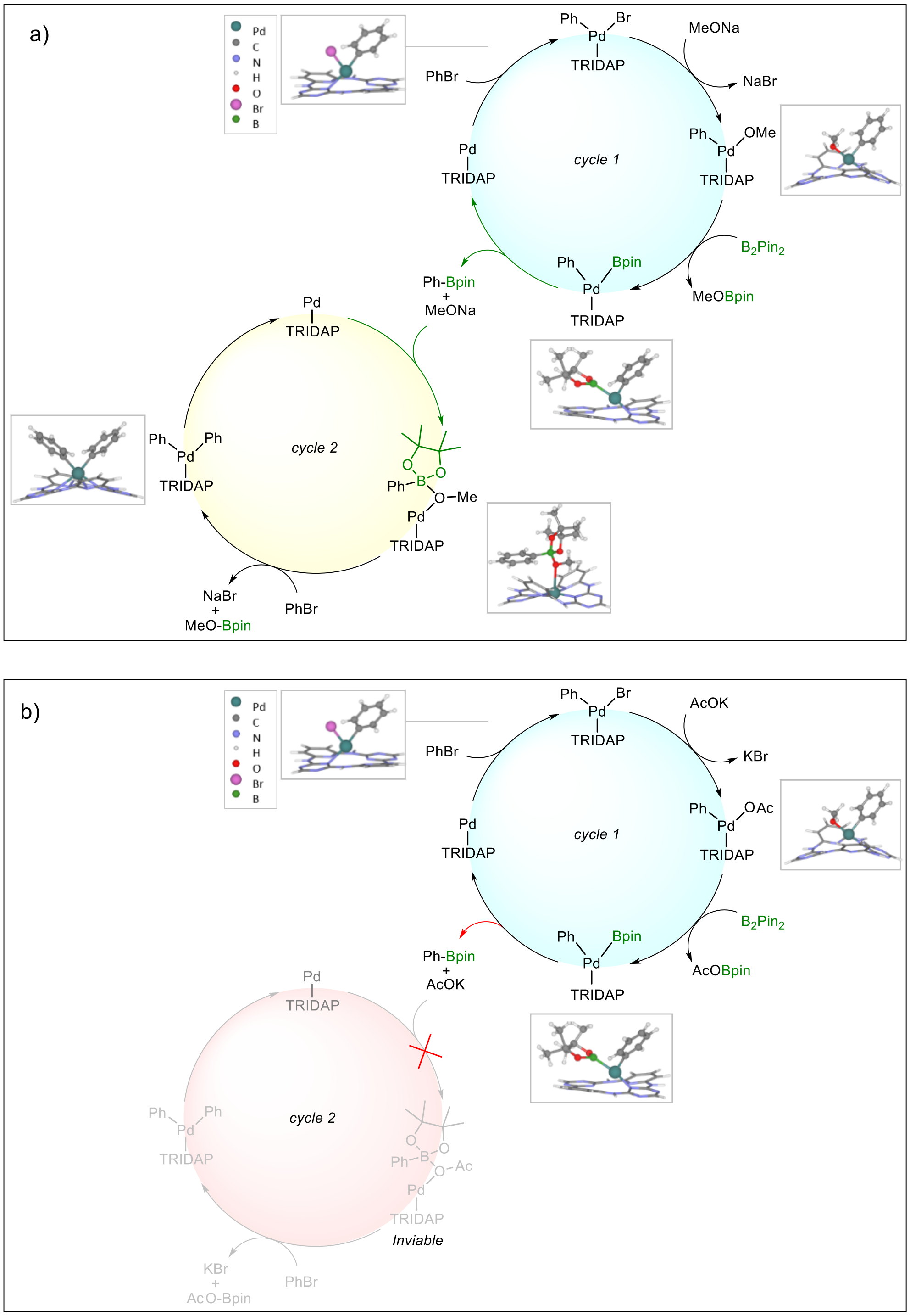


**Figure S10.** Mechanism for the Sukuzi-Miyaura cascade reaction by using NaOMe (a) and KOAc (b) bases, respectively.

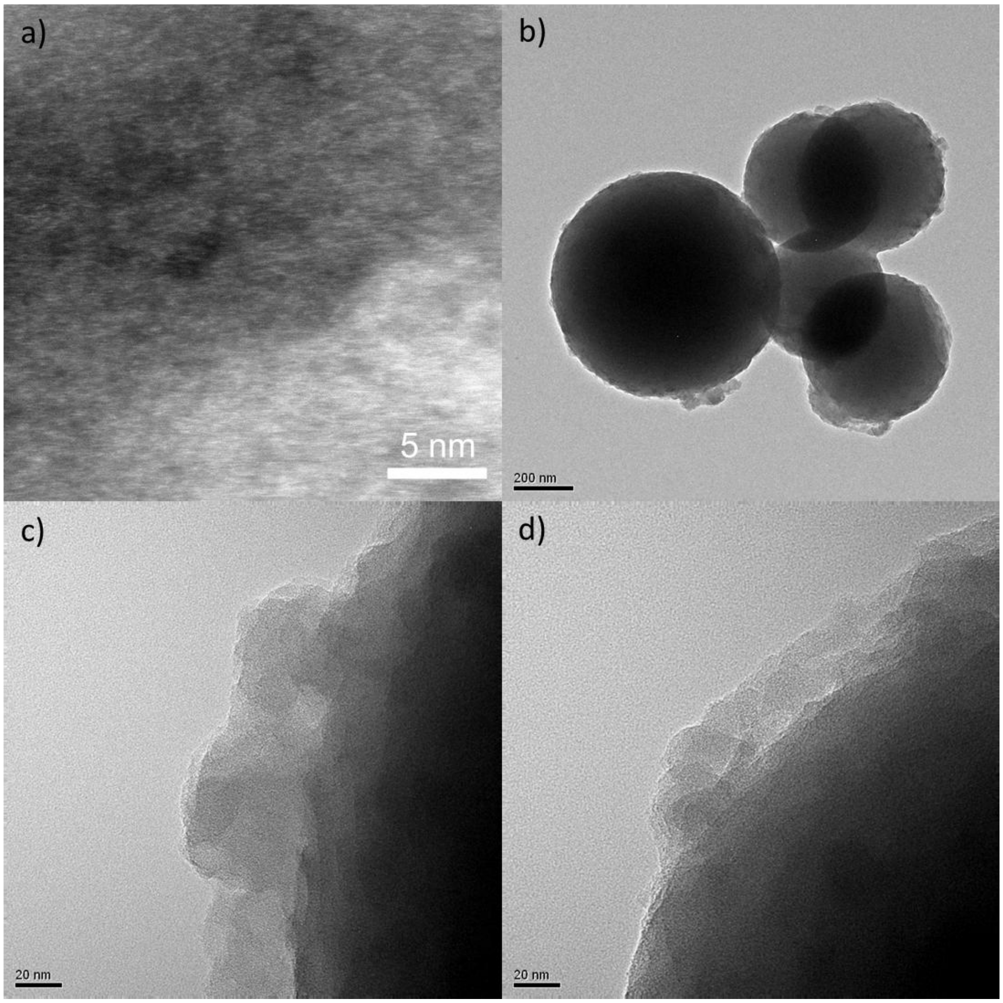


**Figure S11.** Microscopic analysis of $Pd_1$@TRIDAP catalyst: a) AC-HAADF-STEM image of recycled catalyst. b) TEM of fresh $Pd_1$@TRIDAP; (c, d) Recycled $Pd_1$@TRIDAP.

## 6. NMR spectral data of the all products

All compounds reported below are consistent with the structures reported previously in the literature.[2-7]

*4,4,5,5-tetramethyl-2-phenyl-1,3,2-dioxaborolane* **(2a)**. **$^1$H NMR (400 MHz, CDCl$_3$):** δ 7.74 (dd, *J* = 8.1, 1.5 Hz, 2H), 7.41 – 7.35 (m, 1H), 7.32 – 7.26 (m, 2H), 1.28 (s, 12H) **$^{13}$C NMR (101 MHz, CDCl$_3$):** δ 134.74, 131.23, 127.70, 83.77, 24.87. **m/z**≈ 204, 203, 189, 173, 161, 147, 131, 118, **105** (100%), 104, 85, 77, 59, 57, 51, 41, 39, 27.

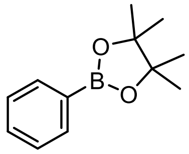


*4,4,5,5-tetramethyl-2-(p-tolyl)-1,3,2-dioxaborolane* **(2b). $^1$H NMR (400 MHz, CDCl$_3$):** δ 7.63 (d, *J* = 8.1 Hz, 2H), 7.11 (d, *J* = 8.2 Hz, 2H), 2.29 (s, 3H), 1.26 (s, 12H). **$^{13}$C NMR (101 MHz, CDCl$_3$):** δ 141.38, 134.81, 128.51, 83.61, 24.86, 21.71. **m/z**≈ 218.2, 203.1, 132.1, **119.0** (100%), 91, 53.

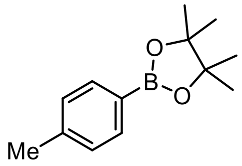


*4,4,5,5-tetramethyl-2-(m-tolyl)-1,3,2-dioxaborolane* **(2c). $^1$H NMR (400 MHz, CDCl$_3$):** δ 7.56 (s, 1H), 7.55 – 7.51 (m, 1H), 7.19 (dd, J = 4.9, 1.2 Hz, 2H), 2.28 (s, 3H), 1.27 (s, 12H). **$^{13}$C NMR (101 MHz, CDCl$_3$):** δ 137.10, 135.35, 132.03, 131.80, 127.68, 83.72, 24.87, 21.26. **m/z**≈ 218.1, 203.1, 187.1, 175.0, 145.0, **132** (100%), 110 (100%), 91, 85, 77, 65, 41.1.

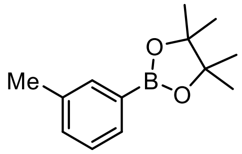


*4,4,5,5-tetramethyl-2-(o-tolyl)-1,3,2-dioxaborolane* **(2d). $^1$H NMR (400 MHz, CDCl$_3$):** δ 7.68 (dd, *J* = 7.7, 1.6 Hz, 1H), 7.24 (td, *J* = 7.5, 1.6 Hz, 1H), 7.12 – 7.06 (m, 2H), 2.46 (s, 3H), 1.27 (s, 12H). **$^{13}$C NMR (101 MHz, CDCl$_3$):** δ 144.82, 135.85, 130.76, 129.77, 124.69, 83.50, 24.54, 22.19. **m/z** ≈ 218.1, 203.1, 187.1, 174.1, 161 (90%), **119** (100%), 117, 91, 85, 41.

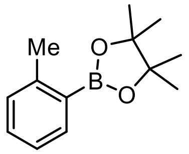


*4-(4,4,5,5-tetramethyl-1,3,2-dioxaborolan-2-yl)phenol* **(2e). $^1$H NMR (400 MHz, CDCl$_3$):** δ 7.63 (d, *J* = 8.4 Hz, 2H), 6.75 (d, *J* = 8.5 Hz, 2H), 5.50 (s, 1H), 1.26 (s, 12H). **$^{13}$C NMR (101 MHz, CDCl$_3$):** δ 158.49, 136.78, 114.84, 82.32, 24.56. **m/z**≈ **220** (90%), 205, 176, 162.9, 134, 126.8, **120** (100%), 107, 77, 65, 59, 41.

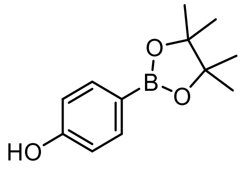

*2-(4-methoxyphenyl)-4,4,5,5-tetramethyl-1,3,2-dioxaborolane* **(2f).** **$^{1}$H NMR (400 MHz, $CDCl_3$):** δ 7.71 – 7.65 (m, 2H), 6.84 – 6.79 (m, 2H), 3.75 (s, 3H), 1.26 (s, 12H). **$^{13}$C NMR (101 MHz, $CDCl_3$):** δ 162.18, 136.52, 113.33, 83.55, 55.10, 24.88. **m/z**≈ 234.2, 219.1, 303.1, 191.1, 176.1, 161.1, 148.1, **134.1** (100%), 120, 109.3, 104.1, 85.1, 91.1, 77.1, 85.1, 41.1.

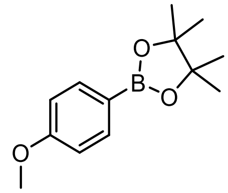


*4-(4,4,5,5-tetramethyl-1,3,2-dioxaborolan-2-yl)benzaldehyde* **(2i).** **$^{1}$H NMR (400 MHz, $CDCl_3$):** δ 9.98 (s, 1H), 7.89 (d, *J* = 8.1 Hz, 2H), 7.79 (d, *J* = 8.2 Hz, 2H), 1.29 (s, 12H). **$^{13}$C NMR (101 MHz, $CDCl_3$):** δ 192.63, 138.15, 136.03, 128.69, 84.34, 24.88. **m/z**≈ 232.1, **217.1** (100%), 201, 189, 146, 133, 103, 85, 43.

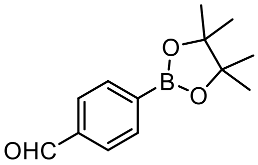


*Methyl 4-(4,4,5,5-tetramethyl-1,3,2-dioxaborolan-2-yl)benzoate* **(2l).** **$^{1}$H NMR (400 MHz, $CDCl_3$):** δ 7.76 (d, *J* = 8.6 Hz, 2H), 7.59 (d, *J* = 8.6 Hz, 2H), 2.54 (s, 3H), 1.29 (s, 12H). **$^{13}$C NMR (101 MHz, $CDCl_3$):** δ 167.33 δ 136.62, 134.92, 129.01, 84.22, 52.13, 24.88. **m/z**≈ 262.1, 255.2, 247.1, 231.1, 219.1, 205.1, 185.1, 176.1, 173.1, **163.1** (100%), 145.1, 131.1, 117.1, 103, 84.1, 59.1, 43.1.

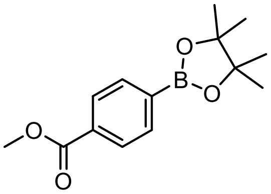


*2-(4-chlorophenyl)-4,4,5,5-tetramethyl-1,3,2-dioxaborolane* **(2m)**. **$^{1}$H NMR (400 MHz, $CDCl_3$)**: δ 7.65 (d, *J* = 8.4 Hz, 2H), 7.40 (d, *J* = 8.8 Hz, 2H), 1.26 (s, 12H). **$^{13}$C NMR (101 MHz, $CDCl_3$):** δ 138.46, 137.55, 136.13, 133.77, 129.05, 128.23, 128.01, 84.02, 24.87. **m/z**≈ 238.1, 223.1 (90%), 152.1, 139.1.

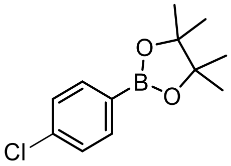


*2-(4-fluorophenyl)-4,4,5,5-tetramethyl-1,3,2-dioxaborolane* **(2n).** **$^{1}$H NMR (400 MHz, $CDCl_3$)**: δ 7.72 (dd, *J* = 8.5, 6.3 Hz, 2H), 7.00 – 6.93 (m, 2H), 1.26 (s, 12H). **$^{13}$C NMR (101 MHz, $CDCl_3$)**: δ 166.37, 163.88, 137.03, 136.95, 114.93, 114.73, 83.91, 24.86. **m/z**≈ 222.1, **207.1** (100%), 136.1, 123.1 (100%), 43.1.

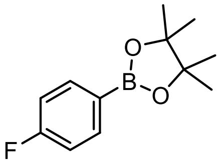

*4,4,5,5-tetramethyl-2-(4-(trifluoromethyl)phenyl)-1,3,2-dioxaborolane* **(2o).** **$^{1}$H NMR (400 MHz, $CDCl_3$):** δ 7.84 (d, *J* = 8.4 Hz, 2H), 7.53 (d, *J* = 7.6 Hz, 2H), 1.28 (s, 12H). **$^{13}$C NMR (101 MHz, $CDCl_3$):** δ 135.00, 132.68, 127.64, 126.01, 125.97, 125.93, 125.89, 124.37, 124.33, 124.29, 124.25, 122.79, 84.27, 24.86. **m/z**≈ 272.1, **257.1** (100%), 253.1, 241.1, 186.1, 173.1, 153, 127, 85, 43.1.

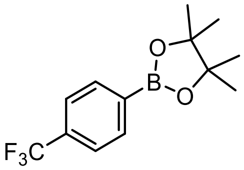


*2-([1,1'-biphenyl]-4-yl)-4,4,5,5-tetramethyl-1,3,2-dioxaborolane* **(2r).** **$^{1}$H NMR (400 MHz, $CDCl_3$):** δ 7.81 (d, *J* = 8.3 Hz, 2H), 7.57 – 7.50 (m, 4H), 7.36 (d, *J* = 1.7 Hz, 2H), 7.26 (t, 1H), 1.29 (s, 12H). **$^{13}$C NMR (101 MHz, $CDCl_3$):** δ 143.91, 141.06, 135.27, 128.77, 127.56, 127.25, 126.91, 126.47, 83.83, 24.90. **m/z**≈ 280.2, 265.2, 223.1, 194.1, **180.1** (100%), 163.1, 152.1, 128.1, 41.1.

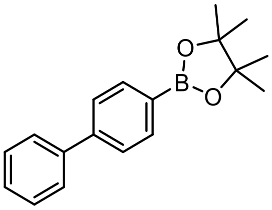


*2-([1,1'-biphenyl]-2-yl)-4,4,5,5-tetramethyl-1,3,2-dioxaborolane* **(2s).** **$^{1}$H NMR (400 MHz, $CDCl_3$):** δ 7.66 – 7.61 (m, 1H), 7.36 – 7.24 (m, 8H), 1.13 (s, 12H). **$^{13}$C NMR (101 MHz, $CDCl_3$):** δ 147.56, 143.30, 134.45, 129.28, 129.16, 128.76, 127.96, 127.76, 127.18, 126.83, 126.28, 83.73, 24.61. **m/z**≈ 280.2, 265.1, 194.1, 180.1, **164.1** (100%), 152.1, 128, 77, 41.1.

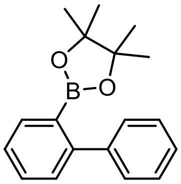


*1,4-bis(4,4,5,5-tetramethyl-1,3,2-dioxaborolan-2-yl)benzene* **(2t).** **$^{1}$H NMR (400 MHz, $CDCl_3$):** δ 7.73 (s, 4H), 1.28 (s, 24H). **$^{13}$C NMR (101 MHz, $CDCl_3$):** δ 133.88, 83.84, 25.03. **m/z**≈330.2, 315.2, 287.2, 273.2, 244.2, **231.1** (100%), 215.1, 202.1, 188.1, 173.1, 158.1, 144.1, 117.1, 101.1, 85.1, 77.1, 69.1, 59.1, 55.1, 43.1.

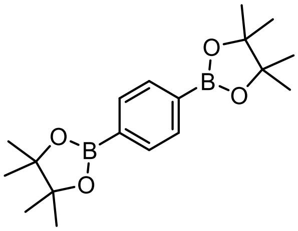

*2-(4-bromophenyl)-4,4,5,5-tetramethyl-1,3,2-dioxaborolane* **(2u).** **$^{1}$H NMR (400 MHz, $CDCl_3$):** δ 7.61 – 7.56 (m, 2H), 7.45 – 7.41 (m, 2H), 1.26 (s, 12H). **$^{13}$C NMR (101 MHz, $CDCl_3$):** δ 136.31, 130.96, 126.23, 84.04, 24.86.

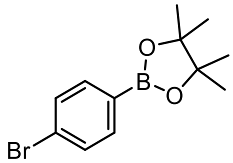


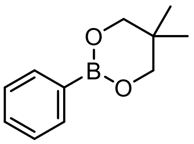


*5,5-dimethyl-2-phenyl-1,3,2-dioxaborinane* **(3a).** **$^{1}$H NMR (400 MHz, $CDCl_3$):** δ 7.74 – 7.70 (m, 2H), 7.38 – 7.33 (m, 1H), 7.31 – 7.25 (m, 2H), 3.70 (s, 4H), 0.95 (s, 6H). **$^{13}$C NMR (101 MHz, $CDCl_3$):** δ 135.65, 133.83, 130.66, 127.57, 72.33, 31.89, 21.92. **m/z**≈ 218, 203, 190, 189, 174, 161, 146, 132, 117, 103, 91, 91, 77, 63, 43, 30.

*2-(4-fluorophenyl)-5,5-dimethyl-1,3,2-dioxaborinane* **(3b).** **$^{1}$H NMR (400 MHz, $CDCl_3$):** δ 7.71 (dd, *J* = 8.5, 6.4 Hz, 2H), 6.95 (t, *J* = 8.9 Hz, 2H), 3.69 (s, 4H), 0.95 (s, 6H). **$^{13}$C NMR (101 MHz, $CDCl_3$):** δ 166.09, 163.62, 136.03, 135.95, 114.68, 114.48, 72.32, 31.88, 21.89. **m/z**≈ **208.1** (100%), 193, 178.1, 165.0, 123, 109, 56.

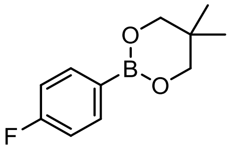


*2-(4-chlorophenyl)-5,5-dimethyl-1,3,2-dioxaborinane* **(3c).** **$^{1}$H NMR (400 MHz, $CDCl_3$):** δ 7.65 (d, *J* = 8.3 Hz, 2H), 7.24 (d, *J* = 8.3 Hz, 2H), 3.68 (s, 4H), 0.94 (s, 6H). **$^{13}$C NMR (101 MHz, $CDCl_3$):** δ 136.89, 135.26, 129.05, 128.23, 127.82, 72.34, 31.89, 21.89. **m/z**≈ 226.1, **224.1** (100%), 181, 139, 125, 56.1.

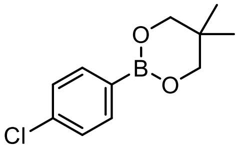


*5,5-dimethyl-2-(4-(trifluoromethyl)phenyl)-1,3,2-dioxaborinane* **(3d).** **$^{1}$H NMR (400 MHz, $CDCl_3$):** δ 8.70 – 8.64 (m, 1H), 7.96 (dd, *J* = 6.9, 1.4 Hz, 1H), 7.81 (dd, *J* = 8.3, 1.2 Hz, 1H), 7.75 (s, 1H), 7.42 – 7.38 (m, 3H), 3.81 (s, 4H), 1.02 (s, 6H). **$^{13}$C NMR (101 MHz, $CDCl_3$):** δ 134.10, 132.45, 132.13, 127.64, 125.97, 125.93, 124.18, 124.14, 124.10, 72.40, 31.90, 21.86. **m/z**≈ **258.1** (100%), 243.1, 239.1, 228.1, 215.1, 200.1, 187.1, 173, 56.1.

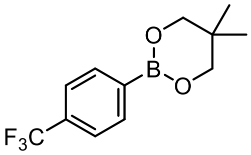

*5,5-dimethyl-2-(naphthalen-1-yl)-1,3,2-dioxaborinane* **(3e).** **$^{1}$H NMR (400 MHz, $CDCl_3$):** δ 8.70 – 8.64 (m, 1H), 7.96 (dd, *J* = 6.9, 1.4 Hz, 1H), 7.81 (dd, *J* = 8.3, 1.2 Hz, 1H), 7.75 (s, 1H), 7.42 – 7.38 (m, 3H), 3.81 (s, 4H), 1.02 (s, 6H). **$^{13}$C NMR (101 MHz, $CDCl_3$):** δ 136.75, 134.35, 133.43, 130.92, 128.43, 128.36, 127.89, 126.57, 125.98, 125.23, 72.51, 31.80, 21.96. **m/z≈ 240.2** (100%), 197.1, 181.1, 167.1, 154.1 (90%), 141.1, 127.1, 77.1, 55.1, 41.1.

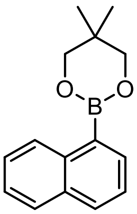


*2-([1,1'-biphenyl]-4-yl)-5,5-dimethyl-1,3,2-dioxaborinane* **(3f).** **$^{1}$H NMR (400 MHz, $CDCl_3$):** δ 7.80 (d, *J* = 8.2 Hz, 2H), 7.57 – 7.50 (m, 4H), 7.36 (t, *J* = 6.0 Hz, 2H), 7.26 (t, 1H), 3.72 (s, 4H), 0.96 (s, 6H). **$^{13}$C NMR (101 MHz, $CDCl_3$):** δ 143.30, 141.25, 134.36, 128.76, 128.73, 127.22, 126.35, 72.37, 31.93, 21.93. **m/z≈** 267, **266.2** (100%), 223.1, 195.1, 180.1, 167.1, 152.1, 56.1.

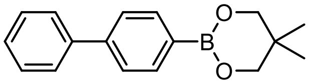


*1,1'-biphenyl* **(4a).** **$^{1}$H NMR (400 MHz, $CDCl_3$):** δ 7.56 – 7.49 (m, 4H), 7.40 – 7.33 (m, 4H), 7.30 – 7.24 (m, 2H). **$^{13}$C NMR (101 MHz, $CDCl_3$):** δ 141.27, 128.75, 127.17. **m/z≈ 154** (100%), 153, 139, 128, 122, 115, 113, 102, 76, 63, 51.

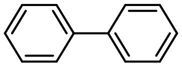

*[1,1'-biphenyl]-4,4'-dicarbaldehyde* **(4c).** **$^{1}$H NMR (400 MHz, $CDCl_3$):** δ 10.02 (s, 2H), 7.93 (d, *J* = 8.3 Hz, 4H), 7.73 (d, *J* = 8.3 Hz, 4H). **$^{13}$C NMR (101 MHz, $CDCl_3$):** δ 191.66, 145.58, 136.04, 130.36, 128.04. **m/z≈** 210, **209** (100%), 181, 163, 152, 126, 102, 74, 50.

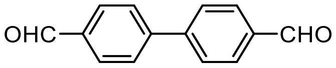


*1,1'-([1,1'-biphenyl]-4,4'-diyl)bis(ethan-1-one)* **(4d).** **$^{1}$H NMR (400 MHz, $CDCl_3$):** δ 7.99 (d, *J* = 8.7 Hz, 4H), 7.65 (d, *J* = 8.7 Hz, 4H), 2.58 (s, 6H). **$^{13}$C NMR (101 MHz, $CDCl_3$):** δ 197.56, 144.36, 136.62, 129.01, 127.46, 26.68. **m/z≈** 238, **223** (100%), 195, 180, 165, 152, 139, 126, 104, 90, 76, 63, 50, 43.

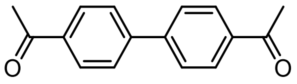


*4,4'-difluoro-1,1'-biphenyl* **(4f).** **$^{1}$H NMR (400 MHz, $CDCl_3$):** δ 7.41 (dd, *J* = 8.8, 5.3 Hz, 4H), 7.04 (t, *J* = 8.7 Hz, 4H). **$^{13}$C NMR (101 MHz, $CDCl_3$):** δ 163.67, 161.22, 136.43, 136.40, 128.61, 128.53,

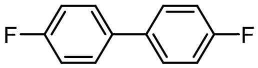

115.79, 115.57. **m/z**≈ **190** (100%), 189, 188, 170, 164, 157, 151, 133, 120, 98, 95, 94, 85, 82, 75, 70, 63, 57, 50, 39, 31.

*4,4'-dichloro-1,1'-biphenyl* **(4g).** **$^1$H NMR (400 MHz, CDCl$_3$):** δ 7.40 (d, *J* = 8.6 Hz, 4H), 7.33 (d, *J* = 8.5 Hz, 4H). **$^{13}$C NMR (101 MHz, CDCl$_3$):** δ 191.66, 145.58, 136.04, 130.36, 128.04. **m/z**≈ 226, 224, **222** (100%), 188, 186, 173, 152 (90%), 151, 136, 126, 111, 99, 93, 87, 80, 75, 63, 50, 39.

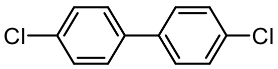


*4,4'-bis(trifluoromethyl)-1,1'-biphenyl* **(4h).** **$^1$H NMR (400 MHz, CDCl$_3$):** δ 7.69 – 7.60 (m, 8H). **$^{13}$C NMR (101 MHz, CDCl$_3$):** δ 143.27, 127.64, 126.01, 125.97, 125.93. **m/z**≈ 290.1 (100%), 271.1, 240.1, 219.1, 201.1, 152.1, 145, **120.1** (100%), 69.

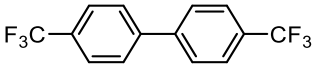


*4,4'-dimethoxy-1,1'-biphenyl* **(4k).** **$^1$H NMR (400 MHz, CDCl$_3$):** δ 7.42 – 7.37 (m, 4H), 6.91 – 6.85 (m, 4H), 3.76 (s, 6H). **$^{13}$C NMR (101 MHz, CDCl$_3$):** δ 158.74, 133.53, 127.74, 114.20, 55.36. **m/z≈214** (100%), 211, 200, 199, 183, 171, 168, 156, 152, 145, 139, 128, 115, 107, 102, 89, 75, 69, 63, 55, 43, 39.

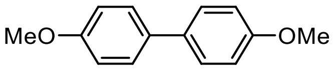


*3,3'-dimethyl-1,1'-biphenyl* **(4l).** **$^1$H NMR (400 MHz, CDCl$_3$)** δ 7.46 – 7.39 (m, 4H), 7.35 (td, *J* = 7.5, 0.8 Hz, 2H), 7.22 – 7.16 (m, 2H), 2.45 (s, 6H). **$^{13}$C NMR (101 MHz, CDCl$_3$)** δ 141.38, 138.25, 128.60, 127.99, 127.91, 124.29, 21.54. **m/z**≈ **182.2** (100%), 178.1, 167.1, 152.1, 139.1, 128.1, 115.1, 102.1, 89.1, 51.1.

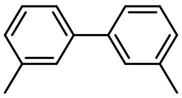

*1,1'-binaphthalene* **(4n).** **$^1$H NMR (400 MHz, CDCl$_3$):** δ 7.88 (dd, *J* = 8.2, 3.7 Hz, 4H), 7.52 (dd, *J* = 8.3, 7.0 Hz, 2H), 7.45-7.37 (m, 4H), 7.32 (dd, *J* = 8.6, 1.1 Hz, 2H), 7.25 – 7.19 (m, 2H). **$^{13}$C NMR (101 MHz, CDCl$_3$):** δ 138.47, 133.54, 132.87, 128.14, 127.88, 127.83, 126.56, 125.96, 125.79,

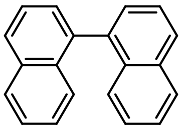

125.37. **m/z≈ 254** (100%), 253, 252, 250, 239, 226, 126, 119, 113, 100, 87, 74, 63, 51, 39, 18.

*4'-methoxy-[1,1'-biphenyl]-4-carbonitrile* **(4p).** **$^{1}$H NMR (400 MHz, $CDCl_3$)** δ 7.61 (d, J = 8.5 Hz, 2H), 7.56 (d, J = 8.7 Hz, 2H), 7.46 (d, J = 8.8 Hz, 2H), 6.93 (d, J = 8.8 Hz, 2H), 3.79 (s, 3H). **$^{13}$C NMR (101 MHz, $CDCl_3$)** δ 160.25, 145.25, 132.89, 132.58, 131.54, 128.37, 127.94, 127.13, 119.08, 114.59, 110.16, 55.42. **m/z≈** 214.1, **209 (100%),** 199,180,166, 156, 140, 128, 113, 102.1, 89.1, 75, 63, 50.1.

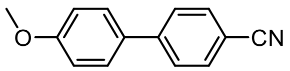


*4-methyl-1,1':4',1''-terphenyl* **(4q).** **$^{1}$H NMR** (400 MHz, $CDCl_3$) δ 7.68 (d, J = 12.3 Hz, 6H), 7.58 (d, J = 7.8 Hz, 2H), 7.51 – 7.46 (m, 3H), 7.40 (d, J = 7.0 Hz, 2H), 7.33 – 7.27 (m, 3H), 2.44 (s, 3H). **$^{13}$C NMR** (101 MHz, $CDCl_3$) δ 140.81, 140.09, 139.86, 137.84, 137.15, 129.55, 128.81, 127.47, 127.30, 127.28, 127.04, 126.89, 21.13. **m/z≈244** (100%), 139.1, 228.1, 215.1, 202.1, 189.1, 176.1, 165.1, 152.1, 139, 101, 91, 77, 63, 51.

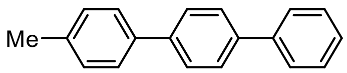


*4-fluoro-1,1':4',1''-terphenyl* **(4s)**. **$^{1}$H NMR (400 MHz, $CDCl_3$)** δ 7.62 – 7.49 (m, 8H), 7.39 (t, J = 7.7 Hz, 2H), 7.32 – 7.26 (m, 1H), 7.07 (t, J = 8.7 Hz, 2H). **$^{13}$C NMR (101 MHz, $CDCl_3$)** δ 163.77, 161.32, 140.62, 140.17, 139.16, 136.87, 136.83, 128.84, 128.63, 128.55, 127.56, 127.41, 127.36, 127.04, 115.80, 115.59. **m/z≈248.1** (100%), 233, 226.1, 220, 207, 200, 194, 183, 152, 144, 133, 120, 96, 87, 75, 63, 51.

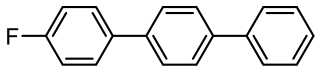


*4-chloro-4'-methoxy-1,1'-biphenyl* **(4r)**. **$^{1}$H NMR (400 MHz, $CDCl_3$)** δ 7.43 – 7.37 (m, 4H), 7.29 (d, J = 8.5 Hz, 2H), 6.91 (s, 1H), 6.88 (s, 1H), 3.77 (s, 3H). **$^{13}$C NMR (101 MHz, $CDCl_3$)** δ 159.41, 139.31, 132.53, 128.85, 128.02, 127.94, 114.34, 55.37.

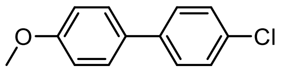


*4'-chloro-[1,1'-biphenyl]-2-amine* **(4t)**. **$^{1}$H NMR (400 MHz, $CDCl_3$)** δ 7.34 – 7.30 (m, 4H), 7.08 (td, J = 7.7, 1.7 Hz, 1H), 7.00 (dd, J = 7.6, 1.6 Hz, 1H), 6.74 (td, J = 7.5, 1.2 Hz, 1H), 6.68 (dd, J = 8.0, 1.2 Hz, 1H), 3.51 (s, 2H). **$^{13}$C NMR (101 MHz, $CDCl_3$)** δ 143.43, 137.97, 133.14, 130.48, 130.34, 129.06, 129.00, 128.84, 128.24, 126.35, 118.80, 115.75.

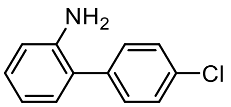

*4'-methyl-[1,1'-biphenyl]-2-carbonitrile* **(4u)**. **$^1$H NMR (400 MHz, $CDCl_3$)** δ 7.68 (dd, J = 7.7, 1.3 Hz, 1H), 7.54

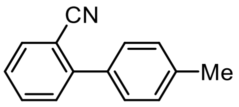


(dd, J = 7.6, 1.4 Hz, 1H), 7.45 – 7.32 (m, 4H), 7.26 – 7.20 (m, 2H), 7.19 (s, 1H), 2.35 (s, 3H). **$^{13}$C NMR (101 MHz, $CDCl_3$)** δ 145.58, 138.71, 135.30, 133.72, 132.73, 129.98, 129.45, 128.62, 127.26, 118.85, 111.26, 21.24.

## 7. NMR spectra of products

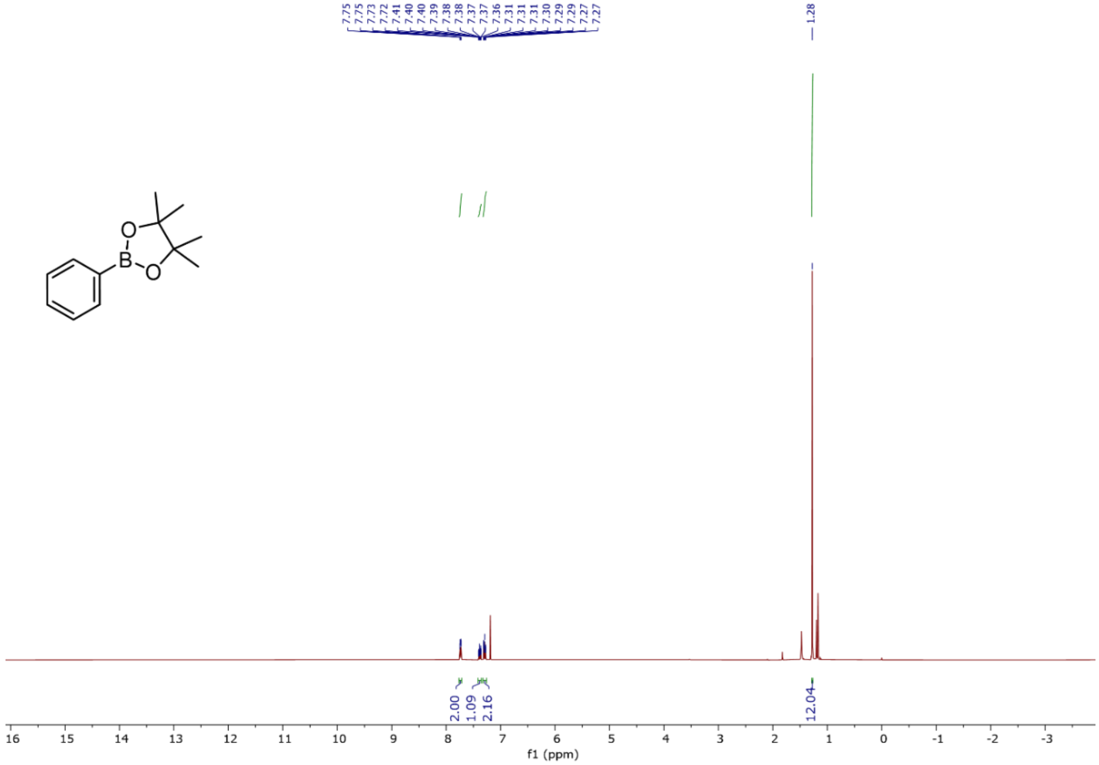


$^1$H NMR of *4,4,5,5-tetramethyl-2-phenyl-1,3,2-dioxaborolane* **(2a)**

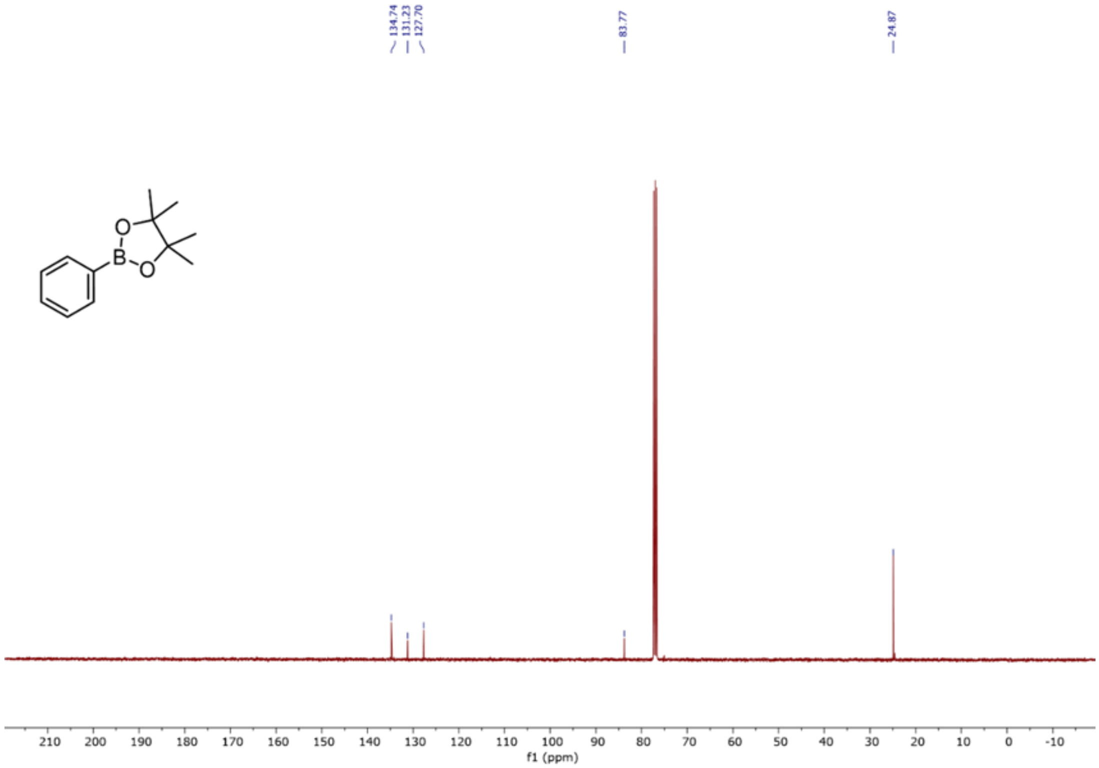

$^{13}$C NMR of *4,4,5,5-tetramethyl-2-phenyl-1,3,2-dioxaborolane* **(2a)**

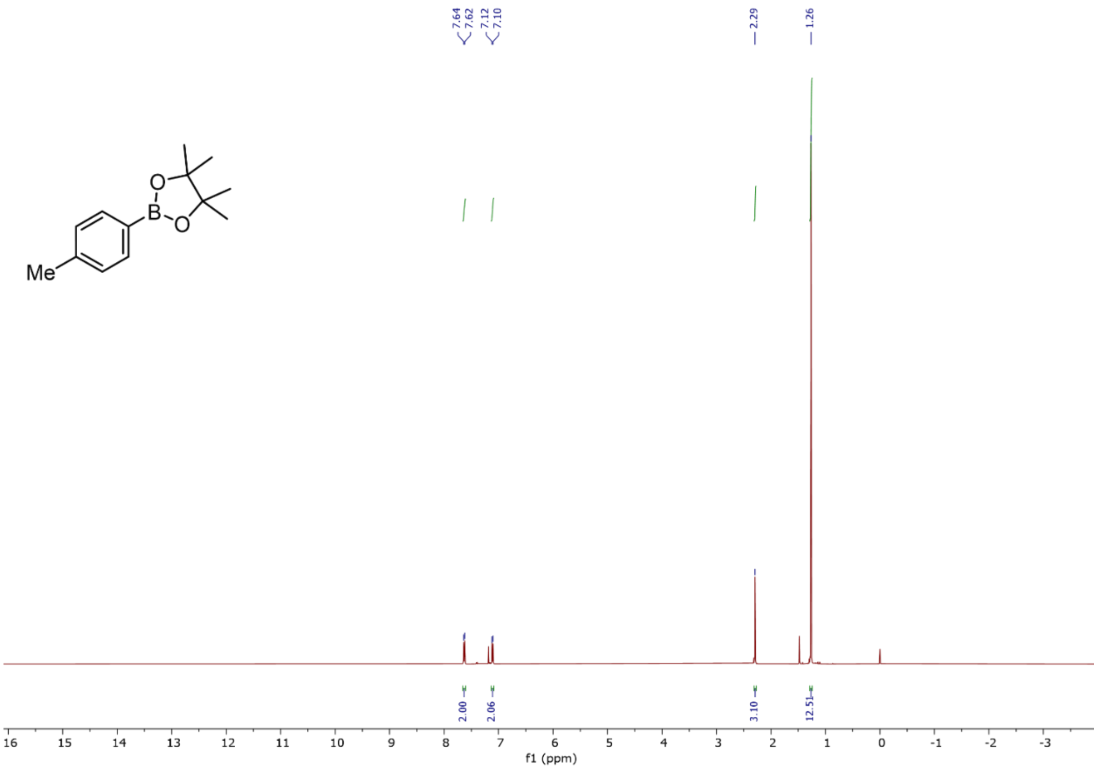


$^{1}$H NMR of *4,4,5,5-tetramethyl-2-(p-tolyl)-1,3,2-dioxaborolane* **(2b)**

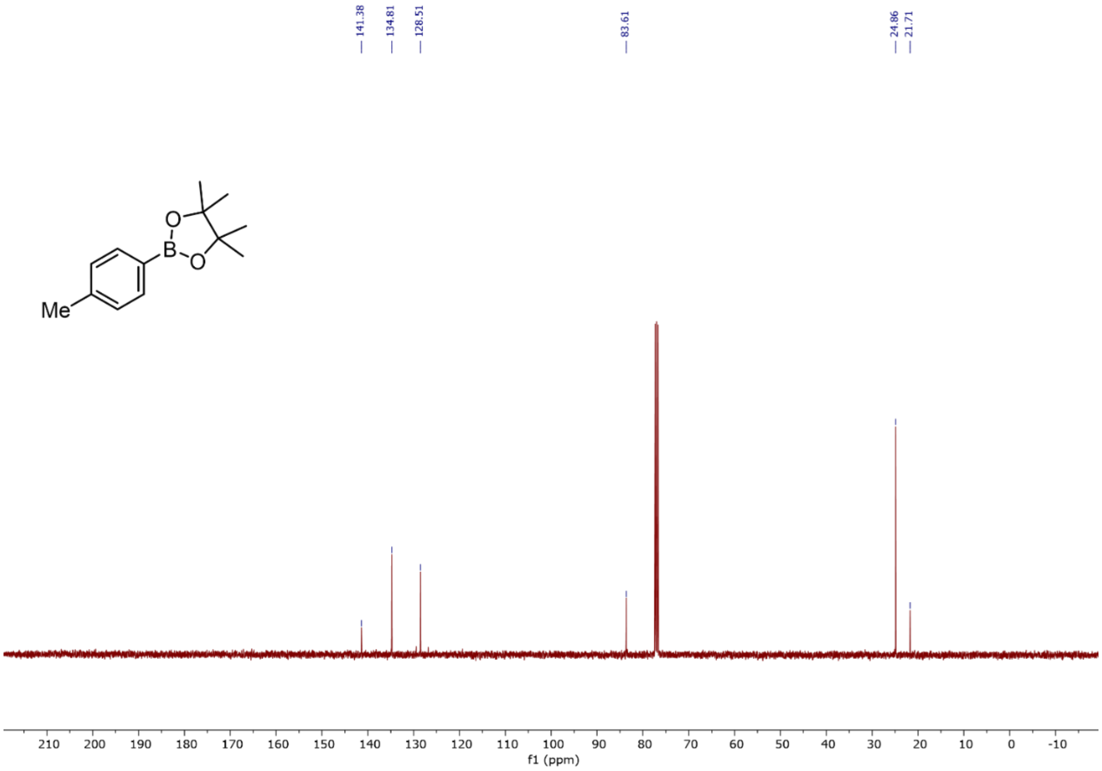

$^{13}C$ NMR of *4,4,5,5-tetramethyl-2-(p-tolyl)-1,3,2-dioxaborolane* **(2b)**

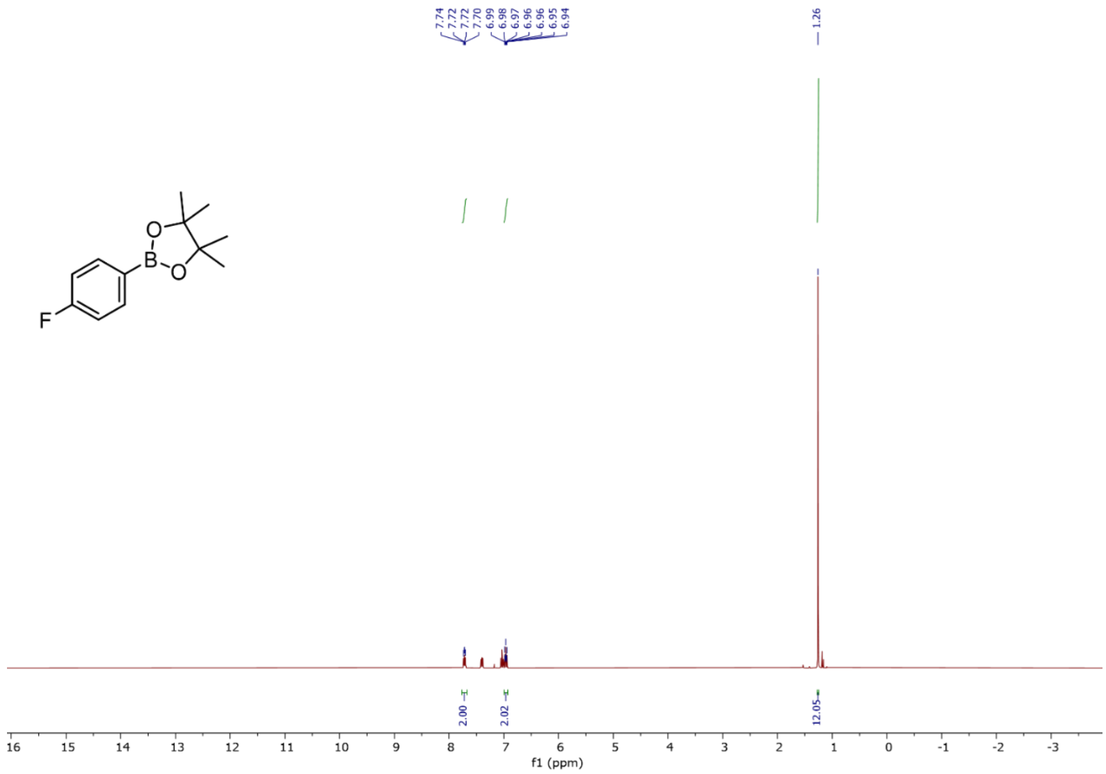


$^{1}H$ NMR of *2-(4-fluorophenyl)-4,4,5,5-tetramethyl-1,3,2-dioxaborolane* **(2n)**

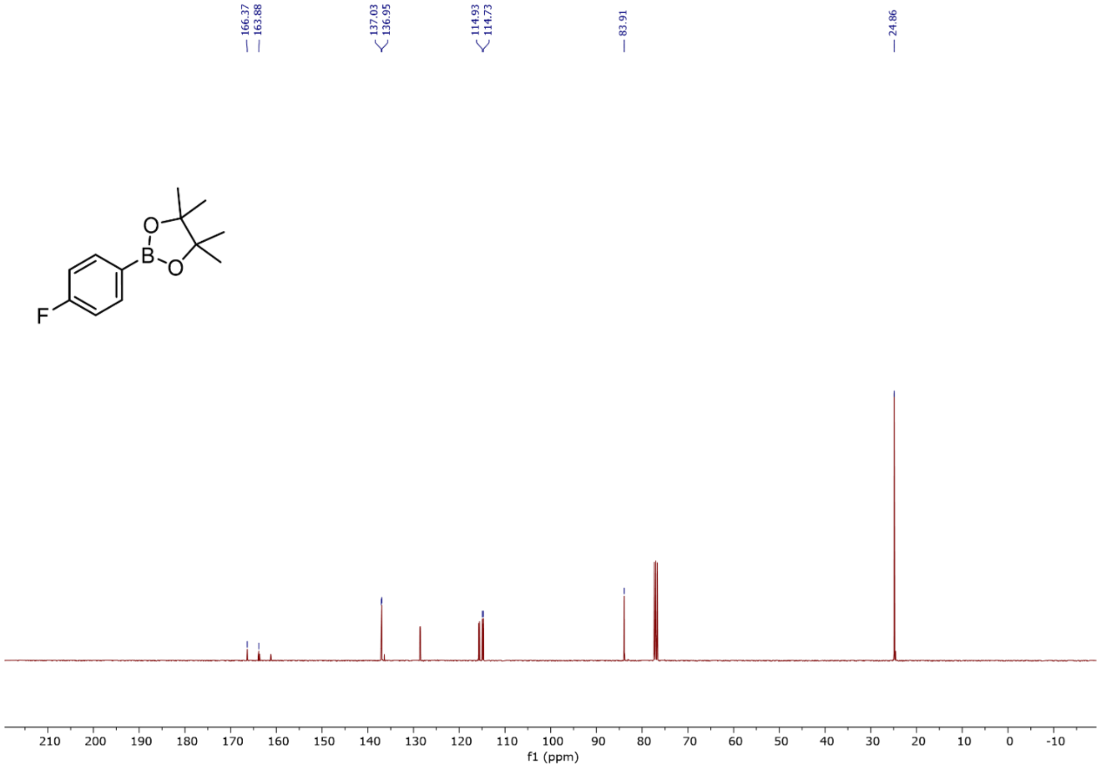


$^{13}C$ NMR of *2-(4-fluorophenyl)-4,4,5,5-tetramethyl-1,3,2-dioxaborolane* **(2n)**

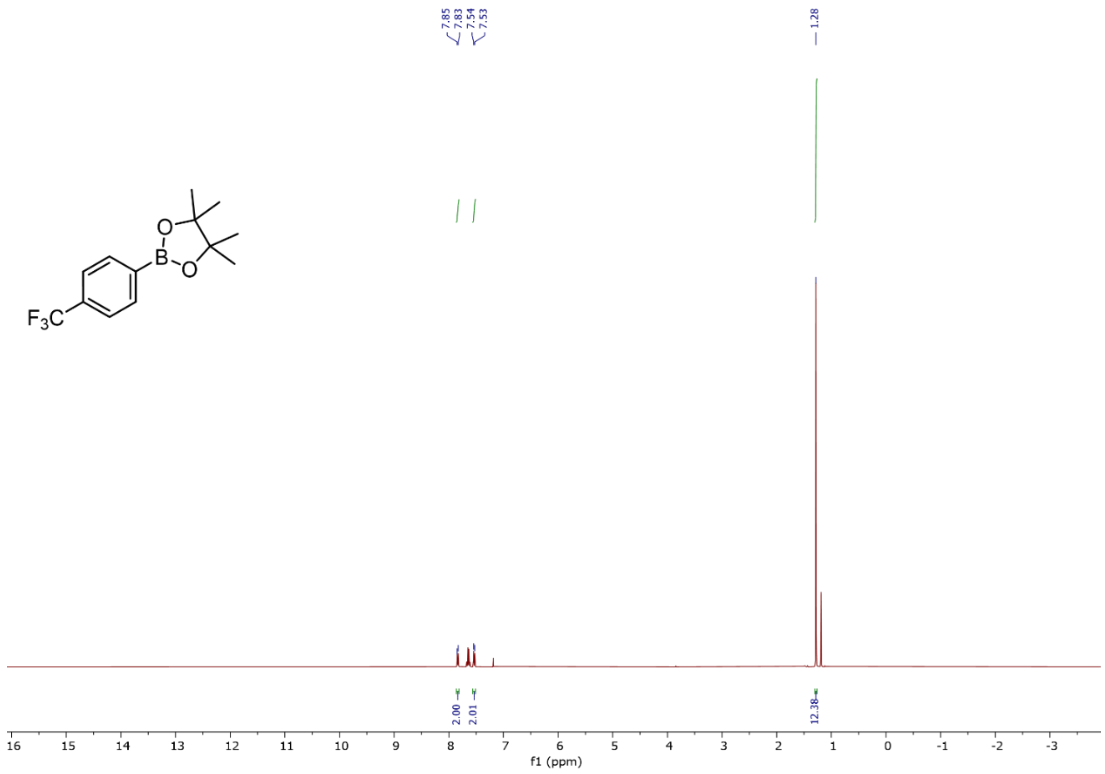


$^{1}$H NMR of *4,4,5,5-tetramethyl-2-(4-(trifluoromethyl)phenyl)-1,3,2-dioxaborolane* **(2o)**

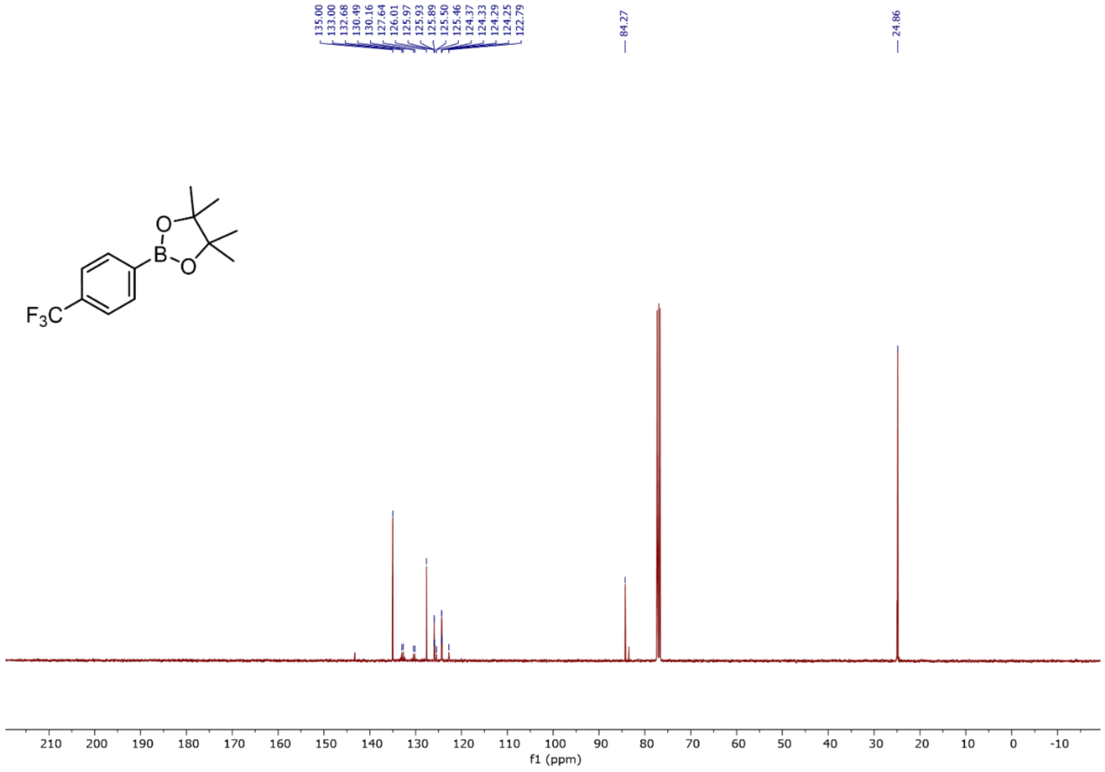


$^{13}$C NMR of *4,4,5,5-tetramethyl-2-(4-(trifluoromethyl)phenyl)-1,3,2-dioxaborolane* **(2o)**

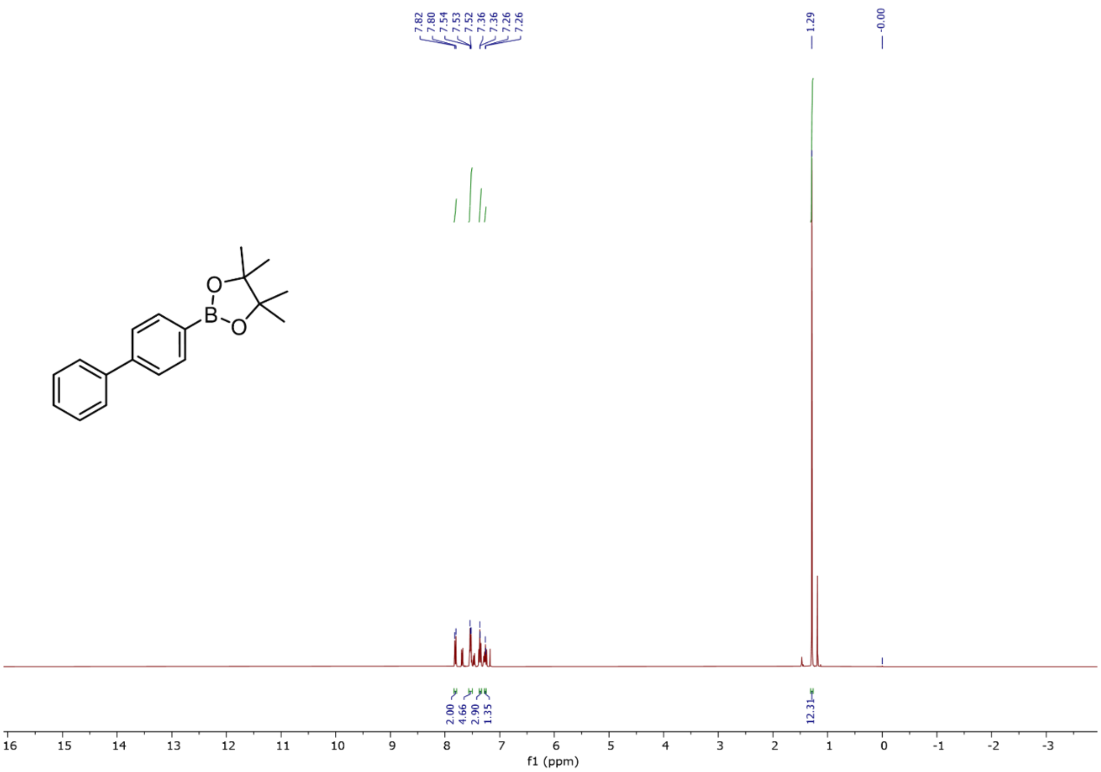


$^{1}$H NMR of *2-([1,1'-biphenyl]-4-yl)-4,4,5,5-tetramethyl-1,3,2-dioxaborolane* **(2r)**

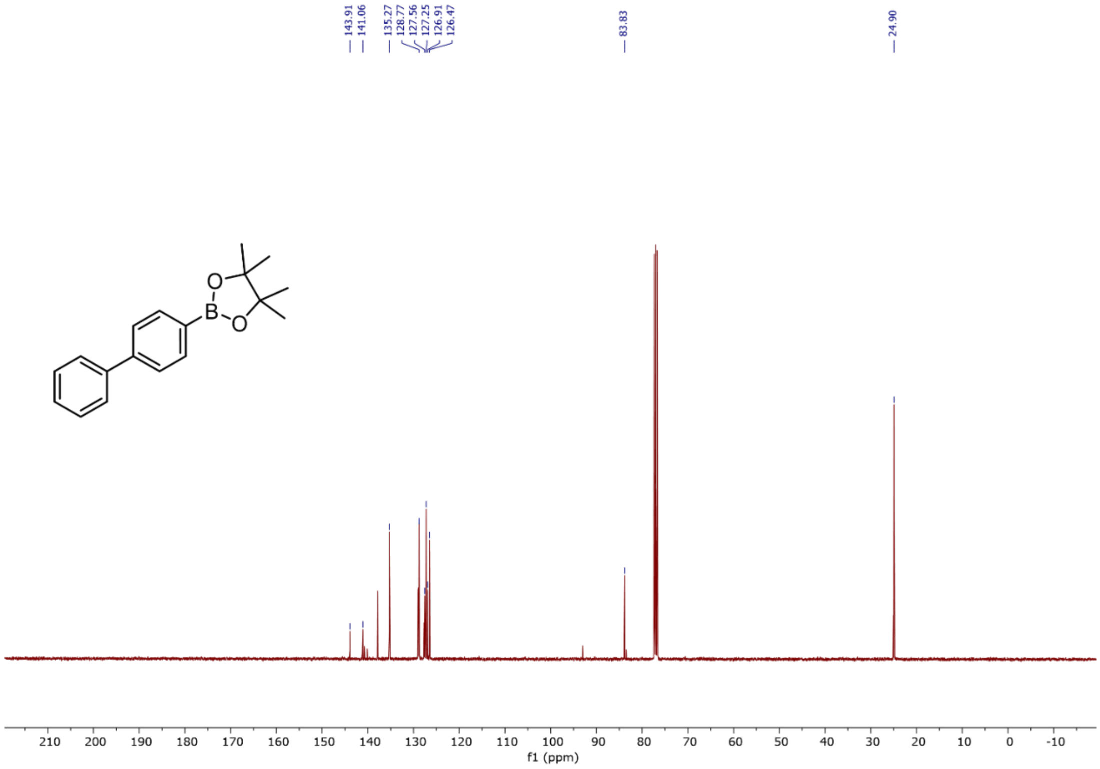


$^{13}$C NMR of *2-([1,1'-biphenyl]-4-yl)-4,4,5,5-tetramethyl-1,3,2-dioxaborolane* **(2r)**

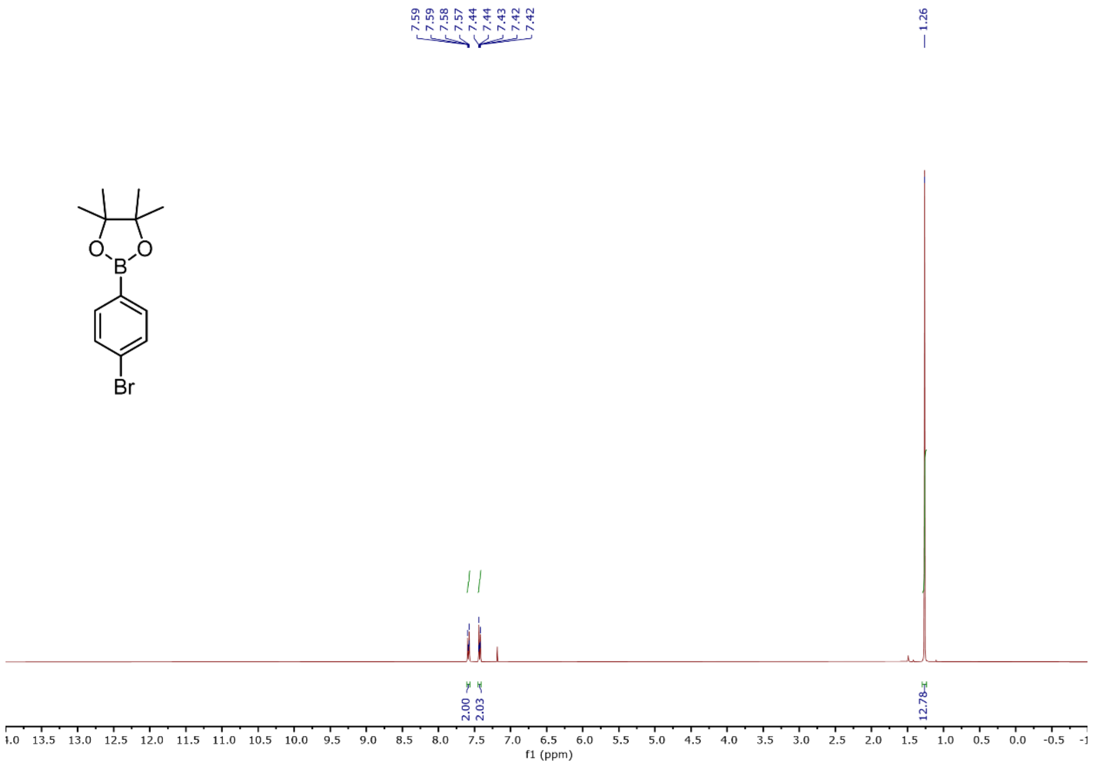


$^1$H NMR of *2-(4-bromophenyl)-4,4,5,5-tetramethyl-1,3,2-dioxaborolane* **(2u)**

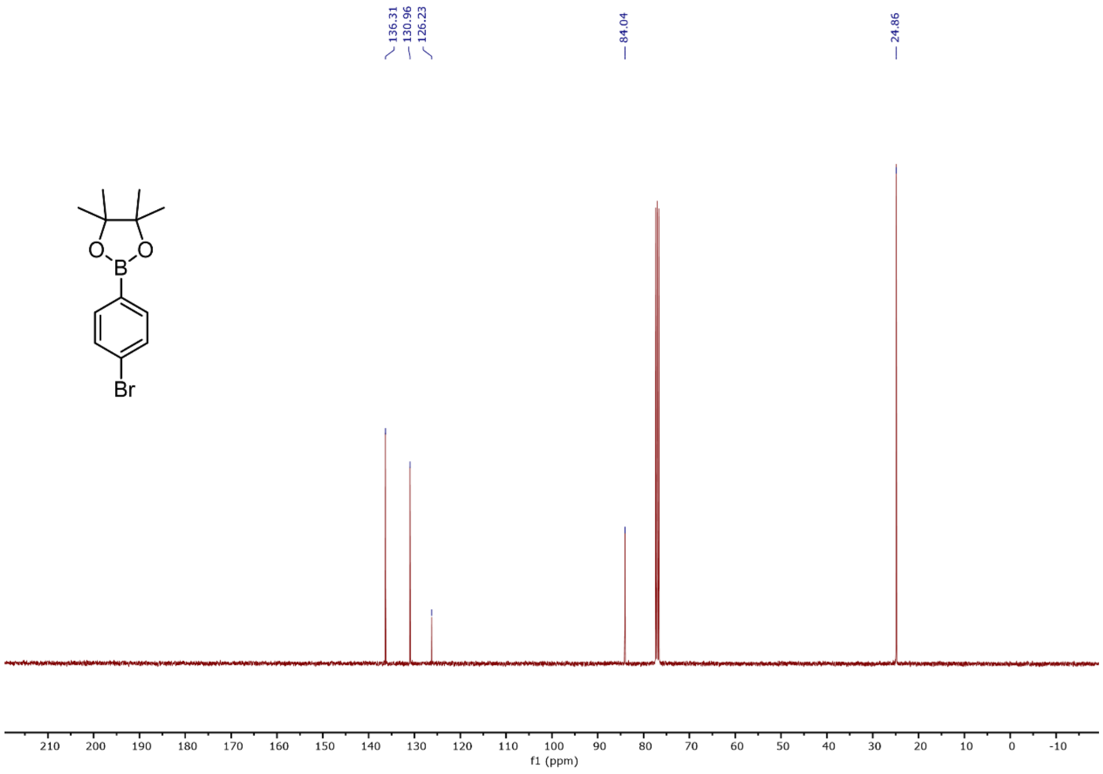


$^{13}$C NMR of *2-(4-bromophenyl)-4,4,5,5-tetramethyl-1,3,2-dioxaborolane* **(2u)**

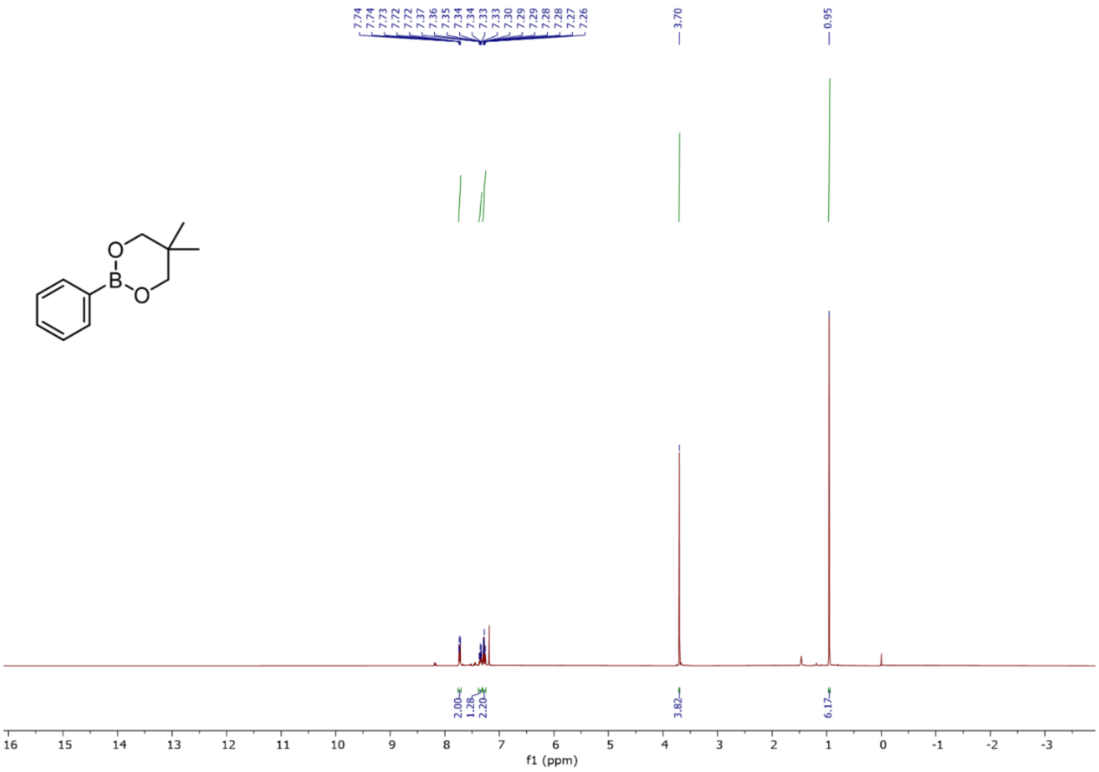

$^{1}$H NMR of *5,5-dimethyl-2-phenyl-1,3,2-dioxaborinane* **(3a)**

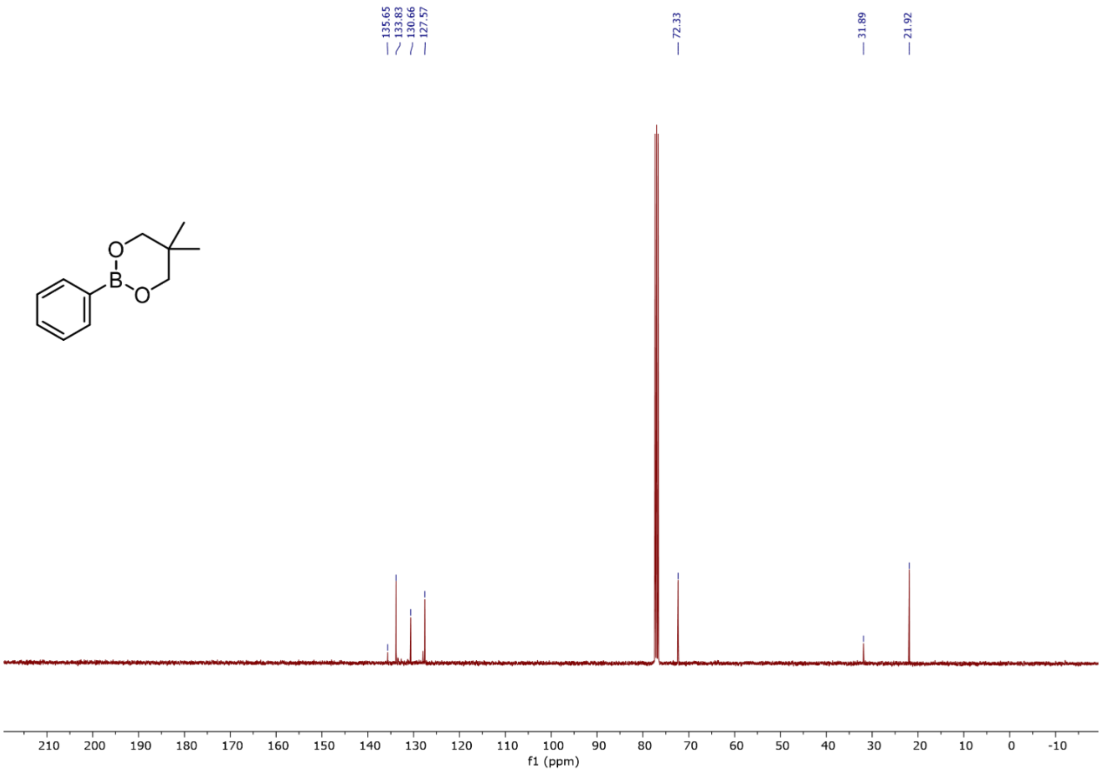

$^{13}$C NMR of *5,5-dimethyl-2-phenyl-1,3,2-dioxaborinane* **(3a)**

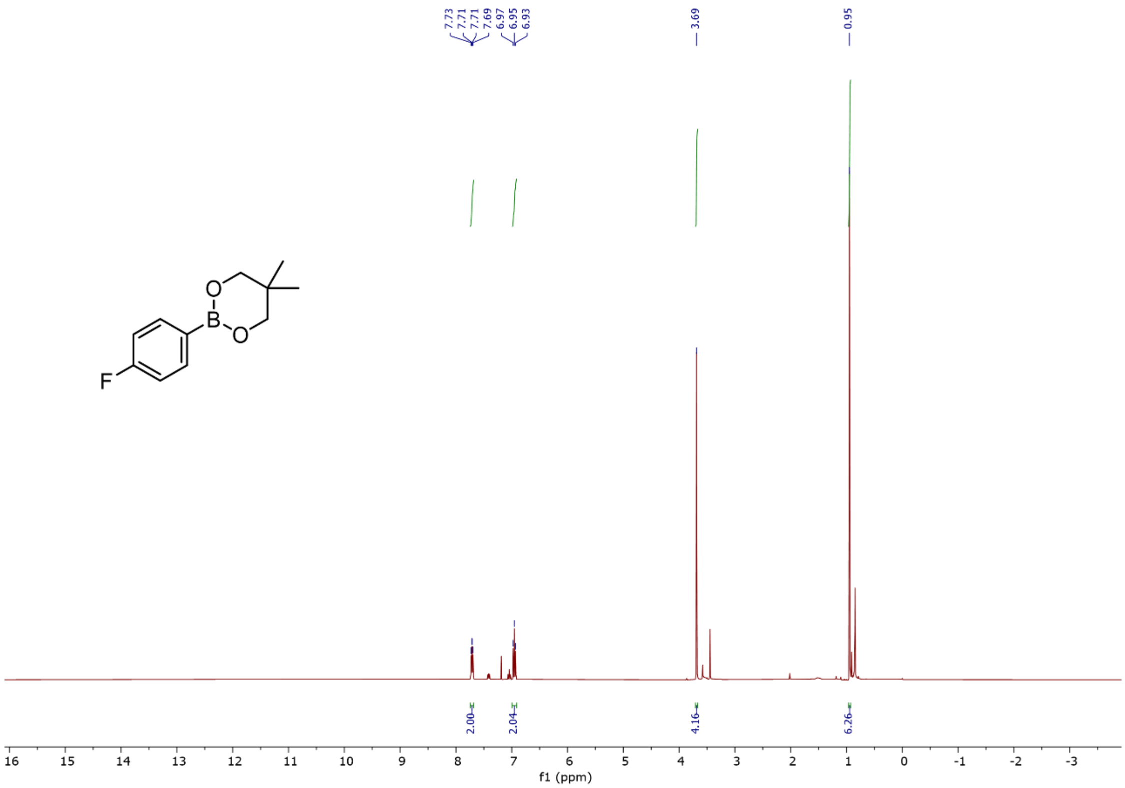


$^{1}$H NMR of *2-(4-fluorophenyl)-5,5-dimethyl-1,3,2-dioxaborinane* **(3b)**

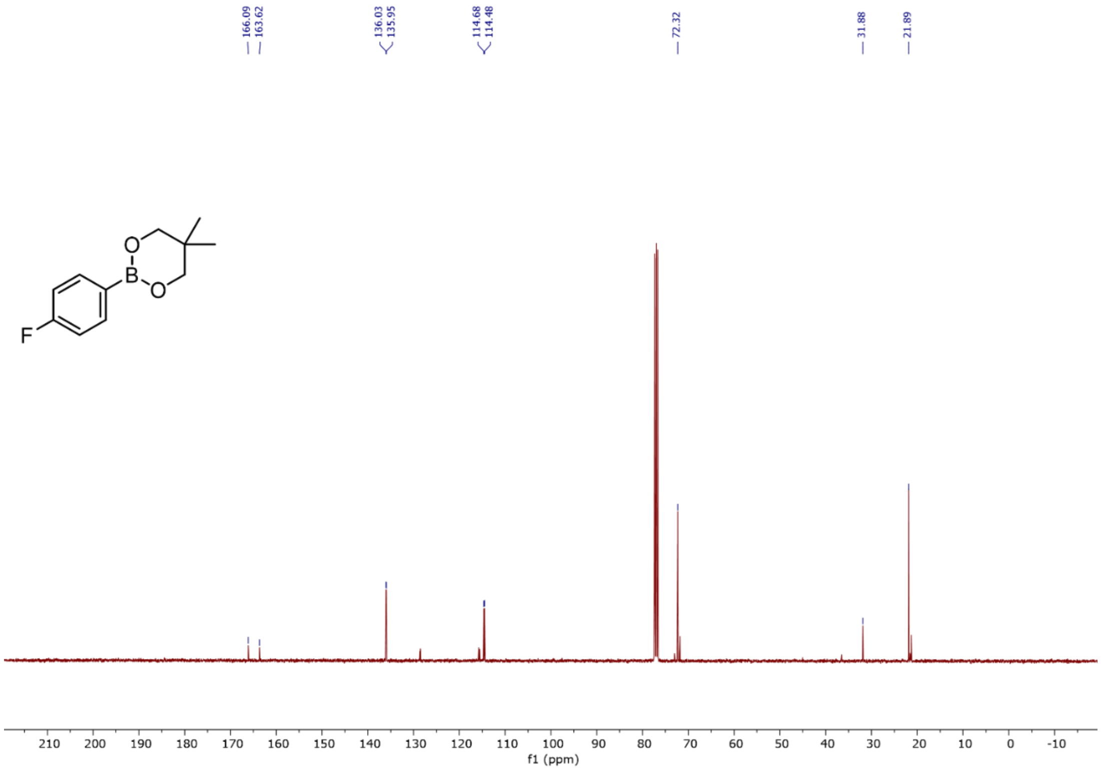


$^{13}$C NMR of *2-(4-fluorophenyl)-5,5-dimethyl-1,3,2-dioxaborinane* **(3b)**

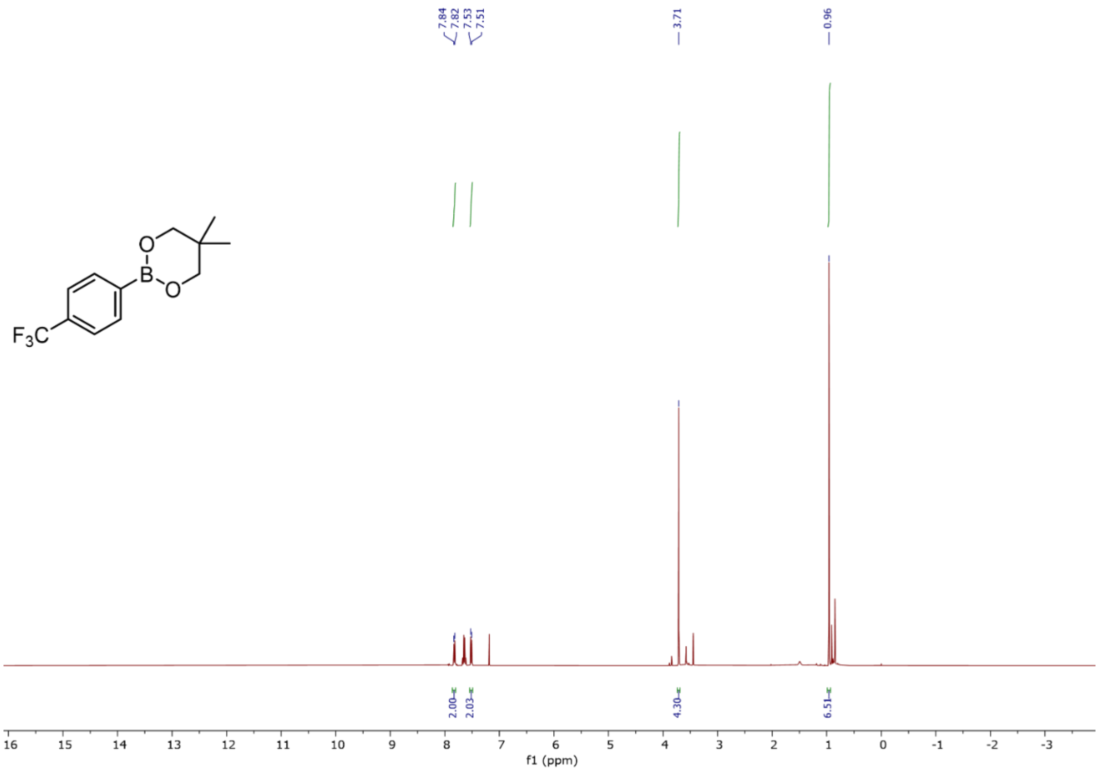


$^{1}$H NMR of *5,5-dimethyl-2-(4-(trifluoromethyl)phenyl)-1,3,2-dioxaborinane* **(3d)**

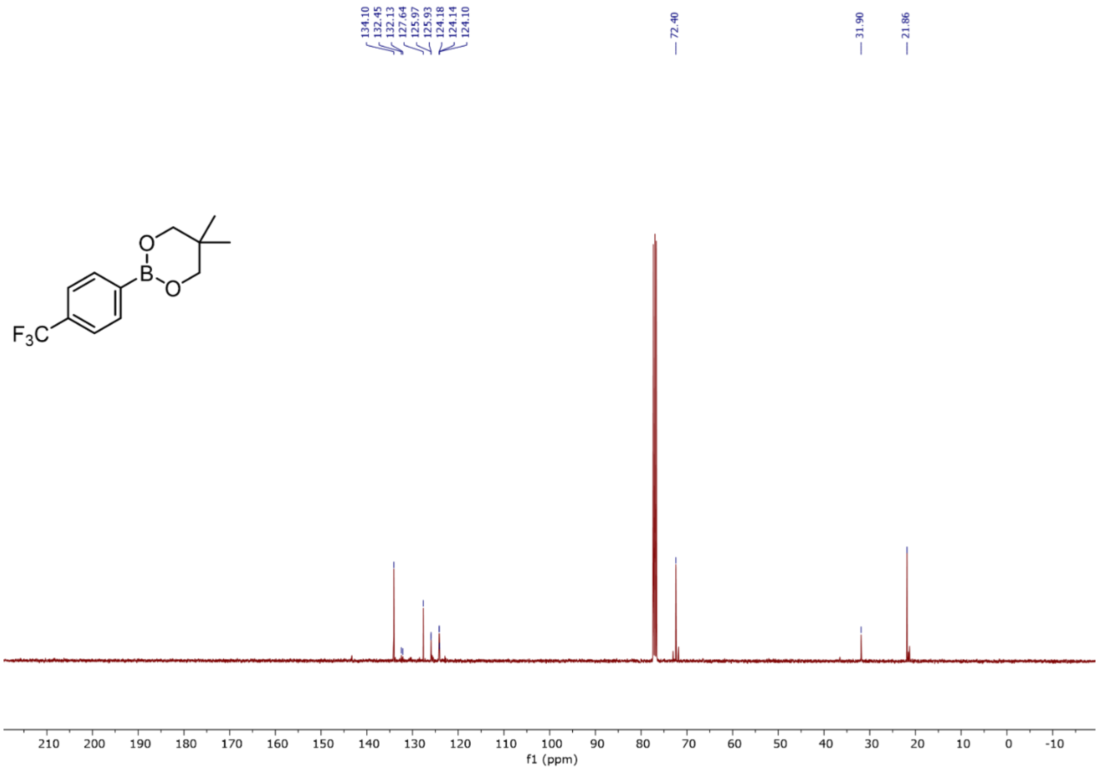


$^{13}$C NMR of *5,5-dimethyl-2-(4-(trifluoromethyl)phenyl)-1,3,2-dioxaborinane* **(3d)**

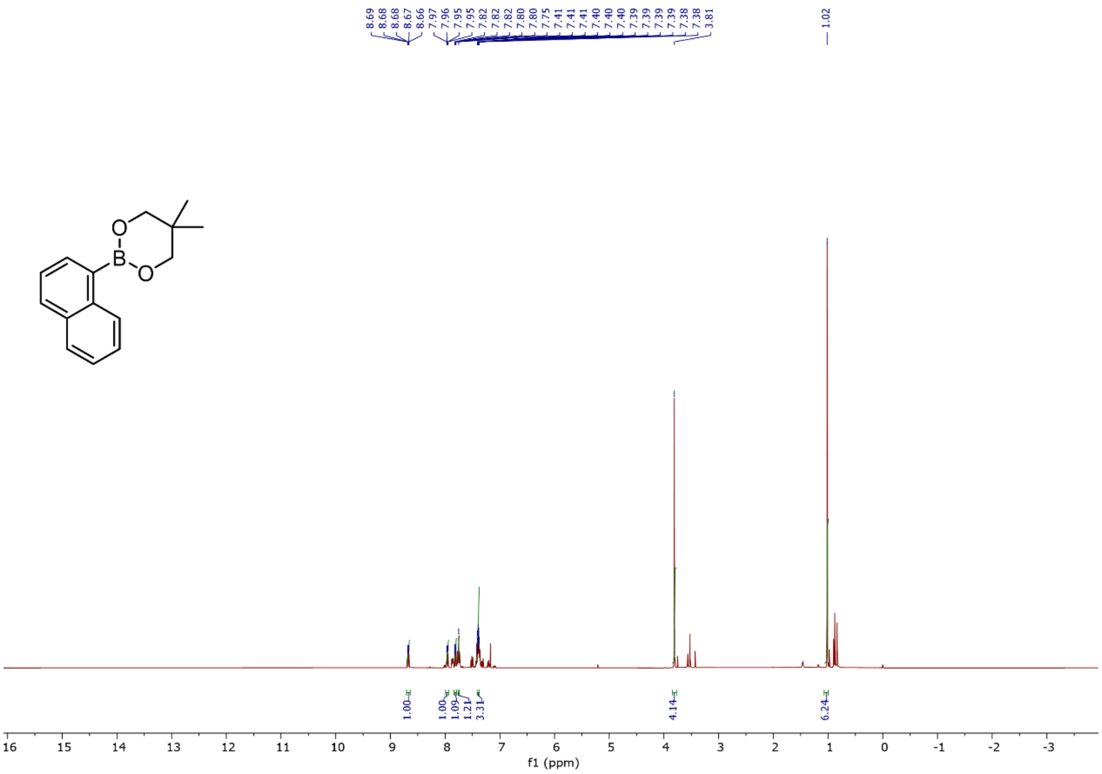


$^{1}$H NMR of *5,5-dimethyl-2-(naphthalen-1-yl)-1,3,2-dioxaborinane* **(3e).**

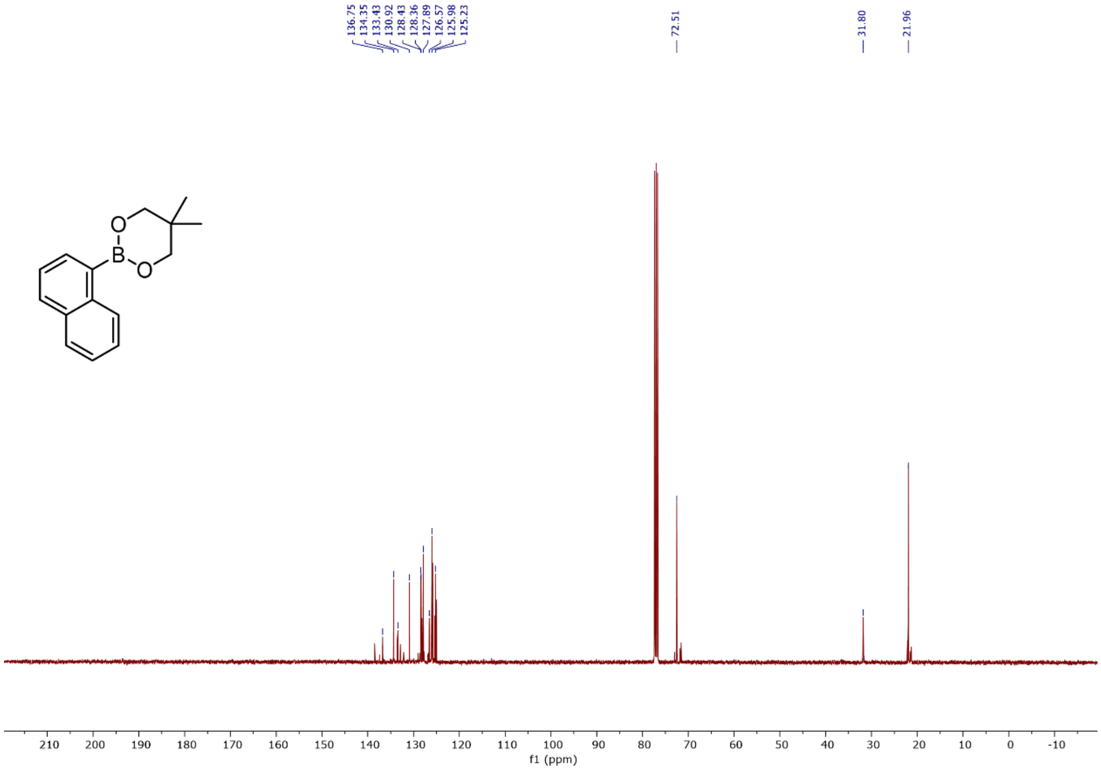


$^{13}$C NMR of *5,5-dimethyl-2-(naphthalen-1-yl)-1,3,2-dioxaborinane* **(3e).**

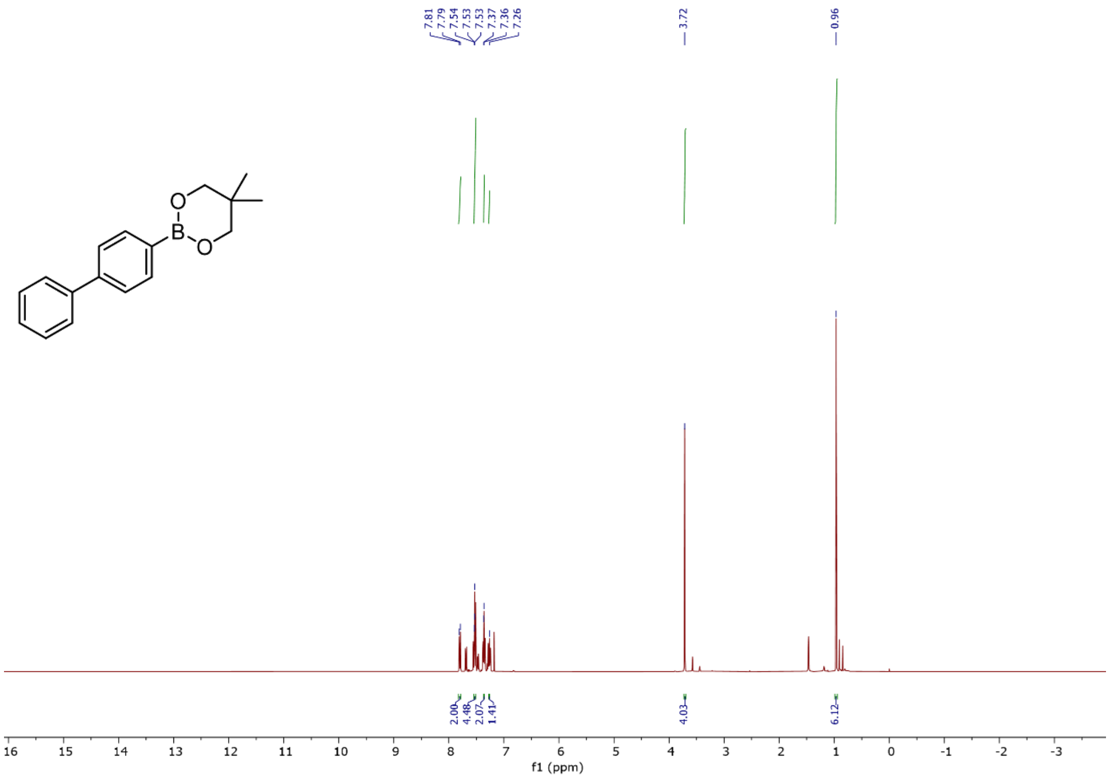


$^{1}$H NMR of *2-([1,1'-biphenyl]-4-yl)-5,5-dimethyl-1,3,2-dioxaborinane* **(3f)**

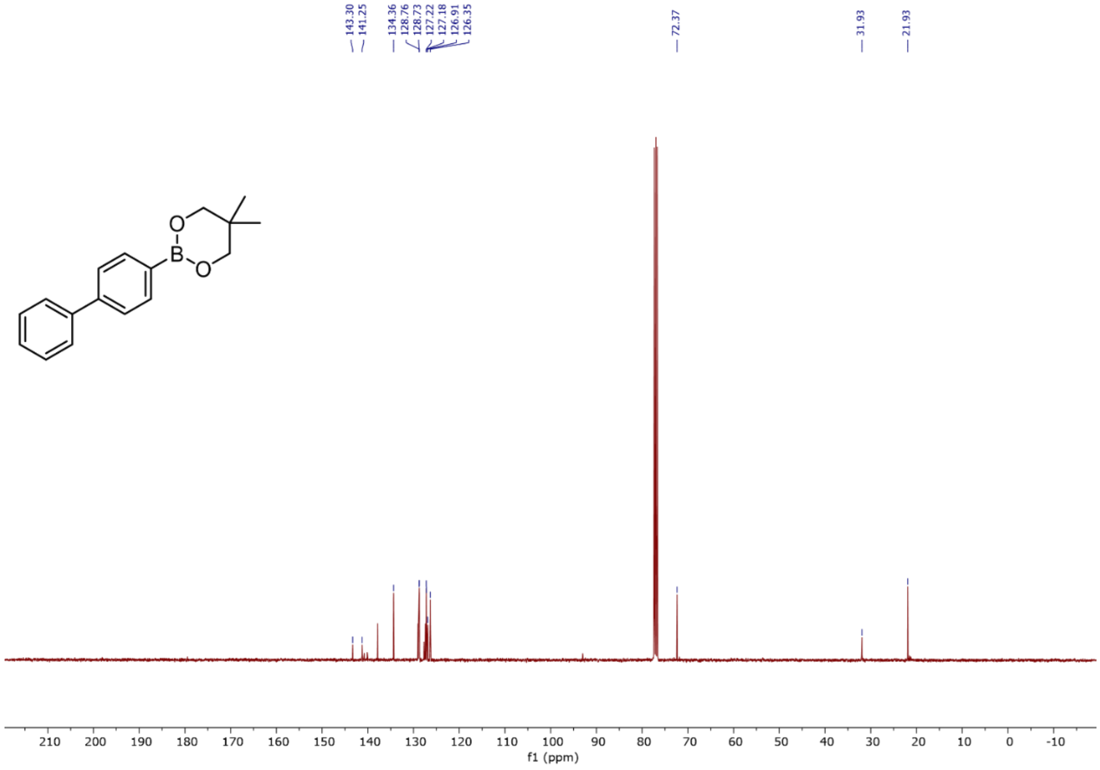


$^{13}$C NMR of *2-([1,1'-biphenyl]-4-yl)-5,5-dimethyl-1,3,2-dioxaborinane* **(3f)**

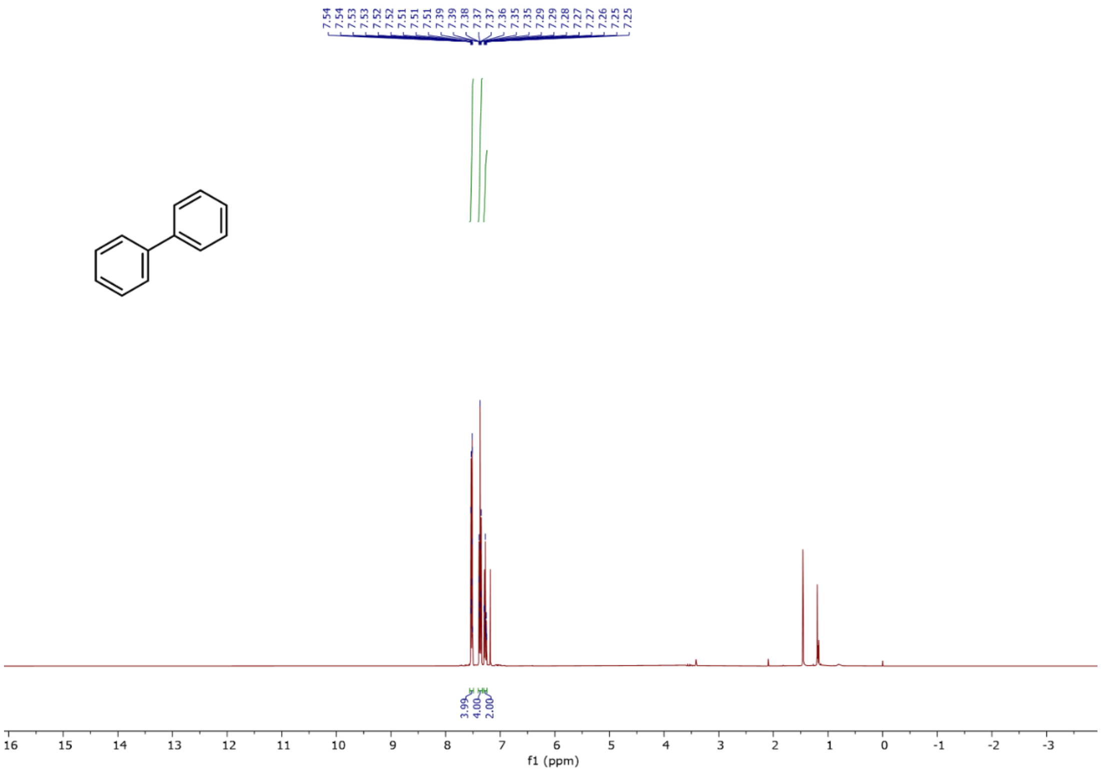

$^{1}H$ NMR of *1,1'-biphenyl* **(4a)**

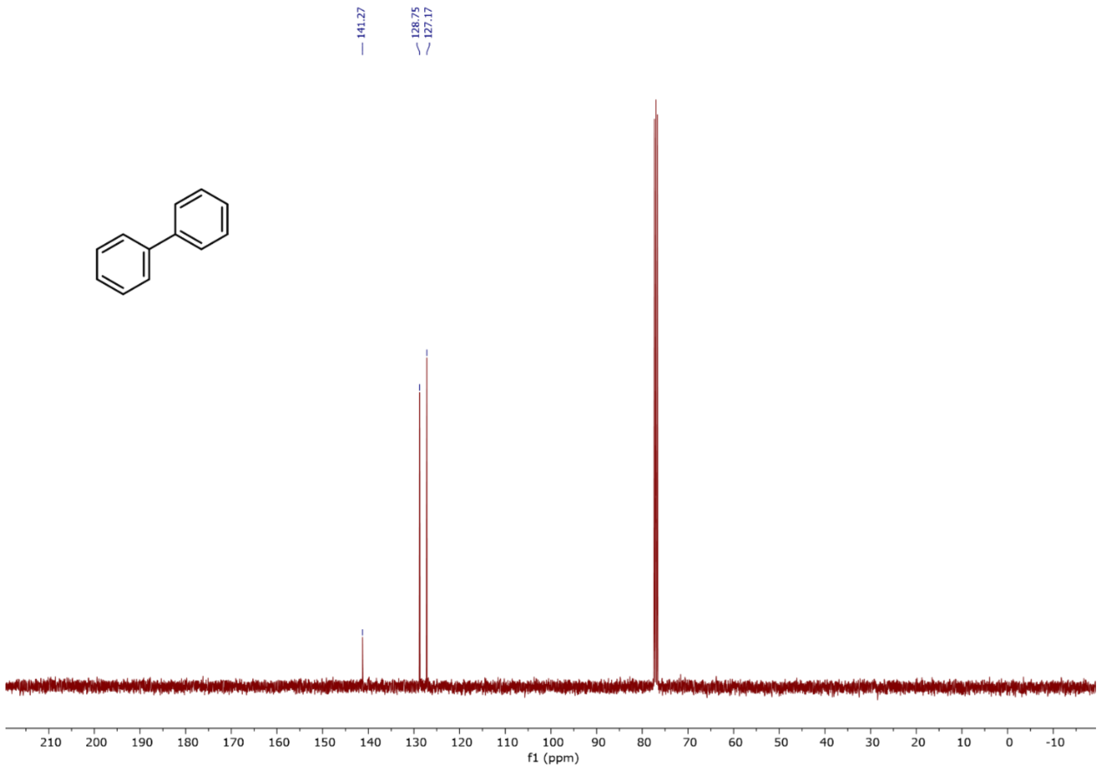

$^{13}C$ NMR of *1,1'-biphenyl* **(4a)**

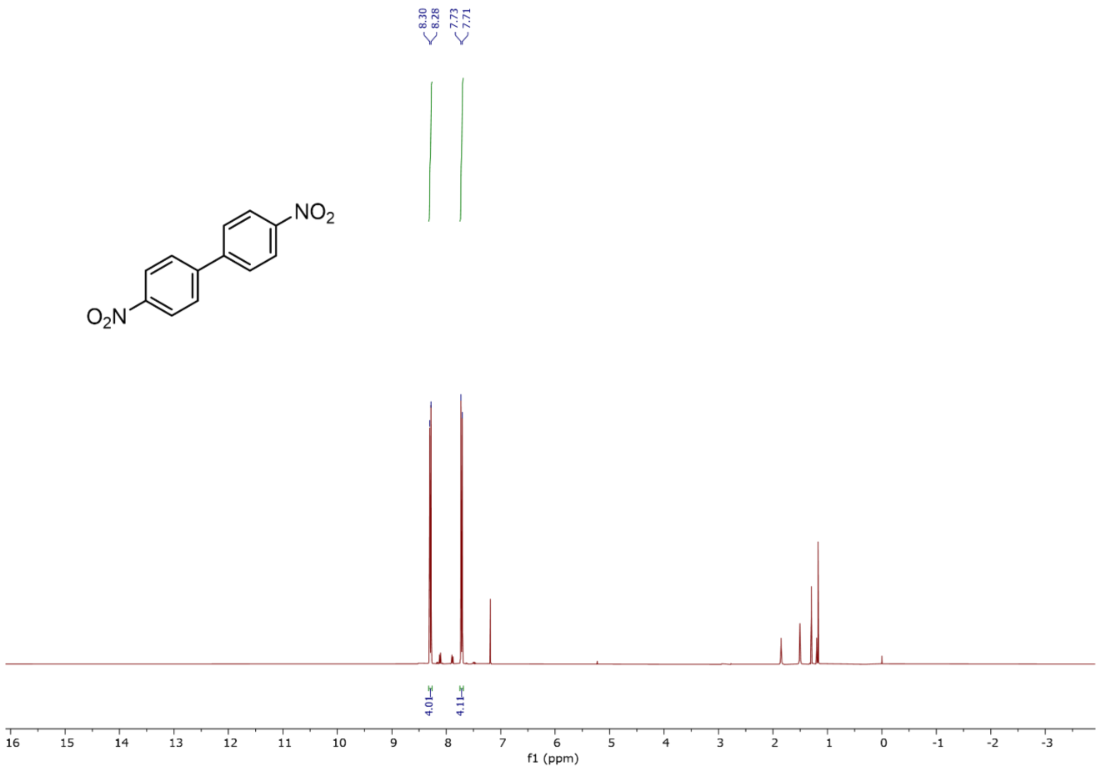


$^{1}$H NMR of *4,4'-dinitro-1,1'-biphenyl* **(4b)**

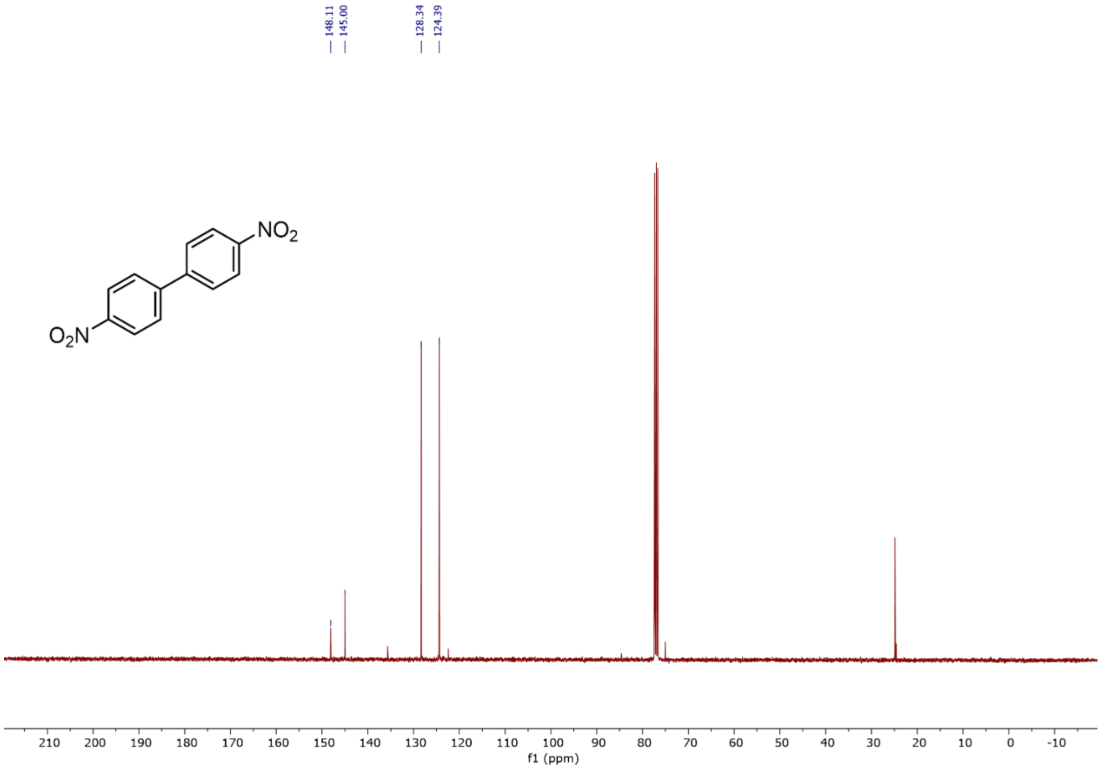


$^{13}$C NMR of *4,4'-dinitro-1,1'-biphenyl* **(4b)**

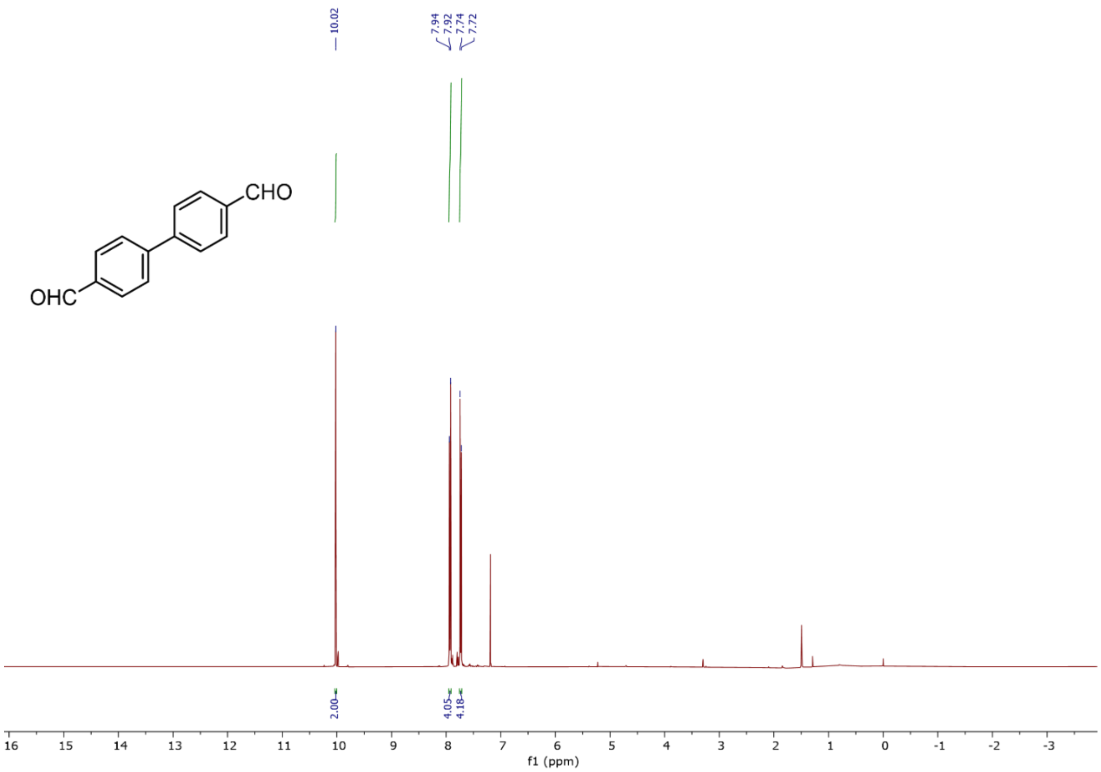


$^{1}$H NMR of *[1,1'-biphenyl]-4,4'-dicarbaldehyde* **(4c)**

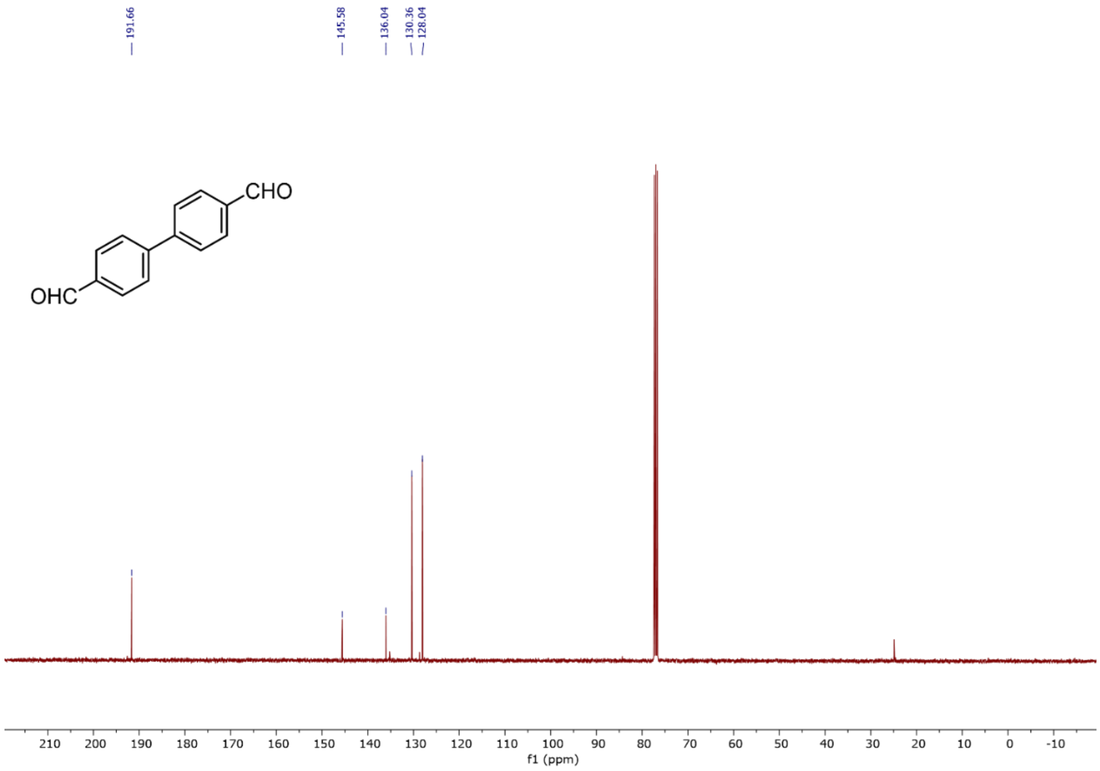


$^{13}$C NMR of *[1,1'-biphenyl]-4,4'-dicarbaldehyde* **(4c)**

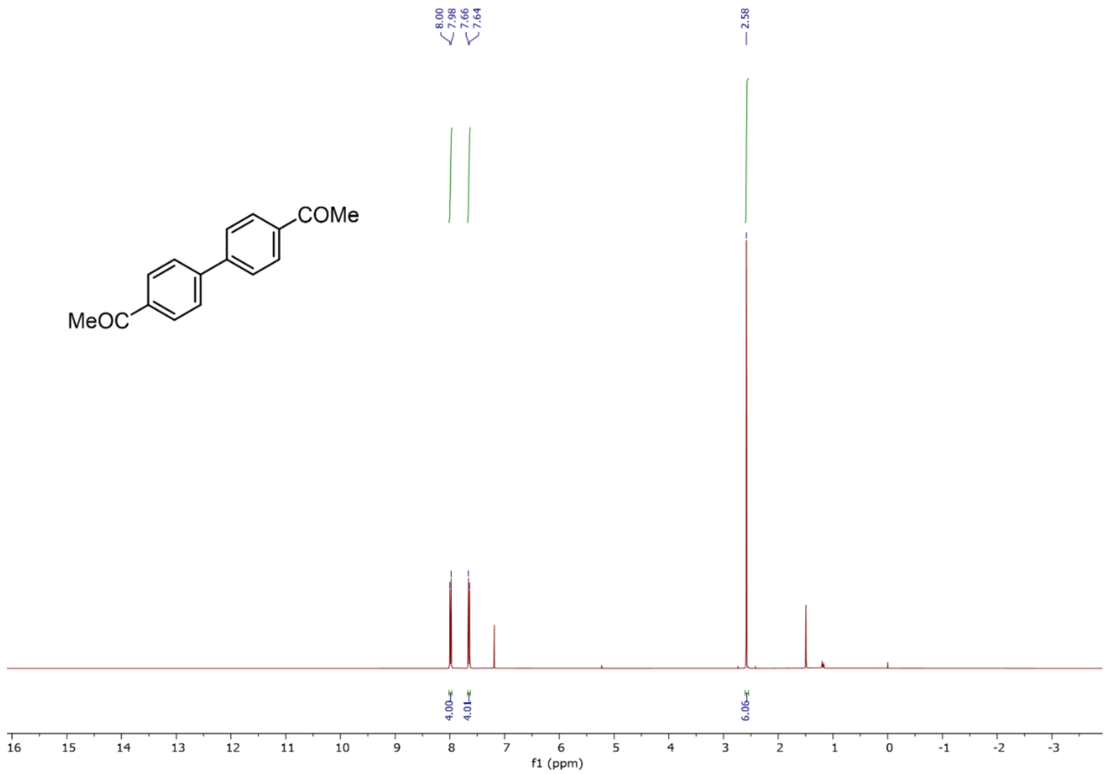


$^{1}$H NMR of *1,1'-([1,1'-biphenyl]-4,4'-diyl)bis(ethan-1-one)* **(4d)**

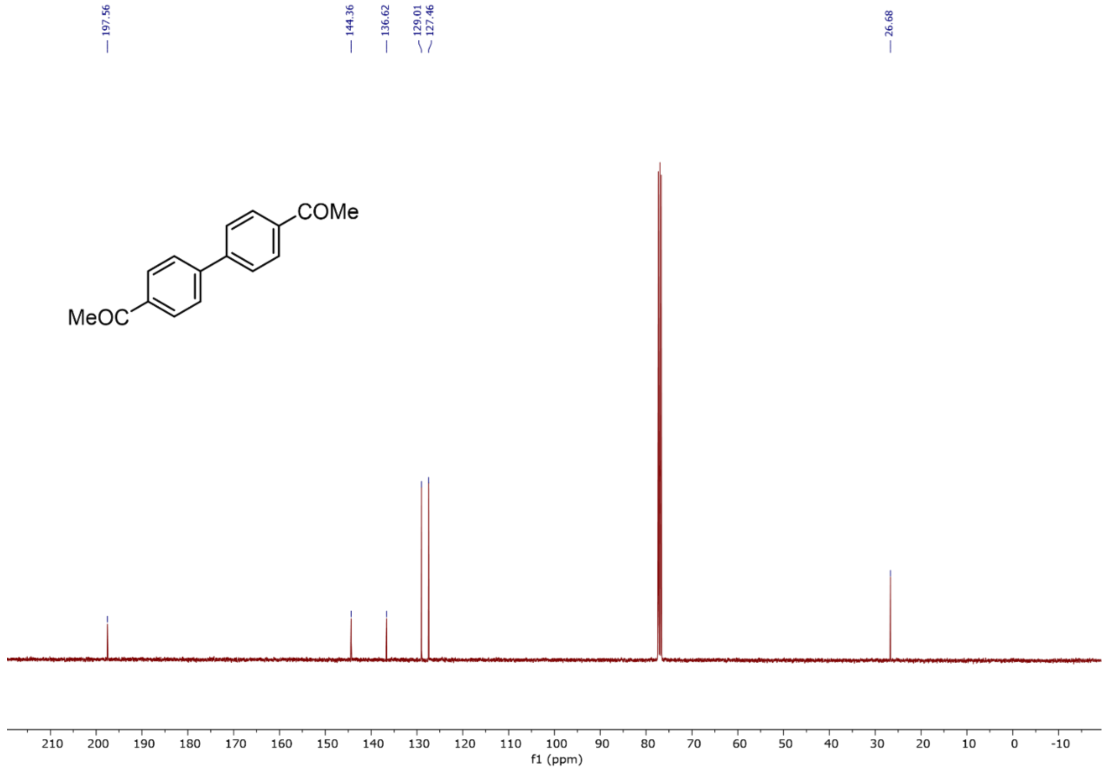


$^{13}$C NMR of *1,1'-([1,1'-biphenyl]-4,4'-diyl)bis(ethan-1-one)* **(4d).**

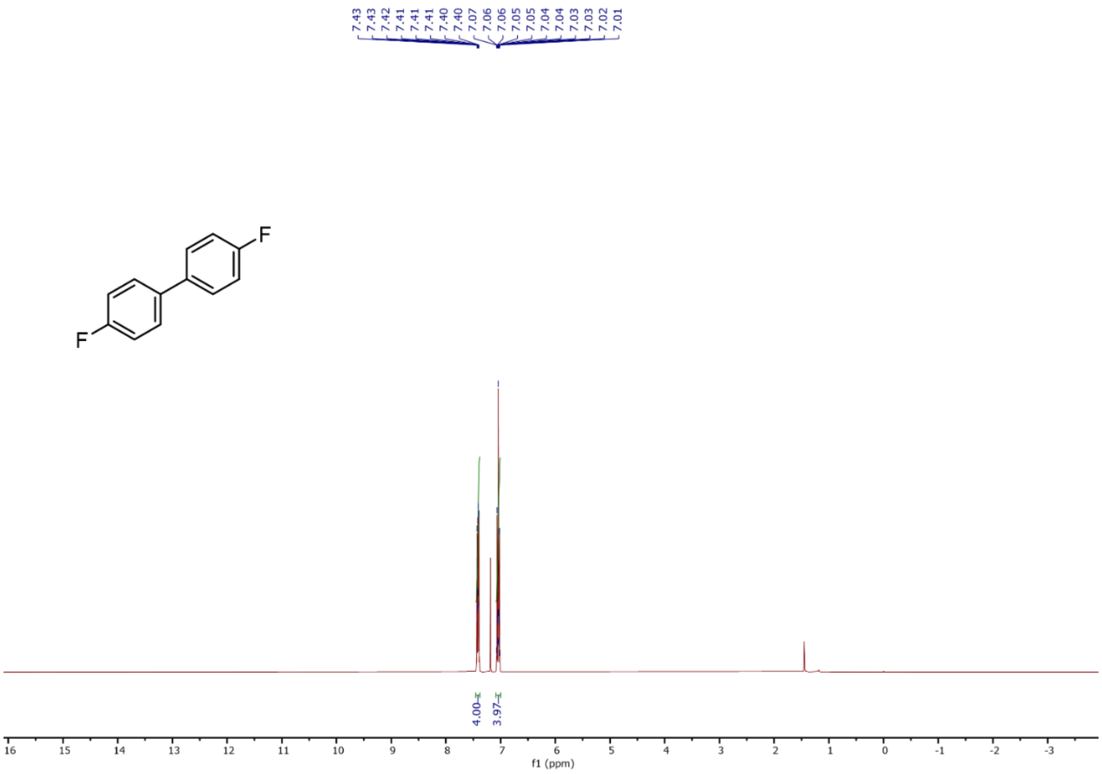


$^{1}$H NMR of *4,4'-difluoro-1,1'-biphenyl* (**4f**)

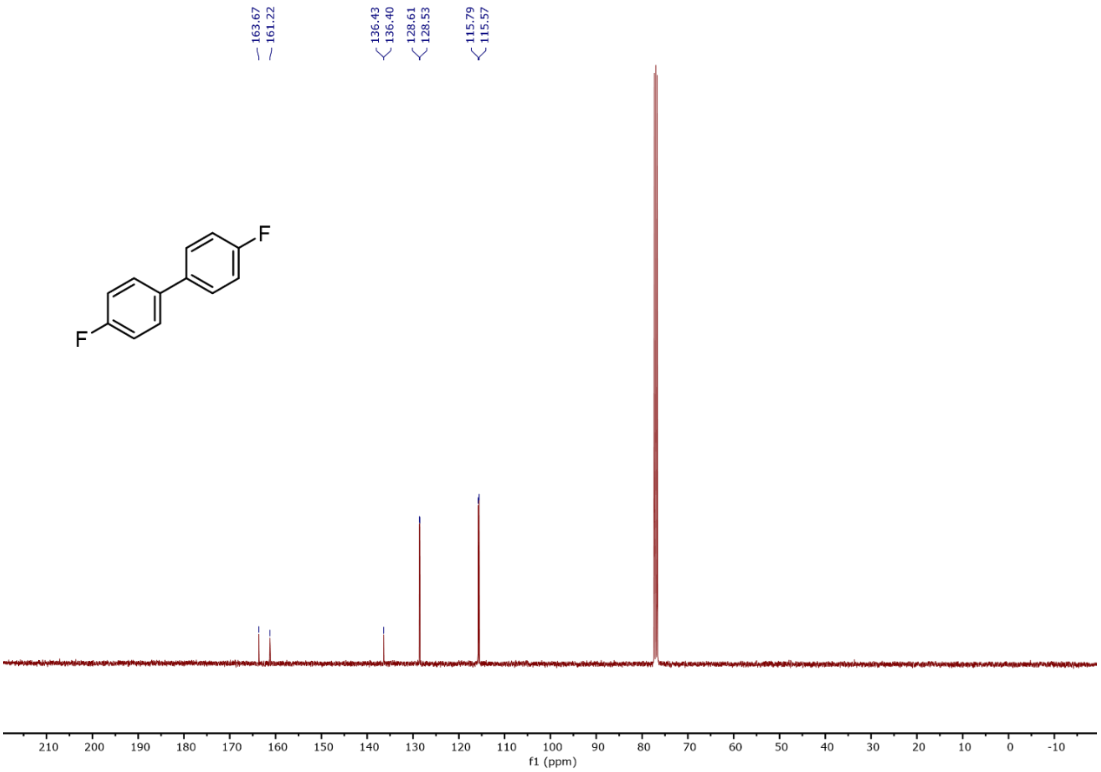


$^{13}$C NMR of *4,4'-difluoro-1,1'-biphenyl* (**4f**)

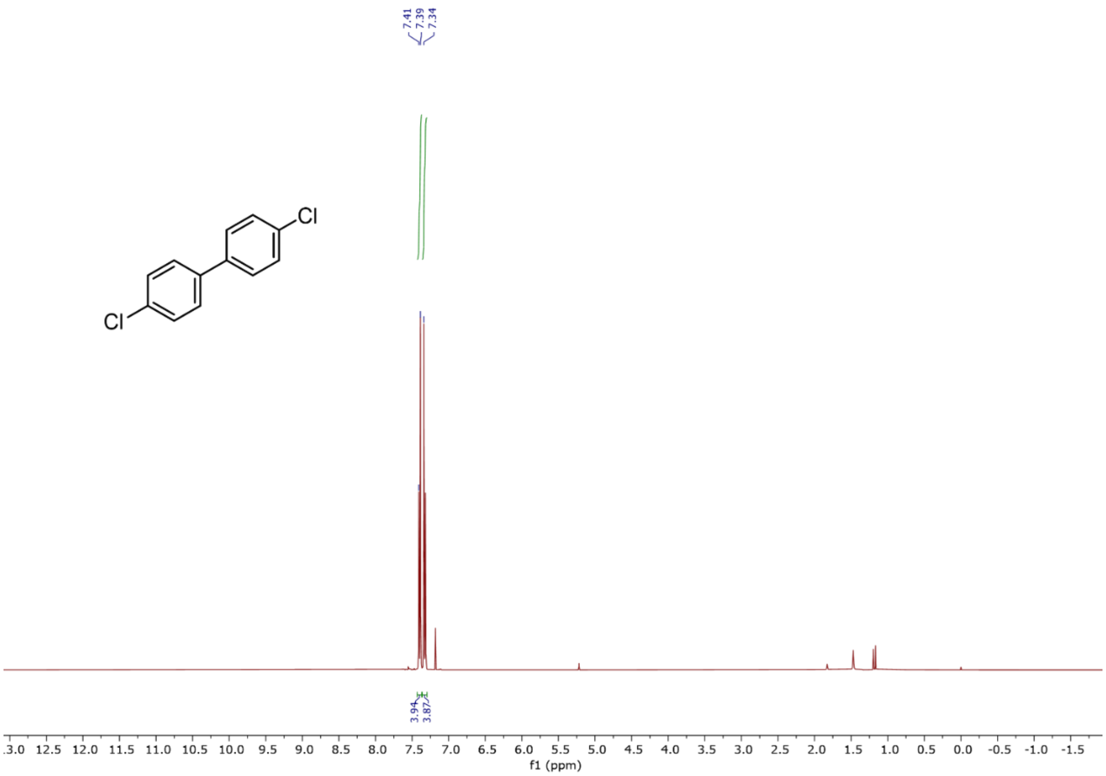

$^{1}$H NMR of *4,4'-dichloro-1,1'-biphenyl* **(4g)**

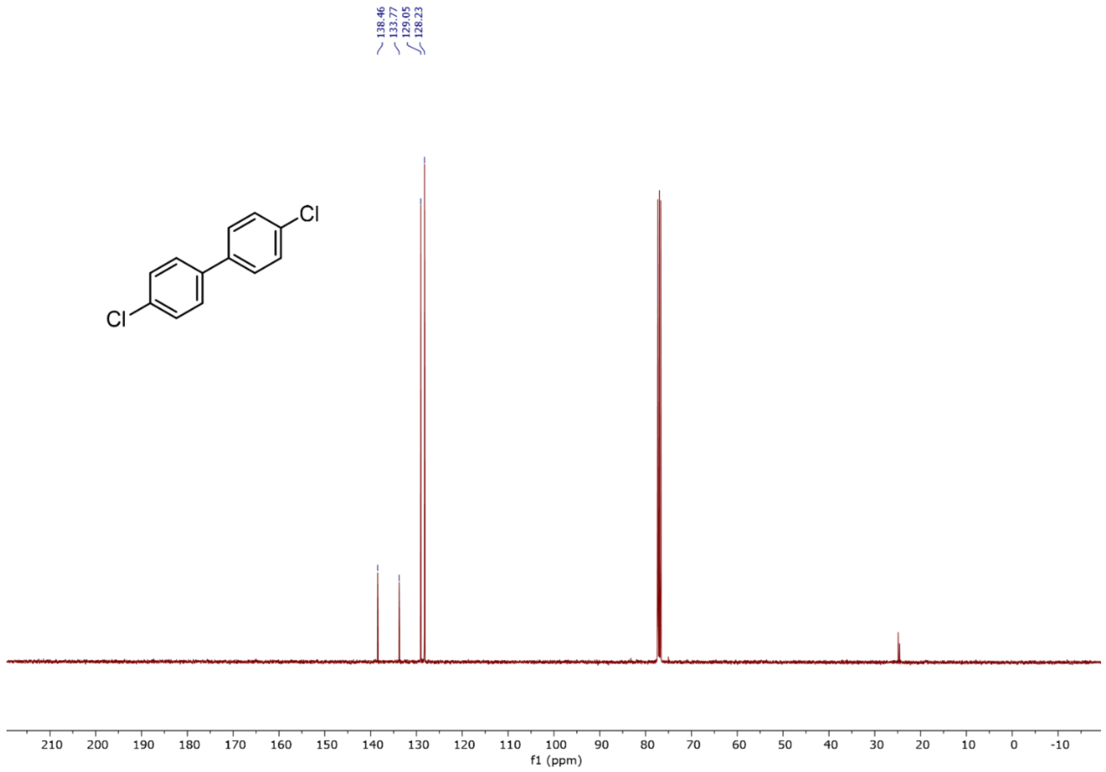

$^{13}$C NMR of *4,4'-dichloro-1,1'-biphenyl* **(4g)**

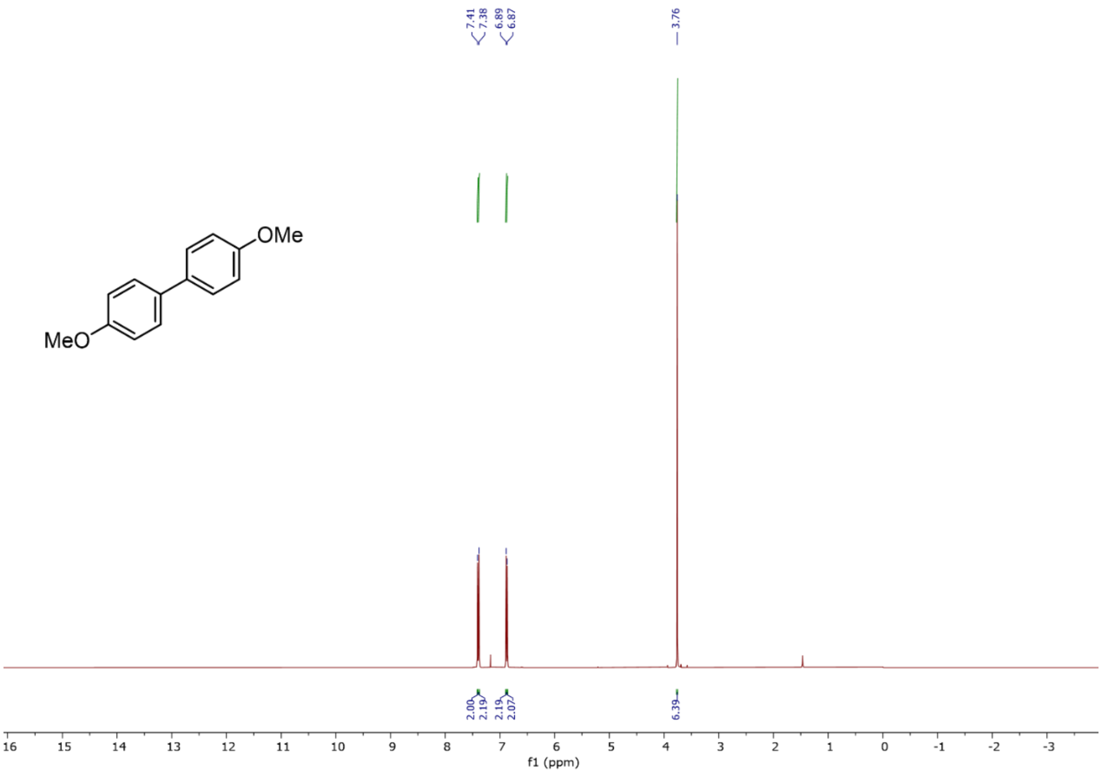


$^1$H NMR of *4,4'-dimethoxy-1,1'-biphenyl* **(4k)**

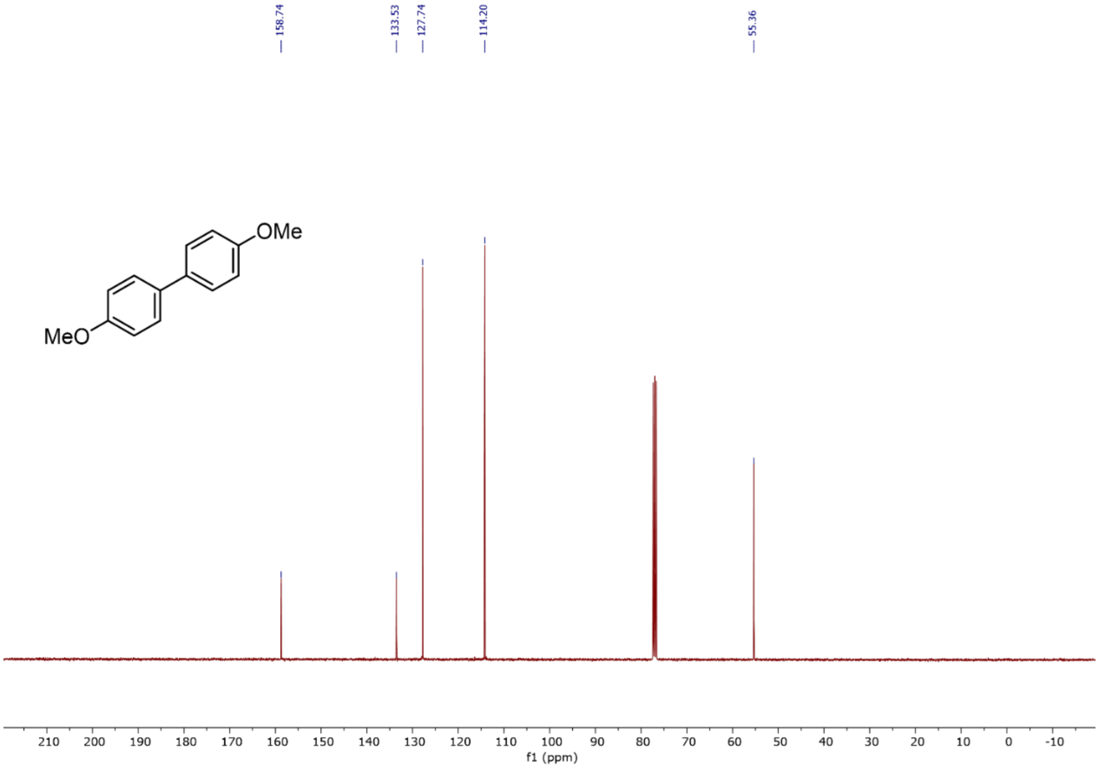


$^{13}$C NMR of *4,4'-dimethoxy-1,1'-biphenyl* **(4k)**

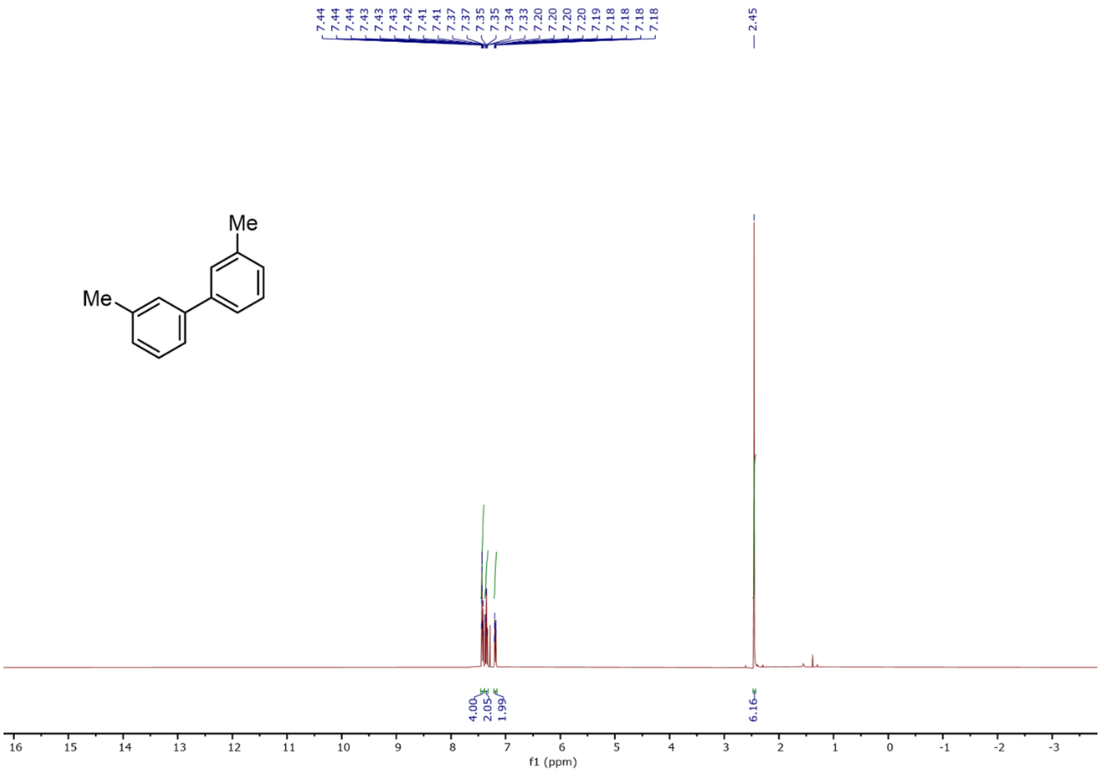


$^{1}$H NMR of *3,3'-dimethyl-1,1'-biphenyl* **(4l)**

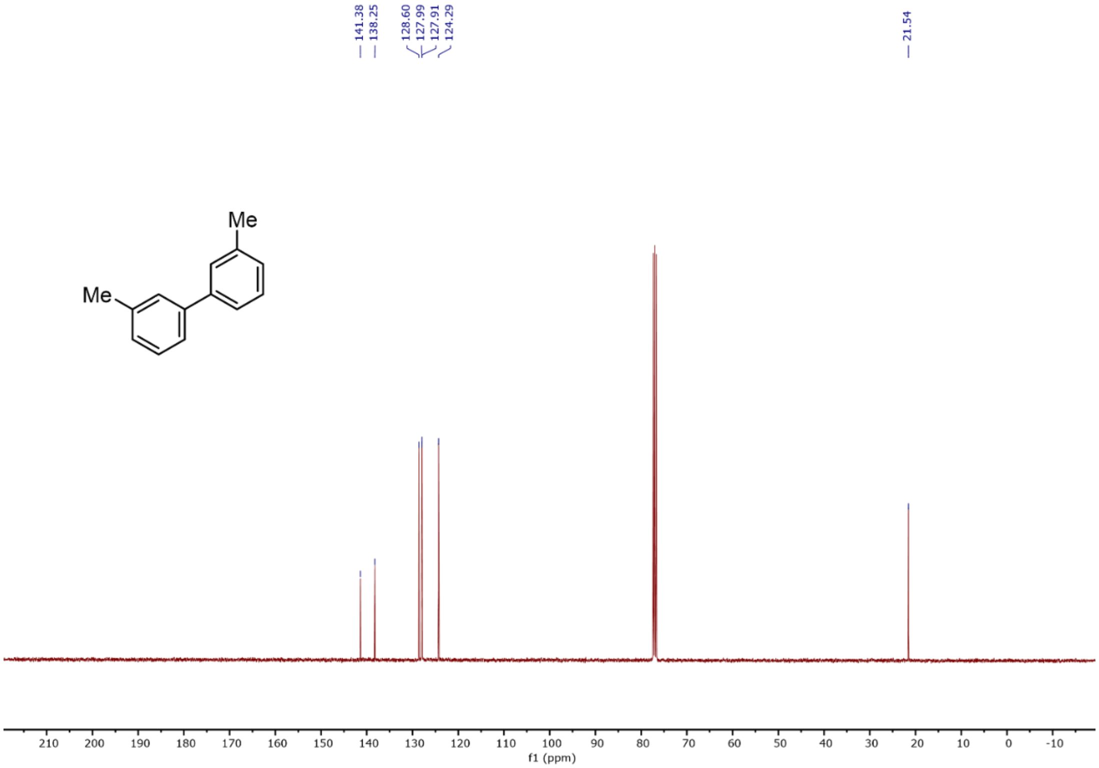


$^{13}$C NMR of *3,3'-dimethyl-1,1'-biphenyl* **(4l)**

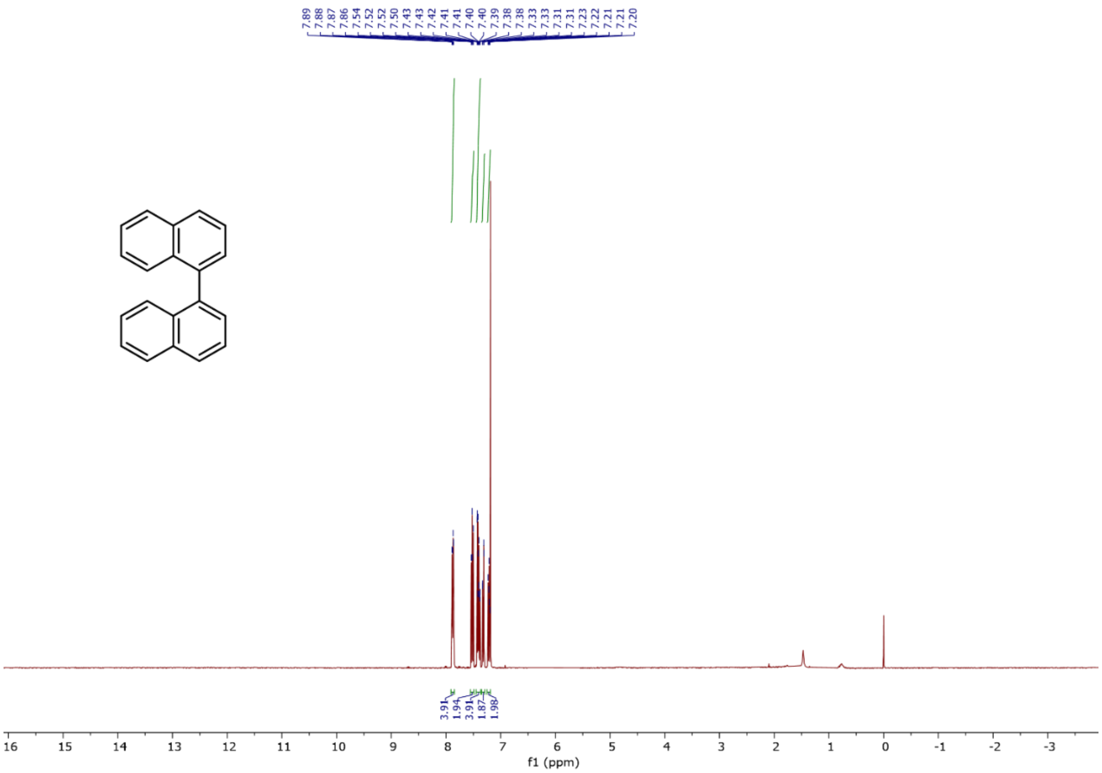

$^{1}$H NMR of *1,1'-binaphthalene* **(4n).**

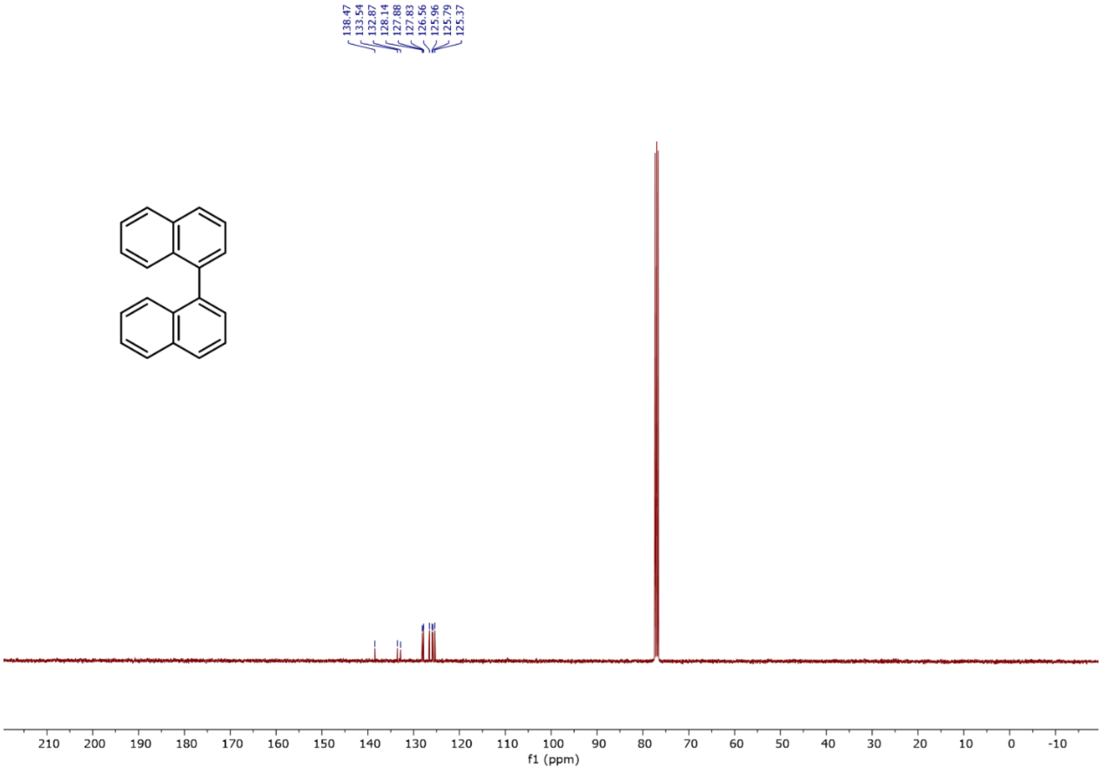

$^{13}$C NMR of *1,1'-binaphthalene* **(4n).**

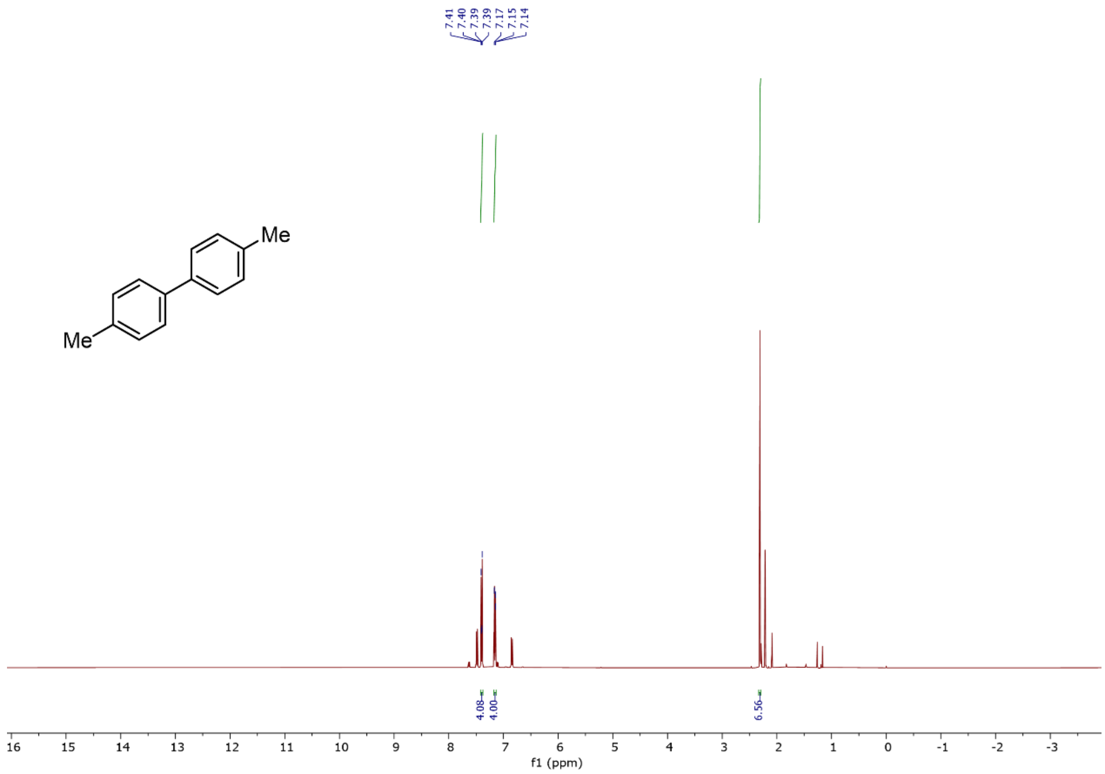

$^{1}$H NMR of *4,4'-dimethyl-1,1'-biphenyl* **(4i)**

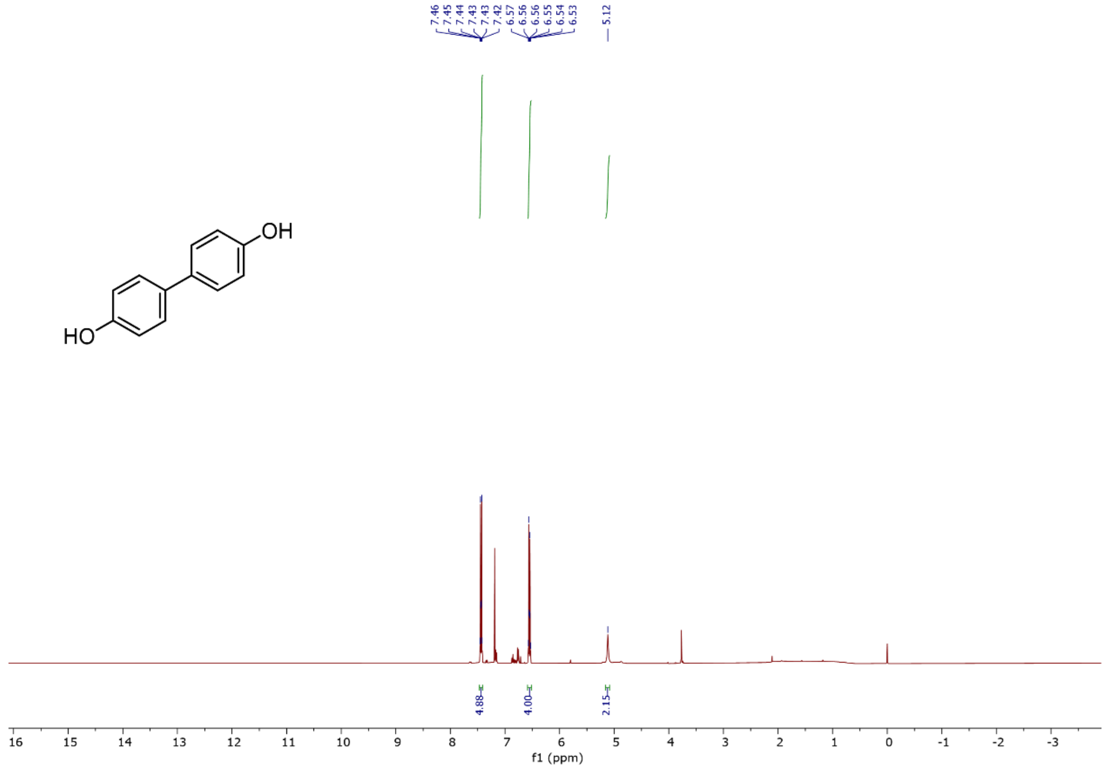

$^{1}$H NMR of *[1,1'-biphenyl]-4,4'-diol* **(4j).**

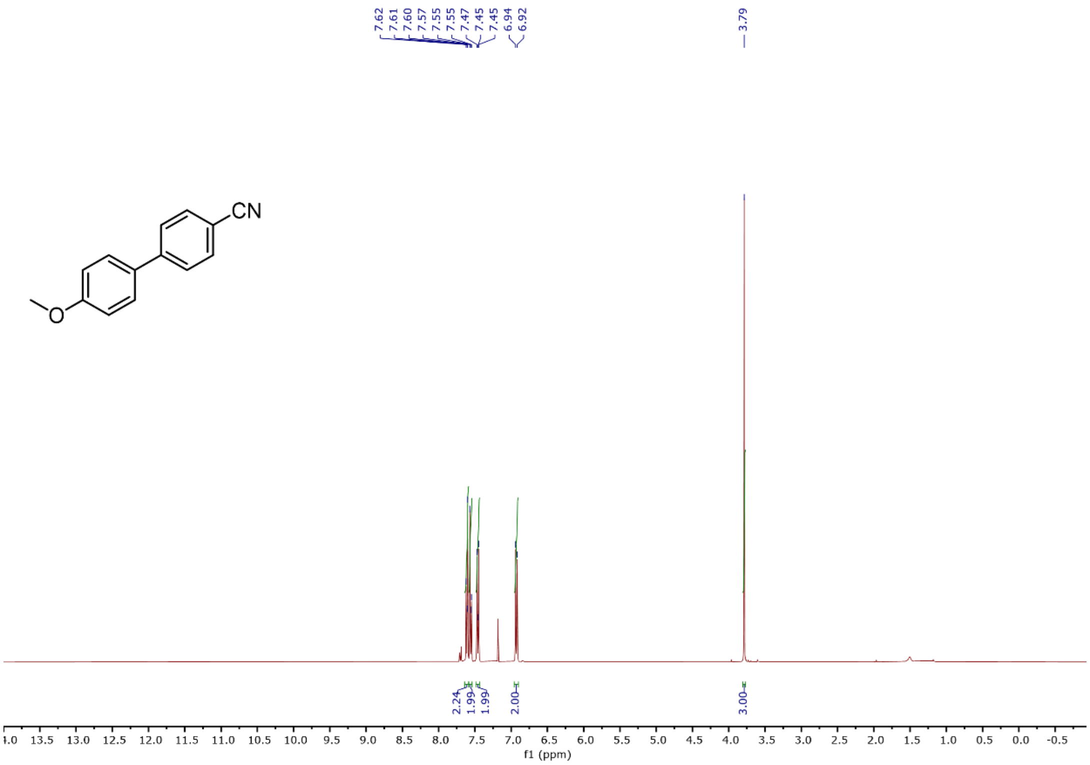


$^{1}$H NMR of *4-chloro-4'-methoxy-1,1'-biphenyl* **(4r)**

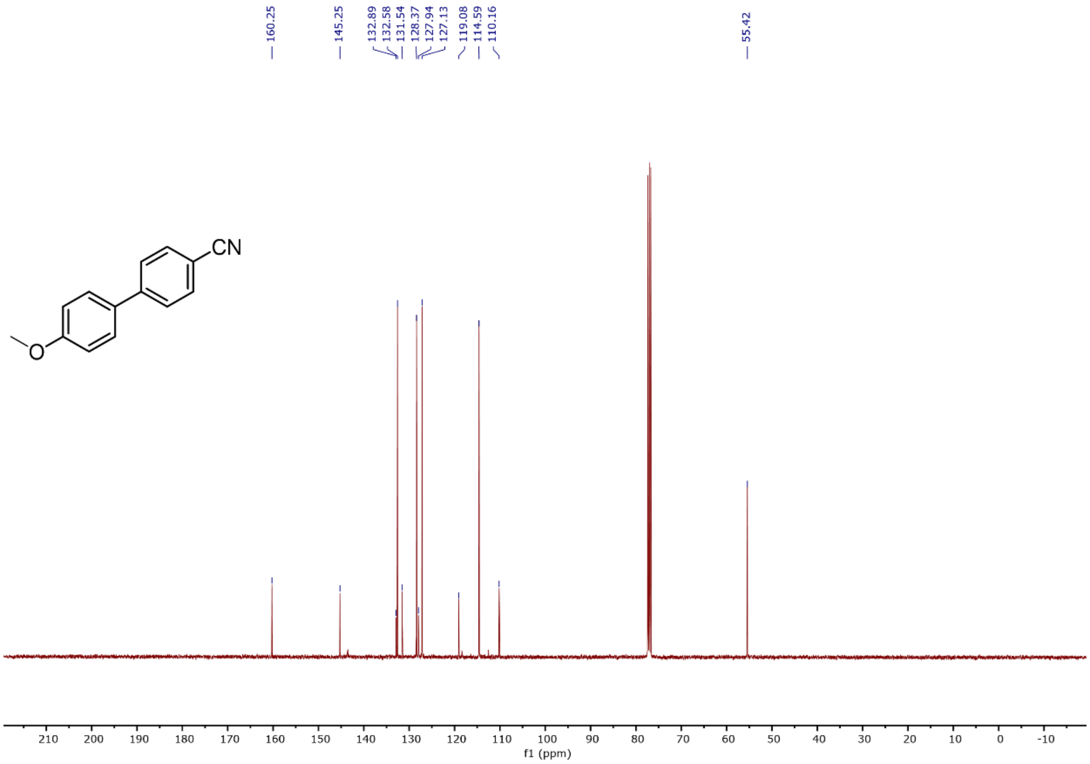


$^{13}$C NMR of *4-chloro-4'-methoxy-1,1'-biphenyl* **(4r)**

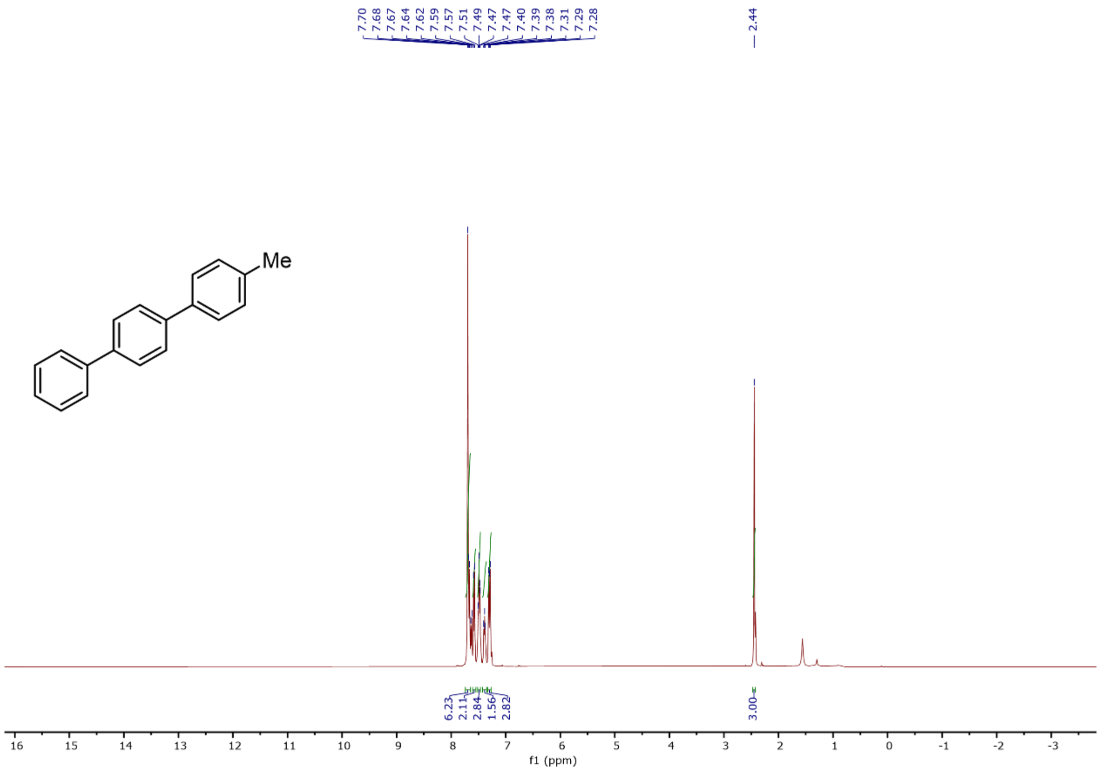


$^{1}$H NMR of *4-methyl-1,1':4',1''-terphenyl* **(4q)**

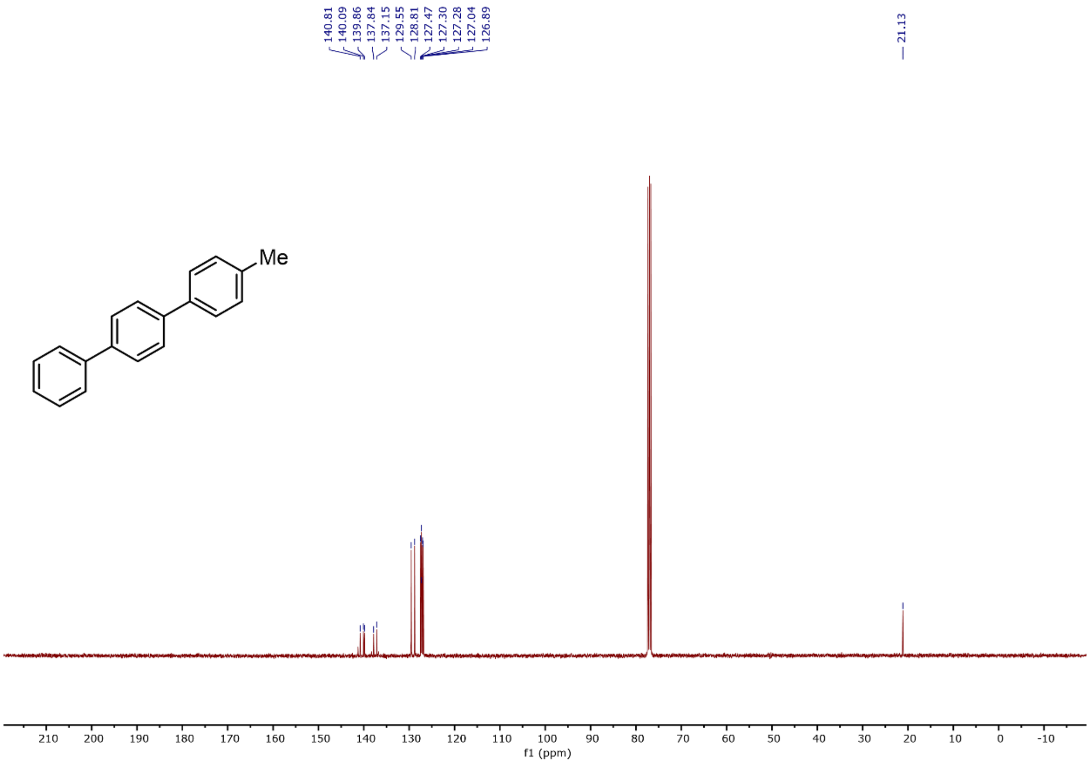


$^{13}$C NMR of *4-methyl-1,1':4',1''-terphenyl* **(4q)**

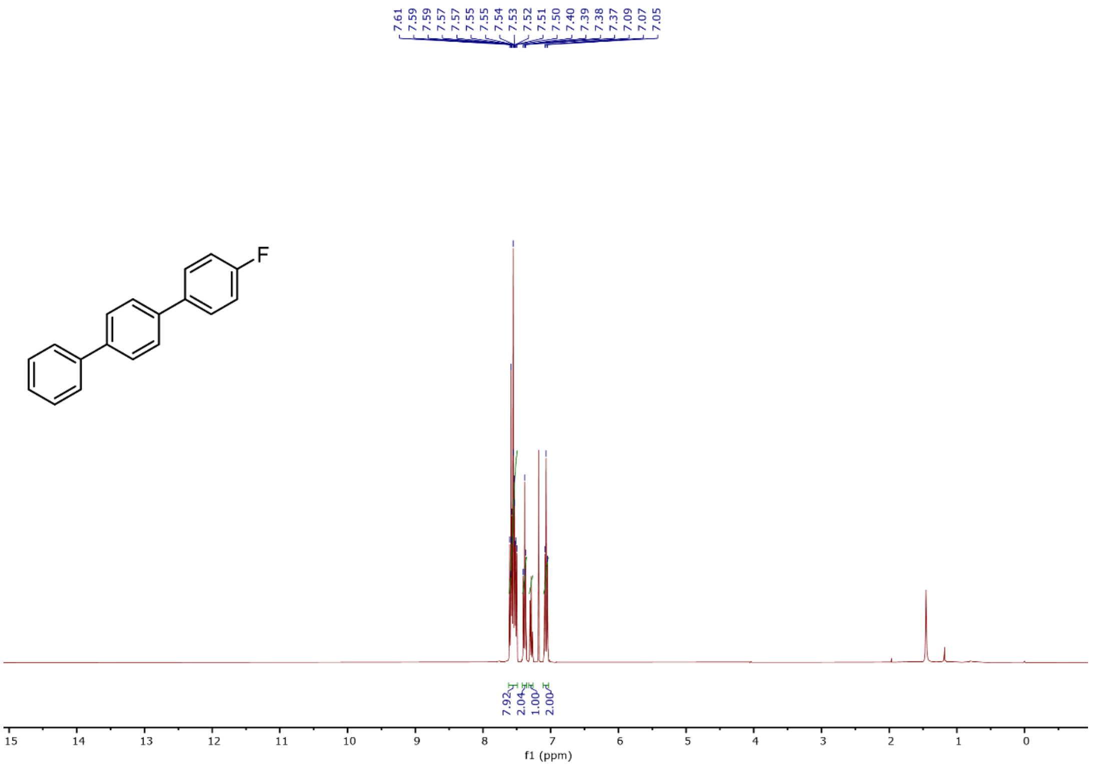


$^{1}$H NMR of *4-fluoro-1,1':4',1''-terphenyl* **(4s)**

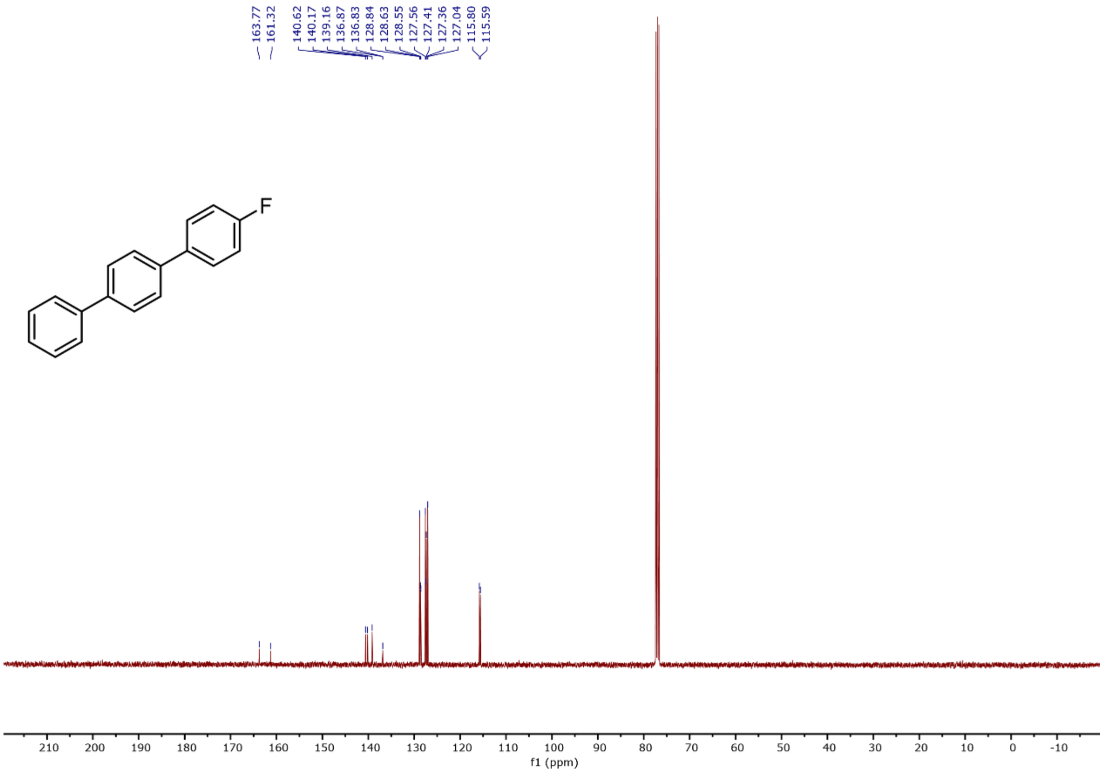


$^{13}$C NMR of *4-fluoro-1,1':4',1''-terphenyl* **(4s)**

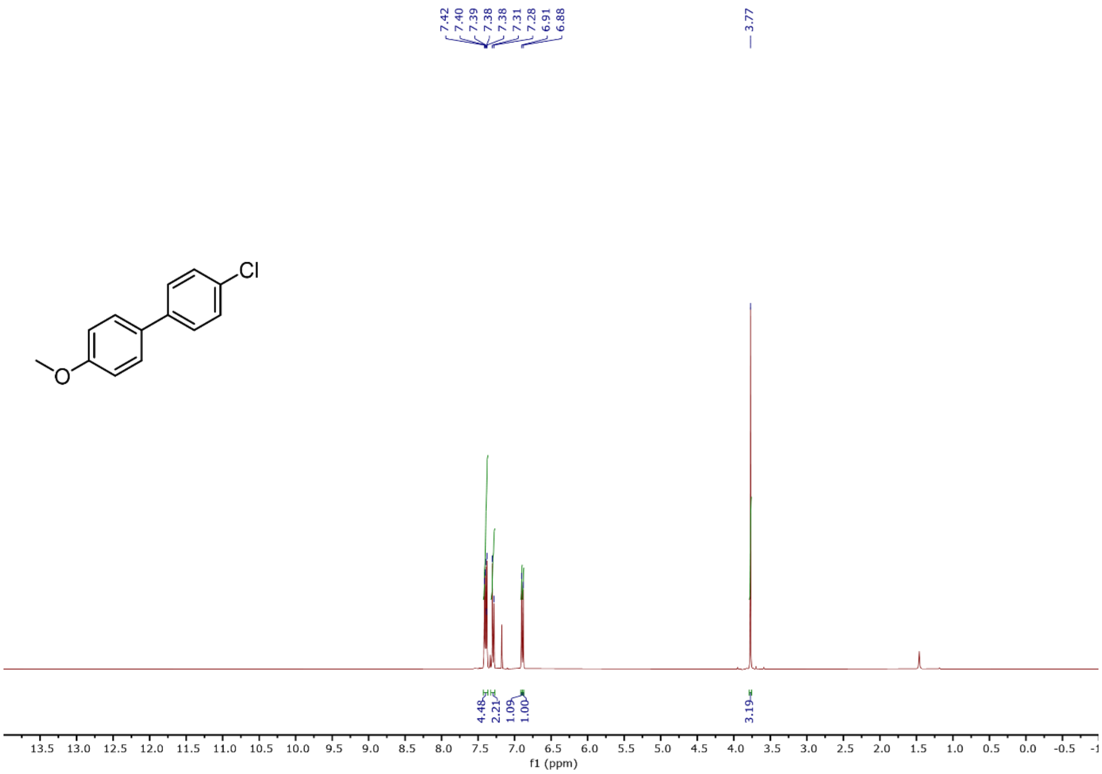


$^{1}$H NMR of *4-chloro-4'-methoxy-1,1'-biphenyl* **(4r)**

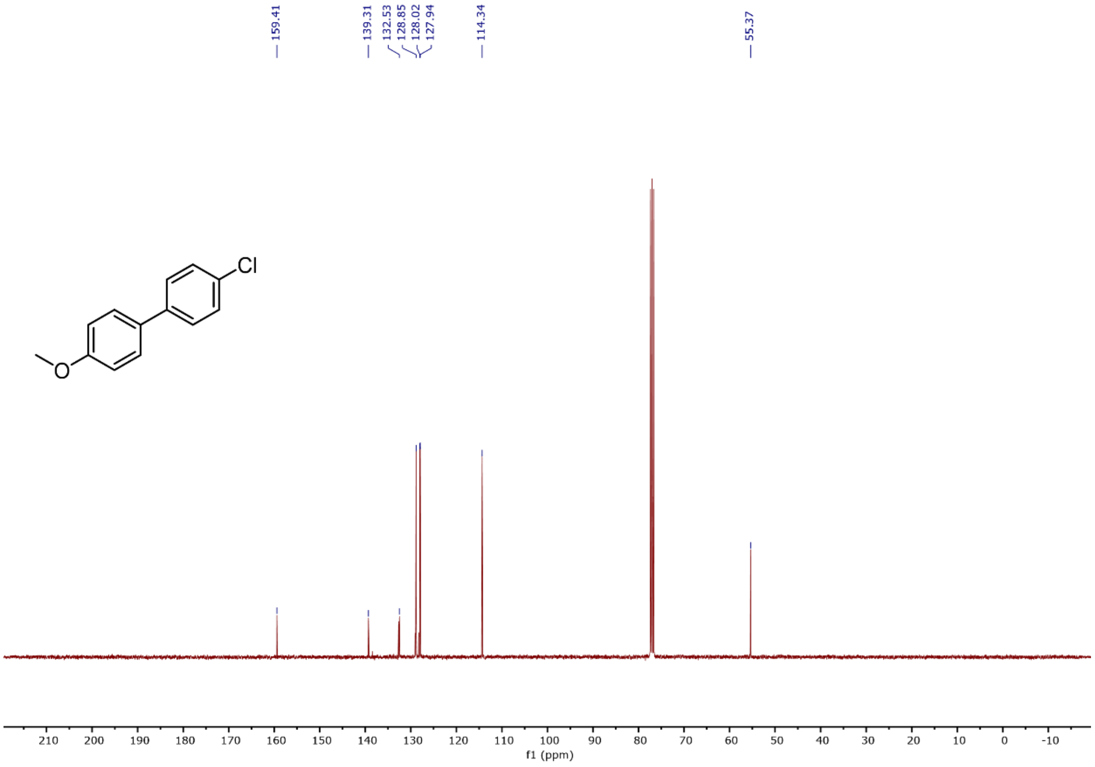


$^{13}$C NMR of *4-chloro-4'-methoxy-1,1'-biphenyl* **(4r)**

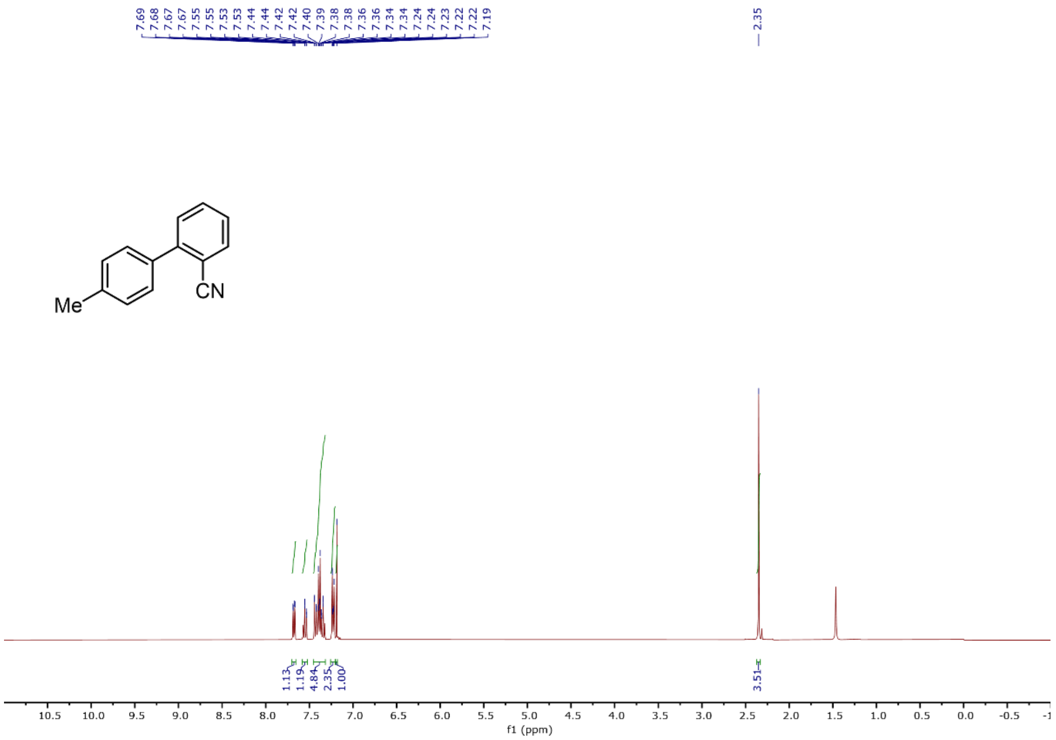

$^{1}$H NMR of *4'-methyl-[1,1'-biphenyl]-2-carbonitrile* **(4u)**

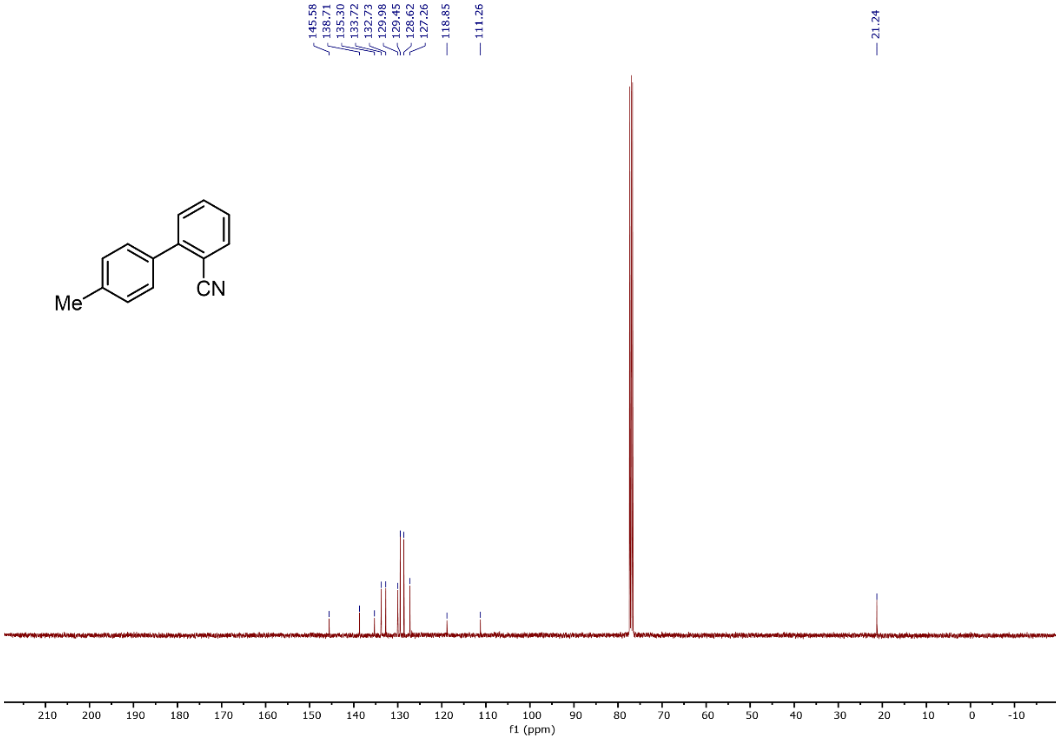

$^{13}$C NMR of *4'-methyl-[1,1'-biphenyl]-2-carbonitrile* **(4u)**

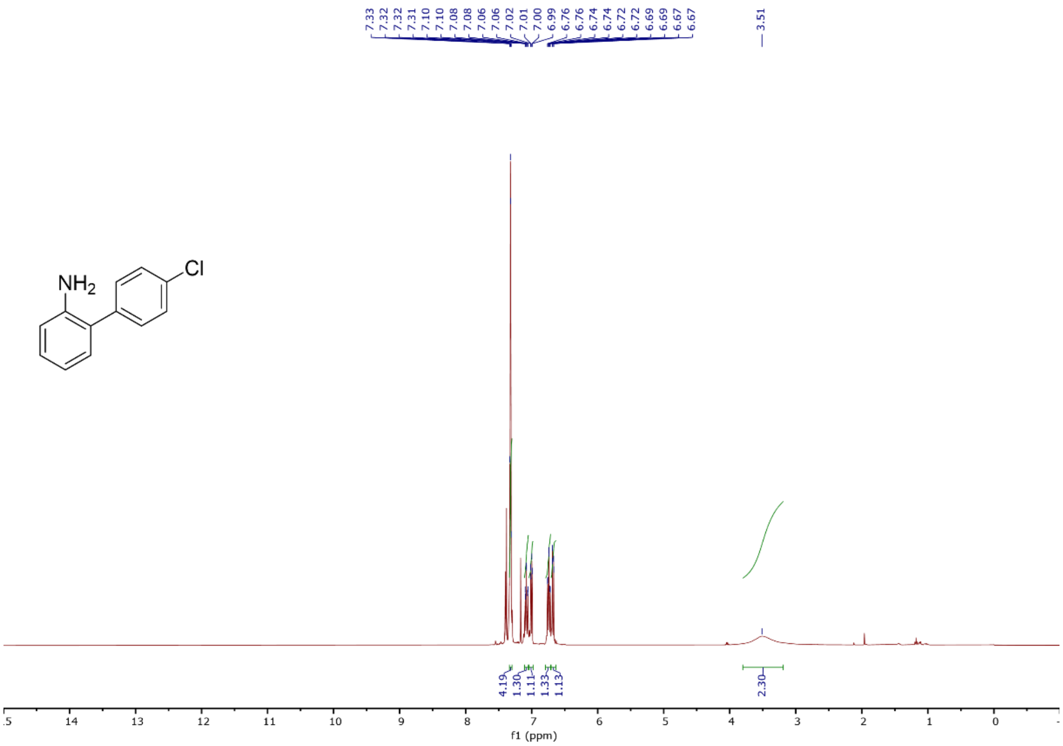

$^{1}$H NMR *4'-chloro-[1,1'-biphenyl]-2-amine* **(4t)**

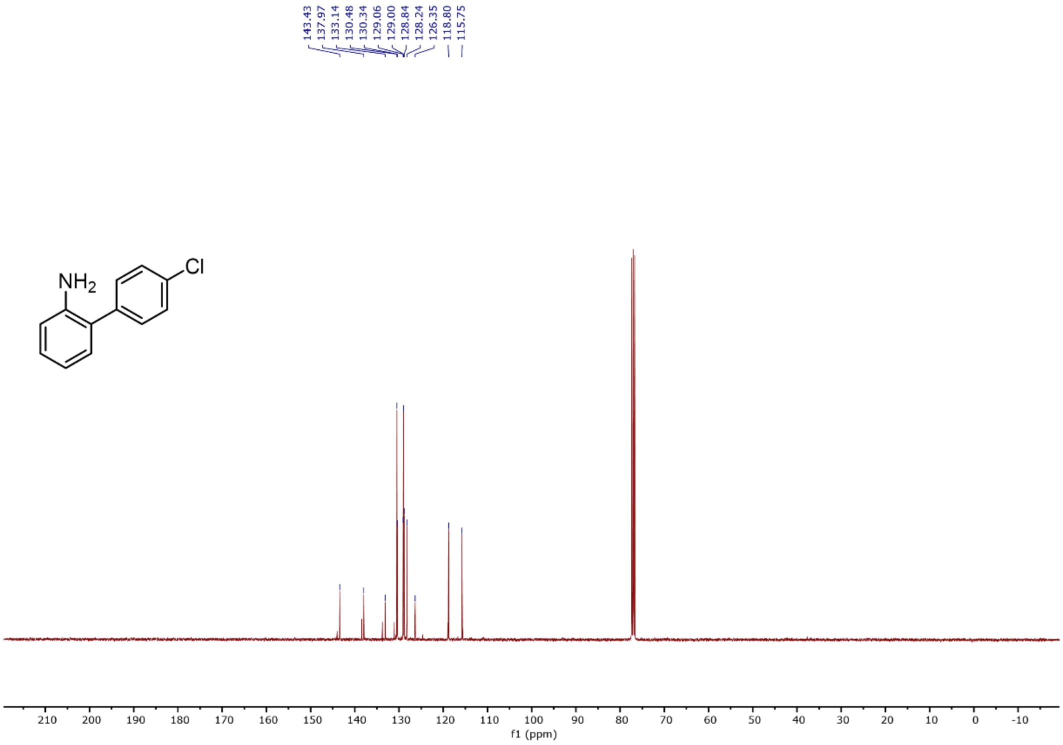

$^{13}$C NMR *4'-chloro-[1,1'-biphenyl]-2-amine* **(4t)**

## 8. References for the Supporting Information


[1] G. Di Liberto, L. Giordano, G. Pacchioni, *ACS Catal.* **2024**, *14*, 45–55.

[2] K. Chen, S. Zhang, P. He and P. Li, *Chem. Sci*. **2016**, *7*, 3676–3680.

[3] M. Cai, C. Luo, C. Xu and B. Huang, *Synthesis* **2022**, *54*, 1339–1346.

[4] F. D'Amico, C. Papucci, D. Franchi, G. Reginato, M. Taddei, A. Mordini, L. Zani, A. Dessì and M. Calamante, *J. Org. Chem*. **2024**, *89*, 6991–7003.

[5] A. Prakash, S. Basappa, B. Jeebula, D. H. Nagaraju, R. S. Dhayal and S. K. Bose, *Org. Lett*. **2024**, *26*, 2569–2573.

[6] C.-F. Meng, B.-B. Zhang, Q. Liu, K.-Q. Chen, Z.-X. Wang and X.-Y. Chen, *J. Am. Chem. Soc*. **2024**, *146*, 7210-7215.

[7] L. Whitaker, H. Y. Harb and A. P. Pulis, *Chem. Commun*. **2017**, *53*, 9364–9367.

[8] G. Vilé, D. Albani, M. Nachtegaal, Z. Chen, D. Dontsova, M. Antonietti, N. López, and J P-Ramírez, *Angew. Chem., Int. Ed*., 54 (38), 11265-11269.

[9] Ishiyama, T.; Murata, M.; Miyaura, N. *J. Org. Chem*. **1995**, *60* (23), 7508–7510.

[10] Murata, M.; Watanabe, S.; Masuda, Y. *J. Org. Chem*. **1997**, *62* (19), 6458–6459.

[11] Billingsley, K. L.; Barder, T. E.; Buchwald, S. L. *Angew. Chem., Int. Ed*. **2007**, *46*, 5359–5363.

[12] Molander, G. A.; Trice, S. L. J.; Dreher, S. D. *J. Am. Chem. Soc*. **2010**, *132* (50), 17701–17703.

[13] Zhang, Y.; Gao, J.; Li, W.; Lee, H.; Lu, B. Z.; Senanayake, C. H. *J. Org. Chem*. **2011**, *76* (15), 6394–6400.

[14] Tang, W.; Keshipeddy, S.; Zhang, Y.; Wei, X.; Savoie, J.; Patel, N. D.; Yee, N. K.; Senanayake, C. H. *Org. Lett.* **2011**, *13* (6), 1366–1369.

[15] Kawamorita, S.; Ohmiya, H.; Iwai, T.; Sawamura, M. *Angew. Chem., Int. Ed*. **2011**, *50*, 8363–8366.

[16] Molander, G. A.; Trice, S. L. J.; Kennedy, S. M.; Dreher, S. D.; Tudge, M. T. *J. Am. Chem. Soc*. **2012**, *134* (28), 11667–11673.

[17] Molander, G. A.; Trice, S. L. J.; Kennedy, S. M. *J. Org. Chem*. **2012**, *77* (19), 8678–8688.

[18] Dzhevakov, P. B.; Topchiy, M. A.; Zharkova, D. A.; Morozov, O. S.; Asachenko, A. F.; Nechaev, M. S. *Adv. Synth. Catal*. **2016**, *358*, 977–983.

[19] Zernickel, A.; Du, W.; Ghorpade, S. A.; Sawant, D. N.; Makki, A. A.; Sekar, N.; Eppinger, J. *J. Org. Chem*. **2018**, *83* (4), 1842–1851.

[20] Ratniyom, J.; Dechnarong, N.; Yotphan, S.; Kiatisevi, S. Eur. *J. Org. Chem*. **2014**, *2014*, 1381–1385.

[21] Tse, M. H.; Zhong, R.-L.; Kwong, F. Y. *ACS Catal*. **2022**, *12* (6), 3507–3515.

[22] Xu, L.; Dong, Z.; Zhang, Q.; Deng, N.; Li, S.-Y.; Xu, H.-J. *J. Org. Chem*. **2022**, *87* (21), 14879–14888.

[23] Niwa, T.; Takimoto, T.; Sakata, Y.; Hosoya, T. *Org. Lett*. **2023**, *25* (45), 8173–8177.

[24] Barroso, S.; Joksch, M.; Puylaert, P.; Tin, S.; Bell, S. J.; Donnellan, L.; Duguid, S.; Muir, C.; Zhao, P.; Farina, V.; Tran, D. N.; de Vries, J. G. *J. Org. Chem*. **2021**, *86* (1), 103–109.

[25] Compagno, N.; Lucchetti, N.; Palmisano, A.; Profeta, R.; Scarso, A. *J. Org. Chem*. **2024**, *89* (17), 12452–12461.

[26] D'Amico, F.; Papucci, C.; Franchi, D.; Reginato, G.; Taddei, M.; Mordini, A.; Zani, L.; Dessì, A.; Calamante, M. *J. Org. Chem*. **2024**, *89* (10), 6991–7003.

[27] Nelson, C. B.; L'Heureux, S. J.; Wong, M. J.; Kuhn, S. L.; Ghiglietti, E.; Lipshutz, B. H. *Green Chem*. **2024**, *26*, 10115–10122.
